# Maternal infection during pregnancy and the risk of childhood cancer: a systematic review and meta-analysis

**DOI:** 10.1186/s12916-026-04625-1

**Published:** 2026-01-14

**Authors:** Loviisa Mulanje, Lara Kim Brackmann, Alicia Lübtow, Hajo Zeeb, Wolfgang Ahrens, Manuela Marron, Rajini Nagrani

**Affiliations:** 1 Leibniz Institute for Prevention Research and Epidemiology – BIPS, Bremen, Germany; 2 Faculty of Human and Health Sciences, University of Bremen, Bremen, Germany; 3 Faculty of Mathematics and Computer Science, University of Bremen, Bremen, Germany

**Keywords:** Leukaemia, Childhood neoplasm, Cancer aetiology, Prenatal infections

## Abstract

**Background:**

Maternal infections during pregnancy may increase the risk of childhood cancer (CC) in offspring by affecting foetal immunity and genetics. Existing evidence seems inconclusive, necessitating a comprehensive review to understand this association. We aimed to evaluate the risk of various CC outcomes following prenatal exposure to different types of maternal infections.

**Methods:**

We searched Medline, Web of Science, Embase, Cochrane Library, and bibliographies for relevant studies from inception to October 2025. The study protocol was registered in PROSPERO (ID:CRD42023483706). We included original human epidemiological studies that examined the association between maternal infections during pregnancy and CC with appropriate reference groups and no language restrictions. We excluded studies if they were reviews or reports, if they did not assess individual-level infection or if they used therapies for infections (e.g., antibiotics) as markers of infection exposure. Two independent reviewers extracted data and assessed methodological quality following PRISMA guidelines. Pooled estimates (ES) and 95% confidence intervals (95%CIs) were calculated using random-effect models. Heterogeneity was examined in subgroup analyses. Publication bias was evaluated using Egger’s tests and Funnel plots.

**Results:**

From 9284 studies identified by the search, 46 studies (39 case–control,7 cohort) with over nine million participants were included, covering 33 analyses of 12 types of infection and five CC sites. Overall, maternal infection during pregnancy was associated with increased risk of CC (ES = 1.36;95%CI,1.17–1.59). Sexually transmitted infections (STIs) were associated with increased overall CC risk (ES = 2.86;95%CI,1.88–4.33). Viral infections were also associated with increased risk of overall CC (ES = 1.43;1.18,1.74), with cytomegalovirus and rubella virus infections showing positive associations upon stratification by pathogen-type. Genitourinary tract infections (GUTIs) were associated with increased risk of leukaemia (ES = 1.49;95%CI,1.05–2.12) and solid tumours (ES = 1.60, 95%CI;1.06–2.42) and viral infections with the risk of acute lymphoblastic leukaemia (ES = 1.58;95%CI,1.15–2.18).

**Conclusions:**

Maternal STIs, GUTIs, and viral infections during pregnancy are associated with increased risk of CC, with GUTIs and viral infections specifically associated with increased risk of leukaemia. Targeted prevention strategies towards specific infections during pregnancy may protect against CC. Large-scale prospective studies with precise infection assessment and stratification by pathogen-type, and mechanistic considerations are needed to deepen knowledge in this area.

**Supplementary Information:**

The online version contains supplementary material available at 10.1186/s12916-026-04625-1.

## Background

Cancer is a global health problem not only among older populations but also among children and adolescents with approximately 400,000 cases diagnosed every year in individuals aged 0–19 years [1]. The aetiologies of childhood cancer, remain largely unknown, despite being studied for decades. While rare genetic syndromes and high-dose ionising radiation are known risk factors for childhood cancer [2, 3], they only account for a small percentage of cases. Hence, further research is necessary to identify other factors associated with the development of cancers in childhood. As approximately half of childhood cancers are diagnosed before five years of age in utero exposures are of interest.

Maternal infections during pregnancy are common, with varying impacts on foetal development depending on the type of infection and the maternal and foetal immune response. Beyond the known adverse perinatal outcomes of pregnancy infections, such as low birth weight, intrauterine growth restriction, prematurity and congenital anomalies [4], emerging research suggests that infections during pregnancy might also be associated with cancer in the offspring. Infections during critical windows of foetal development may influence carcinogenesis through multiple mechanisms involving inflammation and immune modulation [5], with subsequent DNA damage or epigenetic modifications [6, 7]. Some viruses with known oncogenic potential, such as the Hepatitis B virus (associated with hepatocellular carcinomas), may cross the placenta and mediate direct effects on foetal genetics, ultimately influencing the risk of cancer in the offspring. Other infections, mainly ascending infections from the lower genital tract, may be vertically transmitted across the maternal-foetal membranes and may cause an altered foetal immune response [8]. In the absence of direct vertical transmission, infections may still trigger a foetal inflammatory response through the maternal metabolites and by-products of infection and also mediate epigenetic changes which might contribute to cancer susceptibility [7].

Despite suggestive findings of an association between infections during pregnancy and cancer in offspring from epidemiological studies in the last five decades, the evidence is limited and inconsistent, particularly concerning the pathogen type and specific cancer subtypes. A meta-analysis of 20 studies, published in 2019, found that influenza, varicella, and rubella infections of the mother during pregnancy were associated with an increased risk of childhood leukaemia [9]. However, this meta-analysis did not consider other pregnancy infections and was limited to leukaemia.

Therefore, to close this gap, we conducted a comprehensive systematic literature review and meta-analysis on the association between diverse maternal infections during pregnancy and the risk of various childhood cancers in the offspring.

## Methods

### Search strategy and selection criteria

**Fig. 1:**
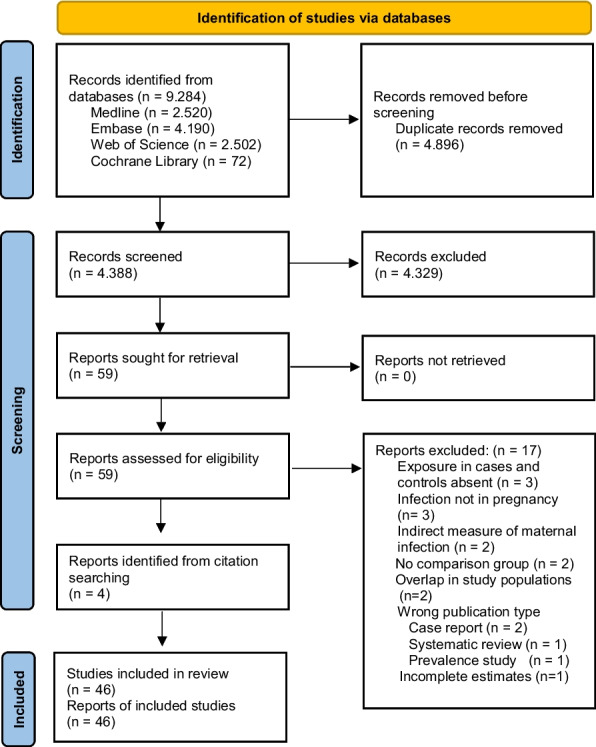
PRISMA 2020 flow diagram for the selection of studies

Our systematic review and meta-analysis was conducted in accordance with the Meta-analysis of Observational Studies in Epidemiology (MOOSE) [10] and the Preferred Reporting Items for Systematic reviews and Meta-analyses (PRISMA) [11] guidelines (Additional file 1: Table. S1) and was registered in PROSPERO (registration number: CRD42023483706). Two researchers (LM and LKB) systematically searched the Medline, Embase, Web of Science and Cochrane Library databases for articles published until October 2025, without language restrictions. We used subject headings and keywords in English, based on the search structure of the literature database, to combine terms related to the population (pregnant women and their offspring), exposure (prenatal infection), and outcome (childhood cancer). A detailed description of the search strategy, developed with the help of an experienced research librarian (LC), is provided in Additional file 2: Table. S2. The reference lists of the included studies were reviewed to identify additional relevant articles. Duplicate entries were removed using EndNote. Two reviewers (LM and LKB) independently screened the titles, abstracts and full-text articles. We included studies if they were human epidemiological research on maternal infections during pregnancy and their association with childhood cancer, with appropriate reference groups (controls without cancer; any other control definition critically assessed). Maternal infections were self-reported by mothers, abstracted from medical records, or assessed in biospecimens such as maternal blood during pregnancy or neonatal dried blood spots, with early neonatal infections considered indicative of congenital infections, acquired in utero. We excluded studies if they were reviews or reports, if they did not assess individual-level infection or if they considered antibiotics or other therapies for infections as exposure to infection. We only included studies with quantitative risk estimates (odds ratio (OR), relative risk (RR), or hazard ratio (HR)) or measures of variability (variance, standard error (SE), standard deviation (SD), confidence interval (CI)) or sufficient data to calculate crude risk estimates. We used the different quantitative risk estimates (OR, RR, HR) with the knowledge that they yield similar results in rare outcomes such as childhood cancer [12]. For studies with overlapping populations, the most recent publication was selected. Disagreements regarding the review of the articles were resolved by consensus with other authors (RN and MM). A detailed description of the study selection is shown in Fig. 1.

### Data extraction and quality assessment

Two of us (LM and LKB) extracted data from each publication as follows: first author name, study design, location, period, number of cases, reference group details, cancer outcome, age at diagnosis, type of infection, method of infection and outcome assessment, matching and adjustment factors, statistical approaches, and risk estimates. Immunoglobulin M (IgM) was selected as a marker of recent infection when multiple immunoglobulins were reported and the model with the most confounder adjustments was used to minimize confounding where multiple adjustment models were reported (Additional file 3: Table. S3).

We assessed the quality of the studies using a scoring system adapted from the Newcastle–Ottawa Scale (NOS) and Risk Of Bias in Non-randomized Studies of Exposure (ROBINS-E) and of Intervention (ROBINS-I) tools. This scoring system expands on the original NOS, recognizing the NOS’s limitation in extensively appraising observational studies, and includes the risk of bias considerations from ROBINS. The tool is described elsewhere [13]. Briefly, it consists of 8 categories with basic study-level characteristics and elements for risk of bias assessment, with a maximum quality score of 45 points. Studies are classified studies as high- or low-quality using a cut-off at the 4th quintile score. Two authors (LM and LKB) independently extracted quality-related items from the included studies, and a third author (RN) resolved disagreements. Detailed quality criteria are available in Table 2.

### Statistical analysis

Pooled estimates (ES) with 95% confidence intervals (CIs) were calculated using random-effects models when at least three studies investigated the same exposure–outcome relationship (i.e., maternal infection during pregnancy and a specific childhood cancer type) [14]. We conducted a total of 33 analyses, examining the association between maternal infections during pregnancy and childhood cancer, focusing on infections and cancers with sufficient data (at least three studies per analysis) for evaluation. To provide an initial summary estimate of the overall association between maternal infections during pregnancy and risk of childhood cancer, we first conducted an overall meta-analysis including all eligible studies. This broad analysis was intended to offer a general overview before performing stratified analyses by type of infection and by specific childhood cancer type. For specific infections, we analysed infections by pathogen type (bacterial and viral), by affected body site (respiratory tract, urinary tract, and genitourinary tract infections (GUTIs)) and by mode of transmission (sexually transmitted infection (STIs)). For cancer types, we analysed leukaemia (all histological subtypes together and acute lymphoblastic leukaemia (ALL) separately), lymphoma (all subtypes collectively due to insufficient data (less than three studies) for individual subtypes), central nervous system tumours (CNS) and neuroblastomas, and solid tumours (including germ-cell tumours, hepatic tumours, hepatoblastomas, retinoblastomas, testicular cancers, and Wilms tumours). Solid tumours were analysed as a group also due to insufficient data for individual tumour types. One study did not specify the type of infection and was included under the “any infection” category [15]. When multiple infection types or cancer outcomes were reported, we selected the most general and prevalent infection and cancer type. For instance, in the study by Roman et al. [16], which examined both general and specific viral infections in relation to leukaemia and non-Hodgkin lymphoma, we included general viral infection and leukaemia as the representative exposure and outcome respectively.

We quantified heterogeneity using the *I*^*2*^ statistic, categorized as low (≤ 50%), moderate (> 50–75%), or substantial (> 75%), with its statistical significance assessed using the Q statistic (*p* value for heterogeneity, (*p*)). To explore potential effects on heterogeneity, we conducted sensitivity analyses by omitting studies one at a time for each analysis [17]. We also conducted subgroup analyses stratified by study characteristics, including study design (case–control or cohort), type of infection assessment (self-reported data, medical records, or biological specimens), type of outcome assessment (cancer registry/pathological confirmation), confounder control (adjustment for basic factors, additional factors, or other infections), geographic region (Asia and Oceania, Europe, and North America), study period (pre- or post-2000), and overall study quality (high or low). In addition, we conducted meta-regression analyses to examine the influence of these study-level characteristics on the effect estimates to further identify and explain potential sources of heterogeneity. The statistical significance of each covariate was evaluated using a p-value threshold of 0.05. For meta-analyses including ≥ 5 studies we evaluated whether publication bias was present by visually inspecting a funnel plot and applying Egger’s asymmetry test [18]. A significant Egger’s test (p < 0.05) and/or asymmetry in the funnel plot were considered indicative of publication bias. In such cases where evidence of publication bias was observed, we applied the Duval and Tweedie trim-and-fill method to estimate the potential effect of missing studies on the pooled results [19]. All analyses were performed in Stata version 18 (StataCorp LP, College Station, TX, USA). 

## Results

### Study characteristics and quality of included studies

We identified 9,284 articles from the four databases with 4,896 duplicates removed and 4,329 excluded based on title and abstract screening, leaving 59 articles for full-text evaluation (Fig. 1). An additional four articles were identified from references in these 59 papers. Seventeen full-text articles were excluded based on our exclusion criteria (Additional file 4: Table. S4), leaving 46 studies for the review and meta-analysis [15, 16, 20–63]. Three studies were excluded from the meta-analysis: two because they were based on overlapping study populations [64, 65] and one because it did not report estimates of risk [66].

The 46 studies included in the meta-analysis were published between 1958 and 2025 and were conducted in Asia or Oceania (13%), Europe (50%), and North America (37%), with sample sizes ranging from 90 to 2,782,507 participants per study (Table 1). Most studies focused on cancer diagnoses within the first 18 years of life, with some exceptions extending to 20, 30, or 40 years of age [16, 30, 56].

**Table 1:** Characteristics of studies included in meta-analysis

First Author, Year	Location	Study years	No. of Cases	No. of Controls	Age Range	Outcome	Infection	Results	Outcome Assessment	Exposure Assessment	Study Design	Comment	Quality
Birch, 1990 [20]	England (North Western, West Midlands, Yorkshire)	1980–1983	78	156	< 15 years	Central Nervous System	Chickenpox, measles, influenza, UTI, "other" infections	No association	Registry	Self-reported questionnaires with some verification with medical records	Population-based Case–Control	Mat: age, sex; hospital controls without neoplastic disease, genetic, constitutional disease, malformation or chronic disease	22.6
Bithell, 1973 [21]	UK (England, Wales, Scotland)	1953–1967	9000	9000	≤ 16 years	Leukaemia, other lymphatic tissue disease, other tumours	Influenza, chicken pox, rubella, other viral infections	Increased risk after influenza and chickenpox infection	Registry	Self-reported interviews and antenatal records where possible	Population-based Case–Control	Cancer deaths; Mat: sex, date of birth	24.8
Blot, 1980 [22]	UK (England, Wales, Scotland)	1971–1976	2823	2484	≤ 15 years	Childhood cancer (leukaemia, brain tumours, neuroblastoma, ovarian cancer)	Chickenpox	No association	Registry	Self-reported interviews and antenatal records where possible	Population-based Case–Control	Cancer deaths; Mat: age, sex, nearness of residence to case child	23.6
Bogdanovic, 2016 [23]	Sweden	1992–2006	95	95	≤ 17 years	ALL	HHV6, Human Parvovirus B19, HERV (DNA)	No association	Registry	Unbiased NGS on neonatal blood spots NBS	Population-based Case–Control	Mat: age and birthplace	18.4
Bonaventure, 2025 [61]	England and Wales	1991–1998	2885	5499	< 15 years	leukaemia, lymphoma, CNS tumours, embryonal tumours or other	Any infection, urinary tract infection, genital infection, influenza, chickenpox	Increased risk of lymphoma after urinary tract infection	Pathological confirmation and Registry	Medical records	Population-based Case–Control	Adj: age, year of birth, General practitioner record abstraction status Mat: age and sex	27.6
Bunin, 1987 [24]	USA (Philadelphia)	1970–1983	88	88	< 15 years	Wilm's tumour	Vaginal infection	Increased risk	Pathological confirmation	Telephone interviews on exposures in the year prior to index pregnancy	Population-based Case–Control	Adj: demographical differences between cases and control and risk factors; Mat: race, birth date, telephone area code and exchange	22.6
Bzhalava, 2016 [25]	Sweden	1977–2005	47	47	≤ 15 years	Childhood leukaemia and lymphoma	Anelloviridae, "unclassified viruses", environmental viruses, papillomaviridae	No association	Registry	NGS of maternal first trimester (14 weeks gestation) sera	Nested Case–Control	Mat: gender, date of birth of child (± 2 months), mother’s age at serum sampling (± 2 years), date at serum sampling (± 2 months) and alive and free from leukaemia/lymphoma at the time of index child diagnosis	23.6
Dockerty, 1999 [26]	New Zealand	1990–1995	121	303	< 15 years	Childhood leukaemia	Influenza, cystitis or kidney infection, cold sores/oral herpes, any other infection (rubella, measles, chicken pox, shingles, mumps, glandular fever, pneumonia, hepatitis B, malaria, leptospirosis)	No association	Pathological confirmation	Self-reported questionnaire on maternal infection in pregnancy or in the 3 months before the pregnancy	Population-based Case–Control	Adj: age, sex, child’s social class, household crowding, mother’s educational level; Mat: age, sex	24.2
Farwell, 1979 [27]	USA (Connecticut)	1956–1962	52	38	≤ 19 years	CNS Tumours	SV40 virus in polio vaccine	Increased risk of medulloblastoma after antenatal exposure to SV40	Registry	Questionnaires to obstetricians on polio vaccination during pregnancy	Population-based Case–Control	Mat: gender, date of birth, town of birth	17.0
Fear, 2001 [28]	UK	1956–1992	83	166	< 15 years	Primary malignant neoplasm of the brain or other part of the nervous system	Definite & probable Viral infection—defined by ICD-10 (rubella, mumps, varicella, herpes zoster)	Increased risk after infection of definite viral origin during pregnancy; no association with probable viral infection	Registry	Maternal obstetric notes on nursing cards at the study hospitals	Population-based Case–Control	Adj: age, sex; Mat: birth, sex, month, year of birth	21.6
Fedrick, 1972 [29]	UK (England, Wales, Scotland)	1958–1969	20	16,730	≤ 11 years	Childhood cancer	Influenza	Increased risk	Registry and Individual physicians	Self-reported questionnaires on exposure during pregnancy	Retrospective cohort	Adj: birth date	23.4
Fine, 1985 [30]	UK	1950–1972	2570	2475	≤ 40 years	Malignant disease (ICD codes (9th revision) 140–208; 230–234	Viral infection (influenza, varicella, herpes zoster, mumps, rubella, measles, hepatitis, CMV, miscellaneous viruses)	Increased risk of cancer among those exposed to herpes viruses, specifically varicella and cytomegalovirus	Death certificates, questionnaire to general practitioner	self-reported interviews or medical records from general practitioner	Cohort study	Mat: sex and date and area of birth	24.6
Francis, 2017 [31]	USA (California)	1982–2006	268	270	≤ 18 years	ALL	CMV and EBV	Increased risk of ALL with CMV infection; no association with EBV	Registry	Droplet digital PCR on NBS	Population-based Case Control	Mat: date of birth, race, sex	25.6
Gardner, 1990 [32]	UK (West Cumbria)	1950–1985	97	1001	≤ 25 years	Leukaemia, NHL, HL	Viral infections (chickenpox, influenza, rubella)	No association	Hospital records, death certificates, registries and pathological review	Self-reported questionnaires, obstetric records, general medical records	Population-based Case Control	Mat: sex, date of birth	24.4
Geris, 2023 [62]	USA	1987–2014	1189	4756	< 15 years	ALL	CMV	No association for overall ALL; increased risk of hyperdiploid ALL	Pathological confirmation	PCR on NBS	Population-based Case Control	Adj: mother’s age at birth, maternal diabetes, birth weight, categorical gestational age, and presence of congenital anomaly Mat: year of birth, sex, and mother’s reported race and ethnicity	33.4
Gustafsson, 2007 [33]	Sweden	1980–2001	49	47	≤ 17 years	ALL	C adenovirus DNA	Increased risk	No information	Nested PCR on NBS	Nested Case–Control	Mat: birth date and birthplace	19.2
Hamrick, 2001 [34]	USA and Canada	1992–1994	504	504	< 19 years	Neuroblastoma	Hepatitis, rubella, measles, mumps, influenza, chickenpox or shingles, mononucleosis, other infectious diseases, UTI, STI	Increased risk with chickenpox and STI	Pathologically confirmed cases from National Cancer Institute-sponsored paediatric collaborative clinical trials groups, the Children’s Cancer Group (CCG) and the Paediatric Oncology Group (POG)	Self-reported interviews	Population-based Case–Control	Adj: child’s gender, mother’s race and education, and household income in the birth year; Mat: date of birth	25.4
He, 2023 [35]	Denmark	1978–2015	4362	2,218,435	< 15 years	Any childhood leukaemia, ALL, AML, other childhood cancers, including brain tumours, lymphoma, and other cancers	Any infection, respiratory tract, genitourinary tract (i.e. urinary and genital tract), digestive system infection, sexually transmitted infections, other infections	Increased risk of childhood leukaemia after any maternal infection, genital tract infection, UTI and STI; no association between maternal infection and childhood brain tumours, lymphomas, or other cancers	Registry	Registry	Prospective cohort	Adj: age at childbirth, education level, parity, cohabitation during pregnancy, diabetes during pregnancy (including pre-pregnancy diabetes and gestational diabetes), year of delivery, and season of deliver (spring, summer, autumn, or winter) maternal comorbidities during pregnancy: hypertensive disorders, anaemia, obstetric haemorrhage, hyperemesis, and asthma	30.4
Heck, 2012 [36]	USA	1988–2007	609	209,051	< 6 years	Retinoblastoma (Bilateral and Unilateral)	Sexually transmitted infection (STI) (genital herpes, syphilis, chlamydia, gonorrhoea)	Increased risk of bilateral retinoblastoma after STI in pregnancy	Pathological confirmation	Birth certificates	Population-based Case Control	Adj: year of birth, father's age, urban or rural country of residence, mother's race and birthplace; Mat: year of birth	31.2
Heck, 2022 [38]	Taiwan	2004–2015	2160	2,076,877	< 11 years	All cancers	Hepatitis B and C	Increased risk of all childhood cancers after Maternal HCV infection	Registry	National health insurance research database	Retrospective cohort	Adj: birth year, parental age and child’s viral hepatitis status, family income, urban or rural area of residence, parity, child’s sex, parental HIV infection, other parent’s viral hepatitis status and maternal country of birth	32.4
Heck, 2015 [37]	USA and Canada	2006–2011	252	136	< 15 years	Retinoblastoma	Any infectious disease, respiratory infection, flu or cold, STI, other viral infections (hepatitis B and C, shingles, HPV, herpes, stomach virus, Murray infection, and Fifth disease), all bacterial infections, UTI	No association	Medical documentation—hospital or by the Children’s Oncology Group (COG)	Self-reported phone interviews	Case–control	Adj: age, biological relative status of parents, child’s age at interview, mother’s race/ethnicity, mother’s educational attainment, household income, mother’s age at birth, and maternal smoking in the month before or during pregnancy; Mat: age and biological relative status of parents	16.2
Heinonen et al. 1973 [39]	USA	1959–1966	24	50,867	≤ 4 years	Malignant neoplasms	Spontaneous viral infection—not specified	No association	Pathological confirmation	Self-reported interview, review of hospital and other records and confirmation by attending physicians whenever possible	Prospective cohort	no adjustment	23.2
Holl, 2008 [40]	Finland, Sweden, Iceland	1975–2001	66	258	0—26 years	Testicular cancer	EBV and CMV (IgM, IgG)	Increased risk of TC with high level of maternal EBV IgG; Decreased risk of TC with CMV IgG positivity	Pathological confirmation	First trimester maternal serum IgM and IgG	Nested Case–Control	Mat: date of birth of son (± one month)	24.8
Honkaniemi, 2010 [41]	Sweden	1992–2006	243	484	≥ 67 days—≤ 15 years	ALL	C adenovirus (DNA)	No association	Registry	Nested PCR in NBS 3–5 days of age	Nested Case–Control	Mat: birthplace and birthdate	21.2
Kumar, 2014 [15]	India	2008–2012	132	132	< 18 years	Leukaemia—ALL and AML	Any infection—not specified	No association	Medical documentation	Self-reported interviews on exposure during pregnancy	Population-based Case Control	Mat: age, sex, residency	16.8
Kwan, 2007 [42]	USA	1995–2002	365	460	< 15 years	Leukaemia, ALL and c-ALL (Common ALL)	Influenza/pneumonia, STI, UTI	Increased risk of childhood leukaemia and ALL with influenza/pneumonia and Increased risk of childhood leukaemia with STI	Registry	Self-reported interviews on exposure during 3 months before pregnancy and during pregnancy	population-based Case Control	Adj: household income, maternal education, maternal age at birth of child, antibiotic use; Mat: date of birth, sex, Hispanic ethnicity, maternal race, maternal county of residence at birth	24.6
Lehtinen, 2003 [43]	Finland and Iceland	1975–1997	403	1216	< 15 years	Leukaemia	EBV, CMV, HHV6 (IgG and IgM)	Increased risk of childhood leukaemia and ALL with maternal EBV	Pathological confirmation	Maternal serum IgG and IgM antibodies at 12–14 weeks gestation	Nested Case–Control	Adj: birth order, sibship size; Mat: age at serum sampling, date of specimen collection, date of birth and sex of offspring, country	30.6
Lehtinen, 2005 [44]	Finland and Iceland	1975–1997	402	1212	≤ 15 years	Leukaemia	Chlamydia, Helicobacter pylori, Mycoplasma pneumoniae in first trimester	Increased risk of childhood leukaemia with H. pylori IgG	Pathological confirmation	Maternal serum IgG and IgM antibodies at 12–14 weeks gestation	Nested Case–Control	Adj: birth order, sibship size; Mat: age at serum sampling, date of specimen collection, date of birth and sex of offspring, country	30.6
Linos, 1998 [45]	Greece	1982–1993	94	210	≤ 17 years	Brain tumours and neuroblastomas	Influenza	Increased risk of brain tumours and neuroblastoma	Pathological confirmation	Self-reported interviews—(influenza diagnosis based on response regarding physician visit)	Hospital-based Case–control	Adj: maternal smoking during pregnancy, paternal smoking prior to birth of index child, age of index child	23.4
Ma, 2021 [46]	China	2012–2020	80	160	< 17 years	Solid tumours	Common cold	No association	Medical documentation	Self-reported questionnaires	Case–control	Mat: birth weight, gestational age, residence, and pregnancy BMI	21.4
McKinney, 1999 [47]	Scotland	1991–1994	390	716	≤ 14 years	Leukaemia, lymphomas, CNS tumours and other solid tumours	Any infection, respiratory tract infection, viral infection, genitourinary infection, fungal infection	Increased risk of "other solid tumours" with respiratory tract infections	Pathological confirmation	Mother's hospital obstetric records	Population-based Case–Control	Mat: age (to within one calendar month), sex and health board area of residence	24.2
Naumburg, 2002 [48]	Sweden	1973–1989	652	652	≤ 16 years	Childhood leukaemia (lymphatic and myeloid leukaemia)	Lower genital tract infections, UTI, ‘‘other infections’’ (such as pneumonia, gastroenteritis, sinusitis, common cold)	Increased risk of childhood leukaemia with lower genital tract infection	Registry	Antenatal, obstetric, and other standardized medical records	Population-based Case–Control	Adj: mothers age, parity, mode of delivery (vaginal or caesarean), maternal smoking, elapsed time from rupture of the membranes to delivery, gestational age at birth, birth weight, birth weight for gestational age, and type of birth (single or multiple); Mat: sex and year and month of birth	26.6
Oksuzyan, 2013 [49]	USA	1988–2008	3308	3308	< 16 years	CNS and brain tumours	Genital herpes and infections (non-sexually transmitted -including pyelonephritis, hepatitis B and rubella)	Increased risk of CNS Tumours with genital herpes; Decreased risk with non STI	Registry	Registry	Population-based Case–Control	Adj: child's race, birth weight, gestational age, birth order, mother's age, father's education and source of payment for delivery; Mat: date of birth (± 6 months) and sex -cancer free	27.4
Olshan, 1993 [50]	USA	1984–1986	200	233	≤ 15 years	Wilm's Tumour	Venereal disease, vaginal or UTI, rubella, measles, flu, mononucleosis, chickenpox, genital herpes, or “other infections during pregnancy"	Increased risk with "other infections”; no association found for the rest	Pathological confirmation	Self-administered questionnaire	Population-based Case- Control	Adj: household income and father's education; Mat: age at diagnosis and geographic area	24.4
Roman, 1997 [16]	UK	1962–1992	177	354	3 months ≤ 29 years	leukaemia and NHL	Viral infection, influenza, vulvar warts, herpes simplex, rubella	Increased risk of leukaemia after viral infection; Increased risk of non-Hodgkin's lymphoma after viral infection	Registry	Mother's hospital obstetric records	Population-based Case Control	Mat: hospital catchment area of birth, sex and year and month of birth	22.6
Sepúlveda-Robles, 2025 [63]	Mexico	2010–2015	1455	1455	< 18 years	Acute leukaemia	Any infection, respiratory infection, gastrointestinal infection, UTI, vaginal infection	Reduced risk of leukaemia with respiratory infection and UTI	Pathological confirmation	Self-reported standardized questionnaire	Hospital-based case control	Adj: child’s sex, age, birth weight, birth order, and standard of living Mat: age and health institution	26.6
Shu, 1995 [51]	USA, Canada	1982–1989	105	639	< 15 years	Malignant Germ Cell Tumours	Any virus infection, UTI, other infection	Increased in risk of MGCT with UTI	Registry	Self-administered questionnaire	Population-based Case–Control	Adj: age, gender, gestational age, number of livebirths, maternal education, maternal smoking in pregnancy; Mat: area and age	24.6
Sirirungreung, 2024 [52]	Taiwan	2004–2015	1409	2,265,777	< 15 years	ALL, CNS tumours, Hepatoblastomas and Medulloblastomas	Medically diagnosed infection—grouped based on pathogen type (viral or bacterial) and affected organ system (such as the respiratory, enteric or urinary tract)	Increased risk of ALL after any infection	Registry	Insurance data	Cohort study	Adj: sex, birth year, maternal age, family income, urbanization level, parity	28.4
Stewart, 1958 [53]	UK	1955–1957	1299	1299		Leukaemia, other cancers	Infections of genitourinary tract, rubella, mumps, herpes zoster, infective hepatitis	No association	Registry	Interviews by survey doctors on exposure during pregnancy	Population-based Case–Control	Mat: age, sex, locality, live healthy child	20.6
Stolt, 2005 [54]	Finland	1983	115	918		Neuroblastoma	Polyomavirus BK and JC virus (Specific IgG and IgM antibodies to BK and JC virus & PCR for BK virus DNA)	No association	Registry	First trimester sera: Serology—Specific IgG and IgM antibodies to BK and JC virus & Virology: PCR for BK virus DNA	nested case–control	Adj: age at serum sampling; Mat: maternal age at serum sampling and municipality of residence	22.0
Swerdlow, 1982 [55]	UK	1953–1973	87	10,128	< 16 years	Testicular Cancer	Cystitis, renal infections, any non-venereal UTI, tuberculosis	Increased risk of cancer death with tuberculosis and cystitis in pregnancy	Registry	Interviews and antenatal records where possible on exposure during pregnancy	Population-based Case Control	no matching/adjustment	20.2
Tedeschi, 2009 [56]	Finland and Iceland	1983–2006	705	2105	≤ 20 years	ALL and non-ALL	EBV (VCA IgM, early antigen IgG and IgM, ZEBRA IgG and IgM)	No association	Registry	First-trimester maternal serum antibodies	Nested Case–Control	Adj: birth order, sibship size Mat: mother's country, age at serum sampling, date of specimen collection and, for the offspring characteristics, date of birth (2 months) and gender of the child	28.4
Van Steensel-Moll, 1985 [57]	Netherlands	1973–1980	519	507	< 15 years	ALL	Viral infections—not specified	No association	Pathological confirmation	Self-administered questionnaire	Population-based case control	Adj: age and sex Mat: date of birth, sex, place of residence	26.0
Vasconcelos, 2008 [58]	USA, California	1995–2002	89	100	< 15 years	ALL	Adenovirus DNA	Virus not detected in cases	Registry	PCR on neonatal blood spots	Population-based Case Control	Mat: birthdates, gender, ethnicity and geographic region of birth	18.0
Wang, 2019 [59]	China	2014–2016	345	345	≤ 15 years	ALL	Viral infections—rubella, measles, cytomegalovirus, toxoplasma infection, but not including common viral infections like colds and influenza	No association	Pathological confirmation	self-reported questionnaire by trained interviewer	Hospital-based Case–control	Adj: child’s age, gender, residence region, birth weight, delivery mode, household per capita annual income, familial history of leukaemia and other cancer, exposure to pesticides, parental age at delivery, parental educational level, paternal exposure to pesticides, paternal cigarette smoking and paternal heavy alcohol drinking; Mat: age, gender, residency	25.6
Wiemels, 2019 [60]	Sweden	1987–2014	3198	2,779,309	< 15 years	Haematological malignancies and CNS tumours	Clinically apparent CMV	Increased risk	Registry	Registry	Retrospective cohort	Adj: congenital malformations, deformations, chromosome abnormalities, and small or large for gestational age	28.2

Study quality assessed using the self-developed scoring tool from −6 to 45 score

*Abbreviations*: *Adj* adjusted factors, *Mat* matching factors, *ALL* acute lymphoblastic leukaemia, *CMV* cytomegalovirus, *CNS* central nervous system, *EBV* Epstein Barr virus, *HERV* Human endogenous retrovirus, *HHV* human herpes virus, *IgG* immunoglobulin, *IgM* immunoglobulin, *MGCT* malignant germ-cell tumours, *NHL* non-Hodgkin lymphoma, *PCR* polymerase chain reaction, *UTI* urinary tract infection, *STI* sexually transmitted infection

The distribution of selected quality-related factors is shown in Table 2. The majority of the 46 papers were case–control studies (n = 39), which included eight nested, 27 population-based, and four hospital-based case–control studies. The remainder were cohort studies (n = 7). For outcome assessment, over half (57%) used registry data [16, 20–23, 25, 27–29, 31, 35, 38, 41, 42, 48, 49, 51–56, 58, 60], 37% used pathological confirmation [20, 24, 26, 34, 36, 39, 40, 43–45, 47, 50, 57, 59, 61–63] and nine percent used medical documentation [15, 30, 37, 46]. For exposure assessment, 74% used non-laboratory data: interviews and self-completed questionnaires [15, 24, 26, 27, 29, 34, 37, 42, 45, 46, 50, 51, 53, 57, 59, 63], medical documentation (antenatal or obstetric records, birth certificates) [16, 20–22, 28, 30, 32, 36, 38, 47, 48, 55, 61], registry data [35, 49, 60], and health insurance data [38, 52]. The remaining 26% of studies used laboratory data (DNA or IgM) [23, 25, 31, 33, 40, 41, 43, 44, 54, 56, 58, 62]. Over 95% controlled for basic confounders only (age, sex, place of residence) while only a few considered more confounders: hepatitis in pregnancy [37], prenatal antibiotic use [42], maternal comorbidities in pregnancy [35] and perinatal factors [37, 42, 45, 46, 48, 49, 51, 59, 60].

**Table 2:**
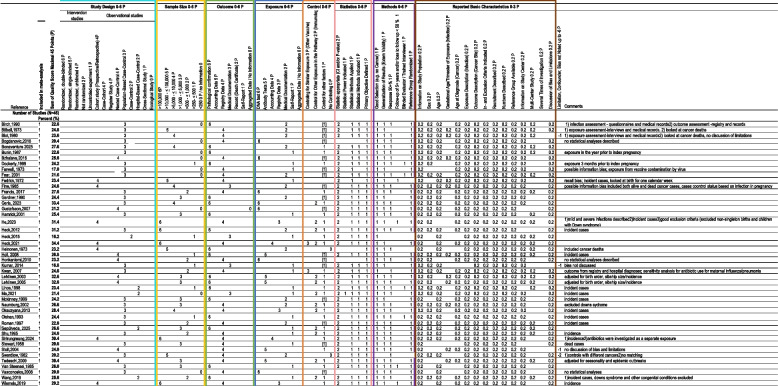
Quality assessment of studies included in the meta-analysis [15, 16, 20–33, 35–37, 39–48, 50–53, 55–63]

### Meta-analysis

**Fig. 2:**
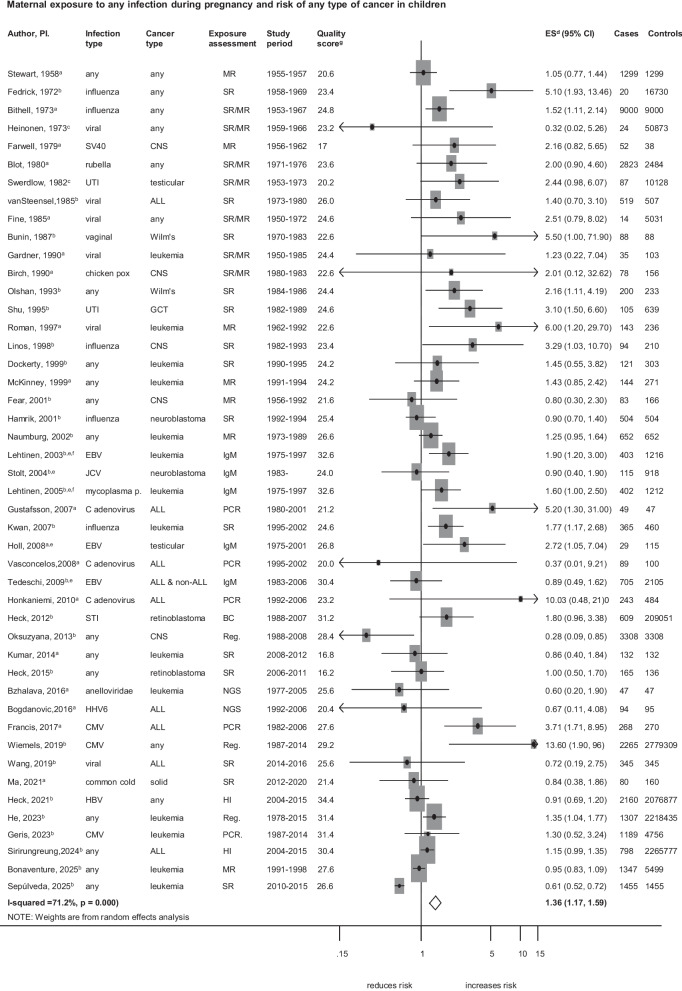
Maternal exposure to any infection during pregnancy and the risk of any type of cancer in children Overall analysis with all studies included in the meta-analysis. Exposure and outcome selection from individual studies depends on broad or general types and a larger number of cases. **a** crude odds ratio taking matching into account. **b** Adjusted estimate as indicated by the published study. **c** no adjustment/matching. **d** ES includes single-study odds ratios or hazard ratios and summary odds ratios. **e** first-trimester maternal serum samples. **f** studies likely to be from the same population. **g** quality score, high quality ≥ 28.4, low quality < 28.4. Abbreviations: ALL, acute lymphoblastic leukaemia; BC, birth certificate; ES, estimate; CI, confidence interval; CNS, central nervous system; EBV, Epstein Barr virus; GCT, germ cell tumour; HHV6, human herpes virus 6; HI, health insurance; IgM, Immunoglobulin M; JCV, human polyomavirus 2; MR; medical records; mycoplasma p., mycoplasma pneumoniae; NGS, next generational sequencing on neonatal blood spots; PCR, polymerase chain reaction on neonatal blood spot; Reg, registry data; SR, self-report; STI, sexually transmitted infection; SV40, simian virus 40; UTI, urinary tract infection. Weights are from random effect analysis

The overall analysis showed that maternal infection during pregnancy was associated with an increased risk of childhood cancer (ES = 1.36; 95%CI, 1.17–1.59; *I*^2^ = 71.2%; *p* < 0.001; Fig. 2)**.** We observed evidence of publication bias in this analysis; after correcting for publication bias using the trim-and-fill method, four additional studies were added, yielding ES = 1.32; 95%CI, 1.13–1.54) (Additional file 5: Fig. S1). Excluding single studies did not substantially alter the pooled estimate (Additional file 6: Table. S5). Subgroup and meta-regression analyses by study-level characteristics on confounder adjustment, exposure and outcome assessment methods gave similar results in terms of strength and direction of association (Additional file 7: Table. S6 and Additional file 8: Table. S7). However, the effect estimates differed when the analyses were stratified by study period. For example, we did not observe a positive association in the subgroup of recently conducted studies (P_meta-regression_ = 0.014; Additional file 8: Table.S7). When restricted to high-quality studies (n = 10), the association remained positive (ES = 1.25; 95% CI, 0.99–1.58), although it did not reach statistical significance (Additional file 7: Table. S6).

**Fig. 3:**
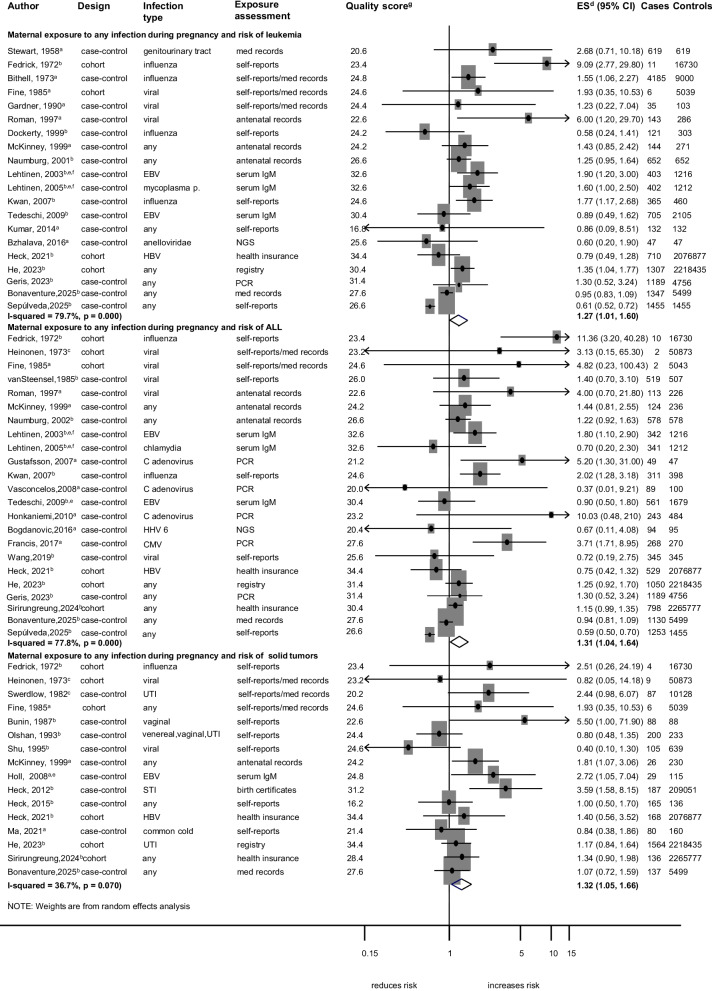
Maternal exposure to any infection during pregnancy and risk of leukaemia (all subtypes), ALL and solid tumours. Leukaemia includes all subtypes; solid tumours include Wilms tumours, retinoblastomas, hepatoblastomas, malignant germ cell tumours, and testicular cancers. **a** crude odds ratio taking matching into account. **b** Adjusted estimate as indicated by published study. **c** no adjustment/matching. **d** ES includes single-study odds ratios or hazard ratios and summary odds ratios. **e** first-trimester maternal serum samples. **f** studies likely to be from the same population. **g** quality score, high quality ≥ 28.4, low quality < 28.4. Abbreviations: ALL, acute lymphoblastic leukaemia; AN, antenatal; CI, confidence interval; CMV, cytomegalovirus; EBV, Epstein-Barr virus; ES, estimate; HBV, hepatitis B virus; HHV6, human herpes virus 6; IgM, Immunoglobulin M; med records, medical records; mycoplasma p., mycoplasma pneumoniae; NGS, next-generation sequencing on neonatal blood spot; PCR, polymerase chain reaction on neonatal blood spot; UTI, urinary tract infection; STI, sexually transmitted infection. Weights are from random effect analysis

Associations between any maternal infection during pregnancy and different cancer types are shown in Fig. 3 and Additional file 9: Fig. S2. Any maternal infection during pregnancy was associated with an increased risk of overall leukaemia (ES = 1.27; 95%CI, 1.01–1.60; *I*^*2*^ = 79.7%; *p* < 0.001), ALL (ES = 1.31; 95%CI, 1.04–1.64; *I*^2^ = 77.8%; *p* < 0.001) and solid tumours (ES = 1.32; 95%CI,1.05–1.66; *I*^2^ = 36.7%; *p* = 0.070) (Fig. 3). We observed publication bias in the analyses for leukaemia and ALL, with weaker pooled estimates after applying the trim and fill method (ES = 1.14; 95% CI, 0.87–1.49 and ES = 1.20; 95% CI, 0.89–1.62; Additional file 5: Fig. S1). Results from the leave-one-out sensitivity analysis indicated that omitting any single study did not substantially alter the pooled estimates, suggesting that the findings were robust (Additional file 6: Table. S5). However, we noted weaker associations when the analysis was limited to recently conducted studies (P_meta-regression_ = 0.009) and slightly stronger associations in cohort studies (P_meta-regression_ = 0.014) for any maternal infection and risk of ALL (Additional file 8: Table. S7).

**Fig. 4:**
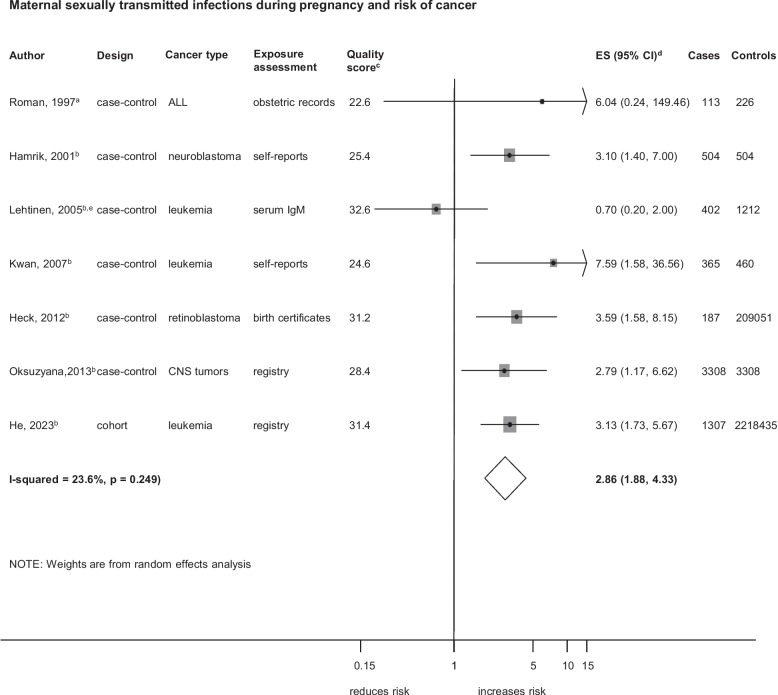
Maternal sexually transmitted infections (STIs) during pregnancy and the risk of overall childhood cancer STIs here include broad categorization of STIs (collectively including chlamydia, gonorrhoea, genital ulcers, genital herpes, human papillomavirus, vulvar warts) and specific STIs in the studies by Roman, 1997 [16] (vulvar warts), Lehtinen, 2005 [44] (chlamydia) and Oksuzyan, 2013 [49] (genital herpes). **a** crude odds ratio taking matching into account. **b** Adjusted estimate as indicated by published study. **c** Quality score, high quality ≥ 28.1, low quality < 28.1. **d** ES includes single-study odds ratios or hazard ratios and summary odds ratios. **e** first-trimester maternal serum samples. Abbreviations: ALL, acute lymphoblastic leukaemia; CNS, central nervous system; ES, estimate; CI, confidence interval; IgM, Immunoglobulin M. Weights are from random effect analysis

Associations between specific maternal infections during pregnancy and overall childhood cancer are shown in Fig. 4, Additional file 10: Fig. S3, Additional file 11: Fig. S4 and Additional file 12: Fig. S5. STIs were associated with an increased risk of overall childhood cancer (ES = 2.86; 95%CI, 1.88–4.33;* I*^*2*^ = 23.6%; *p* = 0.249; Fig. 4). Viral infection (ES = 1.43; 95% CI, 1.18–1.74; *I*^2^ = 52.3%; *p* < 0.001; Additional file 10: Fig. S3), cytomegalovirus (CMV) infection (ES = 2.10; 95%CI, 1.02–4.34; *I*^2^ = 67.1%; *p* = 0.010) and rubella infection (ES = 2.04; 95%CI, 1.11–3.75; *I*^2^ = 0%; *p* = 0.738; Additional file 11: Fig. S4) were associated with an increased risk of childhood cancer. GUTIs (ES = 1.52; 95%CI, 1.05–2.19; *I*^2^ = 69.3%; *p* = 0.001) were also associated with an increased risk of childhood cancer (Additional file 12: Fig. S5). We observed publication bias only for the analyses of viral infections and childhood cancer, with the estimate changing marginally after adjustment using the trim-and-fill method with three additional studies included (ES = 1.40; 95%CI, 1.14–1.71; Additional file 5: Fig. S1). Although study-level characteristics did not appear to influence these associations, our subgroup analysis assessing the CMV and risk of childhood cancer showed a stronger association in cohort studies. The leave-one-out sensitivity analysis indicated that the results were robust for most associations. However, the positive association between CMV infection and the risk of childhood cancer emerged only after excluding the studies by Holl et al., 2008 [40] and Lehtinen et al., 2003 [43], whereas the association for EBV infection became significant only when the study by Tedeschi et al., 2009 [56], was excluded (Additional file 6: Table. S5). When restricted to high-quality studies the association remained strongly positive for between STIs (n = 4; ES = 2.44; 95% CI, 1.36–4.38) and GUTIs (n = 1; ES = 2.42; 95%CI,1.50–3.91) and were weaker for viral infections (n = 7; ES = 1.35;95%CI,0.95–1.86) and CMV infection (n = 3; ES = 1.73;95%CI,0.68–4.39) (Additional file 7: Table. S6). All the studies in the analysis of rubella infection and childhood cancer were of low-quality (Additional file 7: Table. S6). We did not find an association between bacterial infections and childhood cancer risk.

**Fig. 5:**
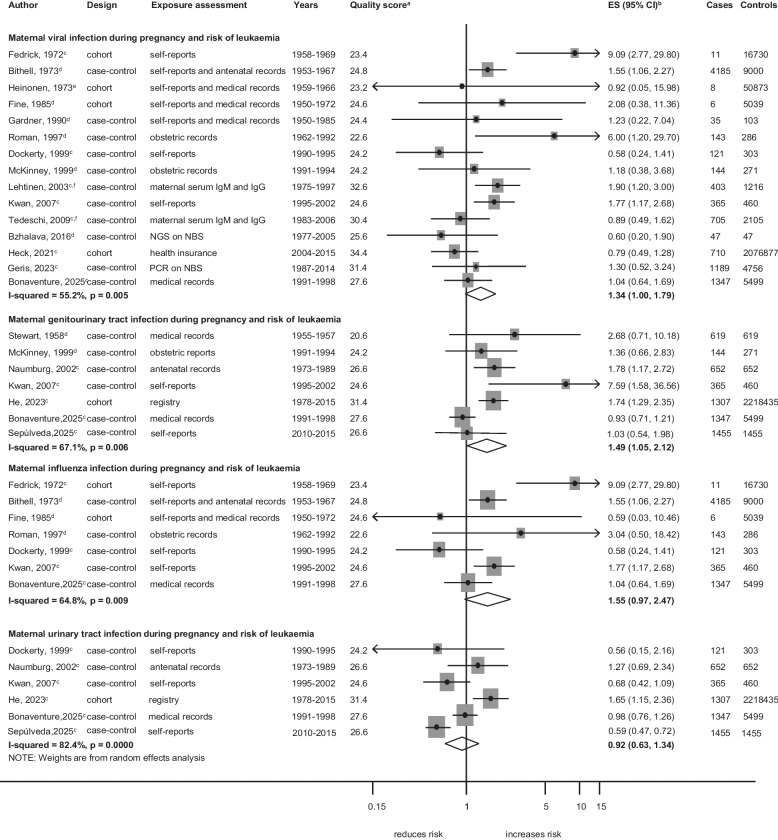
Specific maternal infections during pregnancy and the risk of leukaemia. **a** quality score, high quality ≥ 28.4, low quality < 28.4. **b** ES includes single-study odds ratios or hazard ratios and summary odds ratios. **c** Adjusted estimate as indicated by published study. **d** crude odds ratio taking matching into account. **e** no adjustment/matching. **f** first-trimester maternal serum samples. Abbreviations: ES, estimate; CI, confidence interval; IgM, Immunoglobulin M; NGS, next-generation sequencing; NBS, neonatal blood spot; PCR, polymerase chain reaction. Weights are from random effect analysis

**Fig. 6:**
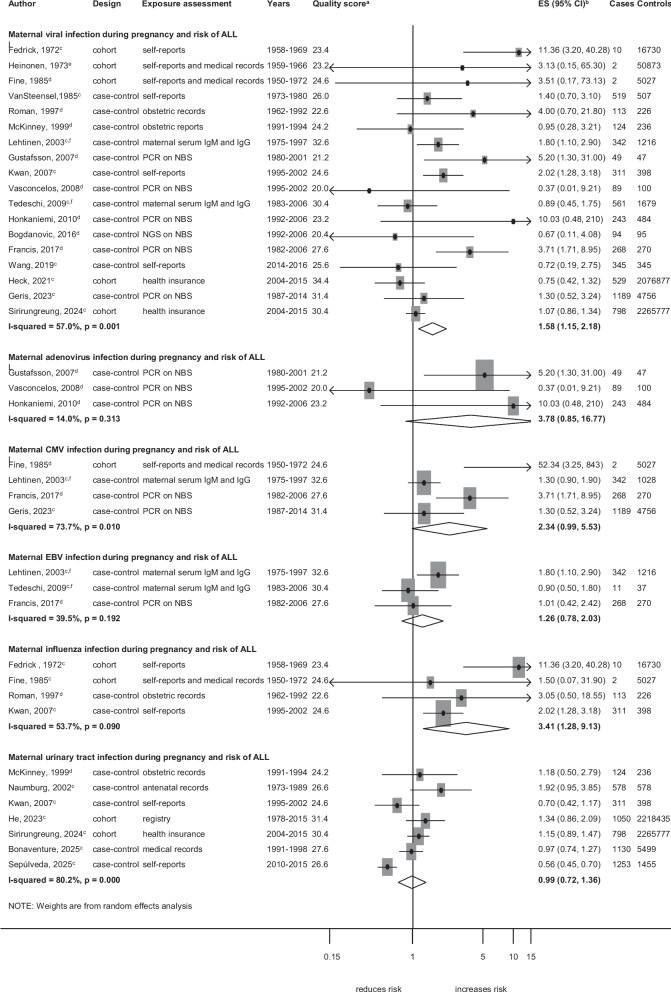
Specific maternal infections during pregnancy and the risk of acute lymphoblastic leukaemia (ALL). **a** quality score, high quality ≥ 28.4, low quality < 28.4. **b** ES includes single-study odds ratios or hazard ratios and summary odds ratios. **c** Adjusted estimate as indicated by published study. **d** crude odds ratio taking matching into account. **e** no adjustment/matching. **f** first-trimester maternal serum samples. Abbreviations: ALL, acute lymphoblastic leukaemia; CI, confidence interval; CMV, cytomegalovirus; IgM, Immunoglobulin M; EBV, Epstein Barr virus; ES, estimate; med records, medical records; NGS, next-generation sequencing on neonatal blood spot; PCR, polymerase chain reaction on neonatal blood spot. Weights are from random effect analysis

**Fig. 7:**
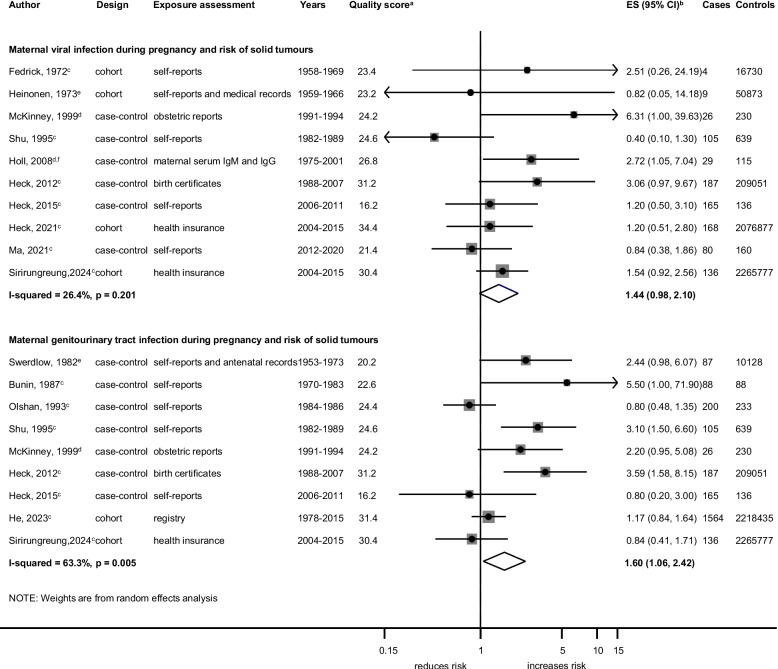
Specific maternal infections during pregnancy and the risk of solid tumours. Solid tumours include those broadly categorized as “solid tumours” in the studies and individual solid tumour types in specific studies: Wilms tumours, retinoblastomas, hepatoblastomas, malignant germ cell tumours, and testicular cancers. Viral infection types in this category include general viral infection and specific viral infections such as influenza, common cold, Epstein-Barr virus and genital herpes (**a**) quality score, high quality ≥ 28.4, low quality < 28.4. **b** ES includes single-study odds ratios or hazard ratios and summary odds ratios. **c** Adjusted estimate as indicated by published study. **d** crude odds ratio taking matching into account. **e** no adjustment/matching. **f** cancers except leukaemia, brain tumours and lymphomas. Abbreviations: ES, estimate; CI, confidence interval; IgM, Immunoglobulin M; IgG, Immunoglobulin G. Weights are from random effect analysis

Associations between specific maternal infections during pregnancy and specific cancer types are shown in Figs. 5, 6 and 7 and Additional file 13: Fig. S6 and Additional file 14: Fig. S7. GUTI was associated with an increased risk of leukaemia (ES = 1.49; 95%CI,1.05–2.12;* I*^2^ = 67.1%; *p* = 0.006; Fig. 5). Viral infection (ES = 1.58; 95%CI, 1.15–2.18; *I*^2^ = 57.0%;* p* = 0.001) and specifically influenza (ES = 3.41; 95%CI, 1.28–9.13; *I*^2^ = 53.7%;* p* = 0.090) were associated with an increased risk of ALL subtype (Fig. 6). GUTI was associated with an increased risk of solid tumours (ES = 1.60; 95%CI, 1.06–2.42; *I*^2^ = 63.3%;* p* = 0.005; Fig. 7). This association was weaker when the analysis was limited to cohort studies (P_meta-regression_ < 0.001) and to studies that adjusted for additional confounders (P_meta-regression_ = 0.006), whereas slightly stronger associations were observed for studies that used medical records as exposure assessment methods (P_meta-regression_ = 0.019). In the analyses assessing GUTIs and risk of leukaemia, we observed a weaker association for the subgroup of case–control studies and in those relying on self-reports for exposure assessment (Additional file 8: Table. S7). The leave-one-out sensitivity analysis revealed that the association between influenza infection and the risk of leukaemia became apparent only after the exclusion of one study by Dockerty et al. Similarly, an association between viral infection and the risk of solid tumours was observed when either the study by Shu et al. or Ma et al. was excluded (Additional file 6: Table. S5). Publication bias was not observed in these analyses. The detailed results for sensitivity analyses and subgroup analyses by study-level characteristics for each observed association are summarized in Additional file 6: Table. S5, Additional file 7: Table. S6 and Additional file 8: Table. S7.

## Discussion

Our meta-analysis demonstrates a positive association between maternal infections during pregnancy and the risk of cancer in the offspring overall, and particularly leukaemia and solid tumours. Notably, STIs, GUTIs and viral infections were associated with an increased cancer risk in the offspring. There are some biologically plausible mechanisms linking specific maternal infections to the development of certain childhood cancers. We discuss these potential links by examining the results according to both the type of infection and the type of cancer outcome.

### Sexually Transmitted Infections (STIs)

We found that STIs of the mother during pregnancy were associated with an increased risk of cancer in the offspring as supported by seven studies, four of which were high-quality studies: one cohort study using registry-confirmed infections [35] and three high-quality case–control studies, using birth certificates and registry data [35, 36, 49] for infection assessment and maternal bacterial antibodies [44] respectively. Majority of the studies examined STIs (including chlamydia, gonorrhoea, genital ulcers, genital herpes, human papilloma virus (HPV)) as a broad group of infections, except for three studies which considered chlamydia, genital herpes and vulvar warts separately [16, 44, 49]. The positive association persisted across all subgroup analyses based on study-level characteristics. In addition, we confirmed with the leave-one-out cross-validation method that the association was not driven by a single study, highlighting the robustness of our finding. These methodological considerations provide strong evidence supporting a positive association between maternal STIs during pregnancy and childhood cancer. Previous research has reported potential biological mechanisms linking STIs during pregnancy to adverse outcomes in offspring. For example, bacterial STIs from ascending infections such as chlamydia and gonorrhoea, and transplacentally transmitted infections like syphilis, can lead to perinatal complications such as preterm birth and low birth weight and congenital anomalies, which are risk factors for various childhood cancers [8, 67–69]. Persistent inflammation and cellular damage triggered by STIs may create a micro environment conducive to carcinogenesis in the developing foetus [70]. Similarly, viral STIs such as HPV also present a potential risk, although vertical transmission is relatively rare [71], HPV DNA has been detected in placenta, amniotic fluid and foetal membranes, implying in utero transmission [71, 72]. HPV’s ability to persist, activate and replicate in differentiated squamous epithelial cells [73] may explain associations observed with certain epithelial-origin childhood cancers, such as retinoblastomas [74] and CNS tumours. While we could not assess individual STIs due to lack of pathogen-specific data in most studies, the consistent associations observed in high-quality studies, together with the biological mechanisms discussed, strengthen the evidence for the association between STIs in pregnancy and cancer in the offspring. Nonetheless, we cannot rule out the possibility of residual confounding from unmeasured confounders such as maternal smoking, alcohol use, or other health behaviours that may be associated both with STI and childhood cancer.

### Genitourinary Tract Infections (GUTIs)

GUTIs during pregnancy were associated with an increased risk of overall childhood cancers, particularly leukaemia and solid tumours. For leukaemia, the analysis was based on seven studies with the positive association persisting across study-level characteristics including confounder adjustment and study quality. A strong positive association was found in the only high-quality study which was a cohort study design with record-based exposure assessment and adjustment for key confounders [35], with evidence of weaker associations in the remaining studies. This aligns with the previous meta-analysis by He [9], strengthening the evidence for the association between GUTIs and leukaemia.

For the association between GUTIs during pregnancy and risk of solid tumours, the analysis was based on nine studies of various tumour types (germ-cell tumours, hepatic tumours, hepatoblastomas, retinoblastomas, testicular cancers, and Wilms tumours) [24, 36, 37, 47, 50, 51, 55]. Due to the small number of individual solid tumour cases, we analysed these outcomes collectively. Our subgroup analysis showed a weaker association in studies with a cohort design and in those that reported estimates adjusted for additional confounders. The positive association was no longer observed in the more recently conducted studies. Further, the positive association with solid tumours was weaker in high-quality studies and was mainly driven by a single study on retinoblastomas [36], limiting its generalizability to other solid tumours and suggesting that GUTIs may specifically influence the risk of retinoblastomas. It is also plausible that there may be some confounding factors that may have led to this result, or the result could be a random observation. Nevertheless, GUTIs may be vertically transmitted across the maternal-foetal membranes and may cause an altered foetal immune response [8] or indirectly trigger a foetal inflammatory response through the infection metabolites, which can mediate epigenetic changes and subsequently increase cancer susceptibility [7].

### Viral infections

Our meta-analysis found that maternal viral infections during pregnancy were associated with an increased risk of childhood cancer overall and particularly with ALL. Specifically, CMV and rubella virus infection showed an elevated risk of overall childhood cancer, while influenza infection was positively associated with ALL, aligning with findings from He et al. [9]. Several biological mechanisms may underlie these associations. In the development of childhood leukaemia specifically, the current theory postulates a two-stage process: the first “stage” occurs in utero, where viral integration or inflammation induces initial genetic changes, and the second postnatally, with additional mutations or environmental factors driving leukaemia progression [75]. Viral infections, such as influenza and CMV, may cause damage and inflammation to the placenta and alter the microenvironment of the foetal bone marrow, promoting leukemogenesis. Although these mechanisms are biologically plausible in linking viral infections to childhood cancer, evidence from high-quality studies in our meta-analysis was weaker. Further, for influenza, the exposure assessment mainly relied on self-reported symptoms rather than laboratory-confirmed diagnoses, raising a possibility of misclassification as there are other flu-like illnesses such as CMV, which may partly explain the observed association between “influenza” and leukaemia risk. For rubella, the findings were from few studies of low-quality evidence, limiting their validity. Although no studies in our meta-analysis captured exposures during the COVID-19 pandemic, the potential for viral-modulated effects remains relevant. A recent 2025 study by Behnaz et al. [76] examined early exposure to COVID-19, combining the pre-pregnancy and pregnancy periods, and reported an increased risk of childhood cancer in offspring. Although this study was not included in our meta-analysis due to its combined exposure period, it highlights the need for further research on infections during the post-pandemic period to better understand the potential effects on childhood cancer risk.

### Bacterial vs. viral aetiology

In the present meta-analysis, we observed that maternal viral infections were more strongly associated with childhood cancer risk than bacterial infections. However, this may be influenced by the fact that evidence on bacterial infections is limited, with only four studies assessing their association compared to 36 studies on viral infections. Furthermore, since most studies assessing STIs and GUTIs included in the meta-analysis did not specify the causative pathogens, we could not confirm this assumption. Nevertheless, we acknowledge the need to further explore the biological mechanisms linking bacterial infections to childhood cancers. While viral infections have been more extensively studied in relation to oncogenesis, bacterial infections may also play a role through distinct mechanisms. For instance, Escherichia coli (E.coli) is the most common cause of GUTIs, and several STIs including chlamydia, gonorrhoea, and syphilis, are of bacterial origin. Although the evidence on bacterial infections and childhood cancer risk remains limited, the association is biologically plausible. Bacterial infections during pregnancy may ascend the genital tract or cross the placenta, triggering inflammatory responses. Such inflammation could lead to epigenetic modifications or adverse birth outcomes like low birth weight and prematurity, both of which have been linked to increased cancer risk in the offspring. A clearer distinction between bacterial and viral mechanisms is needed in future research to better understand their respective roles in early-life cancer development.

### Medication use and inflammatory pathways

Beyond all the aforementioned mechanisms linking maternal infections during pregnancy to cancer in the offspring, medications that act through inflammatory pathways, such as fever-reducing analgesics, could influence the development of childhood cancers as observed in our meta-analysis between medications during pregnancy and childhood cancer risk [77].

### Strengths and limitations

The strengths of our analysis include the assessment of diverse infections and cancer outcomes that have not been comprehensively studied before, providing a broader understanding of maternal infections in pregnancy and risk of childhood cancer. We set a minimum threshold of three studies per analysis, which potentially improved the statistical power and reduced potential outlier effects. Our self-developed quality assessment tool allowed for a thorough appraisal of the studies with methodology and risk of bias considerations. We conducted extensive sensitivity and subgroup analyses to assess heterogeneity, which improved the interpretability and robustness of our results.

However, our systematic review and meta-analysis has several limitations that should be considered when interpreting the findings. Firstly, most studies in this meta-analysis relied on maternal self-reported infections during pregnancy, which may be prone to recall bias. While such misclassification is often assumed to be non-differential, the predominance of case–control designs in the included studies raises the possibility of differential misclassification, as mothers of affected children may recall exposures differently from controls. As a result, the direction of bias cannot be assumed, consistent with recent methodological discussions [78]. Secondly, the inadequate adjustment of key confounders such as perinatal factors, other infections during pregnancy, maternal health behaviours (including smoking or alcohol consumption) and comorbidities may have complicated isolating the effects of maternal infections during pregnancy on cancer in the offspring. Thirdly, the general lack of precise timing regarding gestational age at infection limits our ability to identify potential windows of vulnerability, which could inform prevention strategies. Furthermore, most of the included studies did not distinguish between acute and chronic infections or the severity of the infections, which hindered deeper insights into the mechanisms of the association with childhood cancer and the risk categorisation. Our observation of publication bias in the analysis for overall maternal infections and overall cancer risk, leukaemia and ALL, and for viral infections and overall cancer risk may have led to an overestimation of the true association and a distorted conclusion, as studies with significant findings are more likely to be published than those with null results. However, the trim-and-fill method yielded only modestly attenuated pooled estimates, indicating that the association between maternal infections during pregnancy and childhood cancer risk remained largely consistent. Lastly, our attempt to explore general potential mechanisms by pooling diverse infection types and cancer outcomes may have resulted in biased estimates. However, the overall association remains biologically plausible given the role of inflammation in carcinogenesis.

## Conclusions

In conclusion, our systematic review and meta-analysis indicates that maternal infections during pregnancy are associated with an increased risk of childhood cancer. Specifically, STIs, GUTIs, and viral infections showed increased risk of overall childhood cancer, with GUTIs and viral infections also linked to higher risk of leukaemias. In light of these findings, primary prevention of pregnancy infections may be a valuable strategy to reduce potential cancer risk in offspring. Promoting good health and hygiene practices, as well as vaccinations against vaccine-preventable diseases such as influenza during pregnancy, could offer benefits beyond immediate maternal and foetal health. For STIs and GUTIs, routine screening and timely treatment, where available, may help prevent adverse perinatal outcomes and, potentially, long-term health risks, including cancer in children. As research on the subject of maternal infections and cancer risk is still currently underdeveloped, there is a need for large-scale prospective studies stratified by pathogen type as well as mechanistic studies to advance knowledge in this area. Future research should provide a comprehensive exposure assessment that considers the timing, chronicity, severity, and therapeutic interventions for maternal infections during pregnancy, as these factors may yield valuable insights.

## Supplementary Information

Additional file 1: Table. S1: PRISMA checklist.

Additional file 2: Table. S2: Search strategy.

Additional file 3: Table. S3: Extracted data and estimates for the meta-analysis Abbreviations: AML, acute myeloid leukaemia; ALL; acute lymphoblastic leukaemia; CMV, cytomegalovirus; CNS, central nervous system; EBV, Epstein Barr virus; ES estimate; IgG, immunoglobulin G; IgM, immunoglobulin M; LCI, lower confidence interval; NHL, non-Hodgkin lymphoma; TTV, torque teno virus; TTMV, torque teno mini virus ; TTMDV, torque teno midi virus; UCI, upper confidence interval; UTI, urinary tract infection.

Additional file 4: Table. S4: Studies excluded from systematic review and meta-analysis.

Additional file 5: Fig.S1: Publication bias for maternal infection during pregnancy and risk of cancer. Trim and fill analyses were conducted for meta-analyses with evidence of publication bias. Abbreviations: CI, confidence interval; Conf, confidence; Eff, effect; Err, error; logor, log odds ratio; MSE, mean square error; P, P-value; REML, restricted maximum likelihood; se; standard error; SND, standard normal deviate; Std, standard.

Additional file 6: Table. S5: Sensitivity analysis – leave-one-out meta-analysis.

Additional file 7: Table. S6: Summary of meta-analyses showing positive associations. Heterogeneity was examined with the I² statistic, categorized as low (≤50%), moderate (>50-75%), or substantial (>75%), with statistical significance assessed using the Q statistic (P-value for heterogeneity).Study quality (maximal score = 45) was stratified above the 4th quintile for quality score (≥28.4 = high quality) and below (<28.4 = low quality). Publication bias was assessed using Egger tests and funnel plots for analyses which had at least 5 studies. Abbreviations. ALL, acute lymphoblastic leukaemia; CI, confidence interval; CMV cytomegalovirus.

Additional file 8: Table. S7: Subgroup analyses of observed associations by study-level characteristics. *P values for the subgroups are calculated using meta-regression, some P values not possible/not done due to few available studies for meta-regression, and some omitted due to collinearity. For exposure assessment, if studies report more than one method of assessment the most valid exposure assessment method is chosen. For outcome assessment, no registry means outcome assessed using methods other than registry or pathological confirmation. For confounder adjustment, basic means matching, or adjustment for basic characteristics e.g., age, sex, region; other factors include adjustments for comorbidities, genetic disorders, proxies for infection exposure such as sibship size, mode of delivery; similar factors include adjustments for other infections and antibiotics. Quality, high ≥ 28.4, cut off and low < 28.4 from the quality assessment tool. Abbreviations: ES estimate; UCI, upper confidence interval; LCI, lower confidence interval; Ref, reference value for subgroup analyses involving three categories.

Additional file 9: Fig.S2: Maternal exposure to any infection during pregnancy and the risk of CNS tumours and neuroblastomas and lymphoma. Lymphoma includes all subtypes. a) crude odds ratio taking matching into account. (b) Adjusted estimate as indicated by published study. (c) no adjustment/matching. (d) ES includes single-study odds ratios or hazard ratios and summary odds ratios. (e) first-trimester maternal serum samples. (f) quality score, high quality ≥28.4, low quality <28.4. Abbreviations: CNS, central nervous system; CI, confidence interval; ES, estimate; IgM, immunoglobulin M; JCV, human polyomavirus 2; SV 40, simian virus 40. med records, medical records. Weights are from random effect analysis.

Additional file 10: Fig.S3: Maternal Infection by type of pathogen – viral and bacterial, and overall childhood cancer risk. Abbreviations: ES, estimate; CI confidence interval.

Additional file 11: Fig.S4: Specific viral infections and overall childhood cancer risk. Abbreviations: CMV, cytomegalovirus; EBV, epstein-barr virus; ES, estimate. CI, confidence interval.

Additional file 12: Fig.S5: Maternal Infection by affected body system and overall childhood cancer risk. Abbreviations: ES, estimate; CI, confidence interval.

Additional file 13: Fig.S6: Specific maternal infections during pregnancy and the risk of central nervous system tumours and neuroblastomas. (a) quality score, high quality ≥28.4, low quality <28.4. (b) ES includes single-study odds ratios or hazard ratios and summary odds ratios. (c) Adjusted estimate as indicated by published study. (d) crude odds ratio taking matching into account. (e) no adjustment/matching. (f) first-trimester maternal serum samples. Abbreviations: CNS, central nervous system; ES, estimate; CI, confidence interval; IgM, Immunoglobulin M; IgG, Immunoglobulin G. Weights are from random effect analysis.

Additional file 14: Fig.S7: Maternal viral infection during pregnancy and the risk of lymphoma. Lymphoma includes all subtypes. (a) quality score, high quality ≥28.4, low quality <28.4. (b) ES includes single-study odds ratios or hazard ratios and summary odds ratios. (c) Adjusted estimate as indicated by published study. (d) crude odds ratio taking matching into account. Abbreviations: ES, estimate; CI, confidence interval. Weights are from random effect analysis.

## Data Availability

The datasets used and/or analysed during the current study are available from the corresponding author on reasonable request.

## Abbreviations

ALL: Acute lymphoblastic leukaemias
CI: Confidence interval
CMV: Cytomegalovirus
CNS: Central nervous system
DNA: Deoxyribonucleic acid
EBV: Epstein-barr virus
ES: Estimate
GUTI: Genitourinary tract infection
HBV: Hepatitis B virus
HCV: Hepatitis C virus
HPV: Human papillomavirus
HR: Hazard ratio
IgM: Immunoglobulin M
NOS: Newcastle-ottawa scale
OR: Odds ratio
ROBINS-E: Risk of bias in non-randomized studies of exposure
ROBINS-I: Risk of bias in non-randomized studies of interventions
RR: Relative risk
SD: Standard deviation
SE: Standard error
STI: Sexually transmitted infection
