# Supplementary material for: Maternal infection during pregnancy and the risk of childhood cancer: a systematic review and meta-analysis
Source: BMC Med. 2026 Jan 14;24:51. doi: 10.1186/s12916-026-04625-1 (PMC12849171; doi:10.1186/s12916-026-04625-1)
Supplement: Supplementary file 2 — Additional file 2: Table. S2: Search strategy. [file 12916_2026_4625_MOESM2_ESM.docx]

**Additional file 2: Table. S2: Search Strategy -Search Terms in PubMed, Web of Science, Embase and Cochrane Library**

|  | **Search Terms** |
| --- | --- |
| **PubMed (First searched in April 2024, updated April 2025 and 13.10.2025)** |  |
| #1 | "Bacterial Infections and Mycoses"[MeSH Terms] OR "Virus Diseases"[MeSH Terms] OR "Parasitic Diseases"[MeSH Terms] OR "infect*"[Title/Abstract] OR "bacteri*"[Title/Abstract] OR "virus"[Title/Abstract] OR "viral"[Title/Abstract] OR "fungus"[Title/Abstract] OR "fungi"[Title/Abstract] OR "fungal"[Title/Abstract] OR "parasite*"[Title/Abstract] |
| #2 | "Maternal Exposure"[MeSH Terms] OR "maternal"[Title/Abstract] OR "intrauterine"[Title/Abstract] OR "in utero"[Title/Abstract] OR "prenatal"[Title/Abstract] OR "antenatal"[Title/Abstract] OR "Pregnancy"[MeSH Terms] OR "Pregnant people"[MeSH Terms] OR "Pregnancy Trimesters"[MeSH Terms] OR "pregnan*"[Title/Abstract] OR "gestation*"[Title/Abstract] OR "conception*"[Title/Abstract] OR "trimester*"[Title/Abstract] |
| #3 | "Fetus"[MeSH Terms] OR "fetus*"[Title/Abstract] OR "foetus*"[Title/Abstract] OR "foetal*"[Title/Abstract] OR "fetal*"[Title/Abstract] OR "Fetal Blood"[MeSH Terms] |
| #4 | "Dried Blood Spot Testing"[MeSH Terms] OR "cord blood*"[Title/Abstract] OR "guthrie card*"[Title/Abstract] OR "blood spot*"[Title/Abstract] |
| #5 | #2 OR #3 OR #4 |
| #6 | #1 AND #5 |
| #7 | "Neoplasms"[MeSH Terms] |
| #8 | "Infant"[MeSH Terms] OR "Child"[MeSH Terms] OR "Adolescent"[MeSH Terms] OR "newborn*"[Title/Abstract] OR "neonat*"[Title/Abstract] OR "infan*"[Title/Abstract] OR "toddler*"[Title/Abstract] OR "child*"[Title/Abstract] OR "adolescen*"[Title/Abstract] OR "juvenile"[Title/Abstract] OR "teen*"[Title/Abstract] OR "girl*"[Title/Abstract] OR "boy"[Title/Abstract] OR "youth*"[Title/Abstract] OR "paediatric*"[Title/Abstract] OR "pediatric*"[Title/Abstract] |
| #9 | #7 AND #8 |
| #10 | #6 AND #9 |
| **Web of Science**  **(search date 13.10.2025)** |  |
| #1 | TS= (infection or (infect* or bacteri* or virus or viral or fungus or fungi or fungal or parasite*)) |
| #2 | TS=((maternal exposure) OR (maternal)) |
| #3 | TS= ((prenatal exposure) OR (prenatal period) OR (intrauterine) OR (in utero) or (prenatal) or (antenatal)) |
| #4 | (((((TS=(pregnancy)) OR TS= (pregnant woman)) OR TS=(pregnan*)) OR TS=(gestation*)) OR TS=(conception*)) OR TS=(trimester*) |
| #5 | (TS=(fetus)) OR TS=(fetus* OR foetus* OR foetal* or fetal*) |
| #6 | TS="Dried Blood Spot Testing" OR (TS="cord blood") OR (TS="guthrie card*") OR (TS="blood spot*") |
| #7 | #2 OR #3 OR #4 OR #5 OR #6 |
| #8 | #1 AND #7 |
| #9 | (TS=(cancer)) OR TS=(neoplasm) |
| #10 | TS=(newborn* or neonat* or infan* or toddler* or child* or adolescen* or juvenile or teen* or girl* OR boy* or youth* or paediatric* or pediatric*) |
| #11 | #9 AND #10 |
| #12 | #8 AND #11 |
| **Embase (searched on 13.10.2025)** |  |
| #1 | infection/exp OR 'virus infection'/exp OR parasitosis/exp OR infect*:ti,ab OR bacteri*:ti,ab OR virus:ti,ab OR viral:ti,ab OR fungus:ti,ab OR fungi:ti,ab OR fungal:ti,ab OR parasite*:ti,ab |
| #2 | 'Maternal Exposure'/exp OR maternal:ti,ab OR intrauterine:ti,ab OR 'in utero':ti,ab OR prenatal:ti,ab OR antenatal:ti,ab OR Pregnancy/exp OR 'Pregnant Woman'/exp OR pregnan*:ti,ab OR gestation*:ti,ab OR conception*:ti,ab OR trimester*:ti,ab |
| #3 | Fetus/exp OR fetus*:ti,ab OR foetus*:ti,ab OR foetal*:ti,ab OR fetal*:ti,ab OR 'Fetus Blood'/exp |
| #4 | 'Dried Blood Spot Testing'/exp OR 'cord blood*':ti,ab OR 'guthrie card*':ti,ab OR 'blood spot*':ti,ab |
| #5 | #2 OR #3 OR #4 |
| #6 | #1 AND #5 |
| #7 | Neoplasm/exp |
| #8 | Infant/exp OR Child/exp OR Adolescent/exp OR newborn*:ti,ab OR neonat*:ti,ab OR infan*:ti,ab OR toddler*:ti,ab OR child*:ti,ab OR adolescen*:ti,ab OR juvenile:ti,ab OR teen*:ti,ab OR girl*:ti,ab OR boy:ti,ab OR youth*:ti,ab OR paediatric*:ti,ab OR pediatric*:ti,ab |
| #9 | #7 AND #8 |
| #10 | #6 AND #9 |
| #11 | #10 AND [embase]/lim NOT ([embase]/lim AND [medline]/lim) |
| **Cochrane Library (searched on 13.10.2025)** |  |
| #1 | [mh "bacterial infections and mycoses"] OR [mh "virus diseases"] OR [mh "parasitic diseases"] OR infect*:ti,ab OR bacteri*:ti,ab OR virus:ti,ab OR viral:ti,ab OR fungus:ti,ab OR fungi:ti,ab OR fungal:ti,ab OR parasite*:ti,ab |
| #2 | [mh "Maternal Exposure"] OR maternal:ti,ab OR intrauterine:ti,ab OR "in utero":ti,ab OR prenatal:ti,ab OR antenatal:ti,ab OR [mh Pregnancy] OR [mh "Pregnant people"] OR [mh "Pregnancy Trimesters"] OR pregnan*:ti,ab OR gestation*:ti,ab OR conception*:ti,ab OR trimester*:ti,ab |
| #3 | [mh Fetus] OR fetus*:ti,ab OR foetus*:ti,ab OR foetal*:ti,ab OR fetal*:ti,ab OR [mh "Fetal Blood"] |
| #4 | [mh "Dried Blood Spot Testing"] OR ("cord" NEXT blood*):ti,ab OR ("guthrie" NEXT card*):ti,ab OR ("blood" NEXT spot*):ti,ab |
| #5 | #2 OR #3 OR #4 |
| #6 | #1 AND #5 |
| #7 | [mh neoplasms] |
| #8 | [mh Infant] OR [mh Child] OR [mh Adolescent] OR newborn*:ti,ab OR neonat*:ti,ab OR infan*:ti,ab OR toddler*:ti,ab OR child*:ti,ab OR adolescen*:ti,ab OR juvenile:ti,ab OR teen*:ti,ab OR girl*:ti,ab OR boy:ti,ab OR youth*:ti,ab OR paediatric*:ti,ab OR pediatric*:ti,ab |
| #9 | #7 AND #8 |
| #10 | #6 AND #9 |
