# Supplementary material for: Maternal infection during pregnancy and the risk of childhood cancer: a systematic review and meta-analysis
Source: BMC Med. 2026 Jan 14;24:51. doi: 10.1186/s12916-026-04625-1 (PMC12849171; doi:10.1186/s12916-026-04625-1)
Supplement: Supplementary file 3 — Additional file 3: Table. S3: Extracted data and estimates for the meta-analysis Abbreviations: AML, acute myeloid leukaemia; ALL; acute lymphoblastic leukaemia; CMV, cytomegalovirus; CNS, central nervous system; EBV, Epstein Barr virus; ES estimate; IgG, immunoglobulin G; IgM, immunoglobulin M; LCI, lower confidence interval; NHL, non-Hodgkin lymphoma; TTV, torque teno virus; TTMV, torque teno mini virus ; TTMDV, torque teno midi virus; UCI, upper confidence interval; UTI, urinary tract infection. [file 12916_2026_4625_MOESM3_ESM.docx]

| **Additional file 3:Table. S3: Extracted data and estimates used in meta-analysis** | | | | | | | | | | |  |  |  |
| --- | --- | --- | --- | --- | --- | --- | --- | --- | --- | --- | --- | --- | --- |
| **First author** | **Year** | **Title** | **Study design** | | **Type of infection** | | **Cancer outcome** | | **Cases** | **Controls** | **ES** | **LCI** | **UCI** |
| Birch | 1990 | The inter-regional epidemiological study of childhood  cancer (IRESCC)*: case-control study of children with  central nervous system tumours | Case-control | | chicken pox | | CNS tumours | | 78 | 156 | 2.01 | 0.12 | 32.6 |
| Birch | 1990 | The inter-regional epidemiological study of childhood  cancer (IRESCC)*: case-control study of children with  central nervous system tumours | Case-control | | measles | | CNS tumours | | 78 | 156 | 0.66 | 0.03 | 16.4 |
| Bithell | 1973 | Association between malignant disease in children and maternal virus infections during pregnancy | population-based case control | | influenza | | all cancers | | 9000 | 9000 | 1.52 | 1.11 | 2.14 |
| Bithell | 1973 | Association between malignant disease in children and maternal virus infections during pregnancy | population-based case control | | rubella | | all cancers | | 9000 | 9000 | 2.43 | 0.96 | 6.9 |
| Bithell | 1973 | Association between malignant disease in children and maternal virus infections during pregnancy | population-based case control | | varicella | | all cancers | | 9000 | 9000 | 15.01 | 0.86 | 262.89 |
| Bithell | 1973 | Association between malignant disease in children and maternal virus infections during pregnancy | population-based case control | | Varicella | | leukaemia | | 4185 | 9000 | 15.06 | 0.78 | 291.71 |
| Bithell | 1973 | Association between malignant disease in children and maternal virus infections during pregnancy | population-based case control | | influenza | | leukaemia | | 4185 | 9000 | 1.55 | 1.06 | 2.27 |
| Bithell | 1973 | Association between malignant disease in children and maternal virus infections during pregnancy | population-based case control | | other viral infections (mumps, infectious hepatitis, herpes zoster) | | leukaemia | | 4185 | 9000 | 0.69 | 0.33 | 1.41 |
| Bithell | 1973 | Association between malignant disease in children and maternal virus infections during pregnancy | population-based case control | | Rubella | | other lymphatic tissue disease | | 729 | 9000 | 5.31 | 1.37 | 20.57 |
| Bithell | 1973 | Association between malignant disease in children and maternal virus infections during pregnancy | population-based case control | | influenza | | other lymphatic tissue disease | | 729 | 9000 | 0.96 | 0.39 | 2.4 |
| Bithell | 1973 | Association between malignant disease in children and maternal virus infections during pregnancy | population-based case control | | other viral infections (mumps, infectious hepatitis, herpes zoster) | | other lymphatic tissue disease | | 729 | 9000 | 1.2 | 0.36 | 3.92 |
| Bithell | 1973 | Association between malignant disease in children and maternal virus infections during pregnancy | population-based case control | | Rubella | | other tumours | | 4086 | 9000 | 1.57 | 0.5 | 4.96 |
| Bithell | 1973 | Association between malignant disease in children and maternal virus infections during pregnancy | population-based case control | | Varicella | | other tumours | | 4086 | 9000 | 19.84 | 1.07 | 368.64 |
| Bithell | 1973 | Association between malignant disease in children and maternal virus infections during pregnancy | population-based case control | | influenza | | other tumours | | 4086 | 9000 | 1.55 | 1.06 | 2.28 |
| Bithell | 1973 | Association between malignant disease in children and maternal virus infections during pregnancy | population-based case control | | other viral infections (mumps, infectious hepatitis, herpes zoster) | | other tumours | | 4086 | 9000 | 0.92 | 0.48 | 1.77 |
| Blot | 1980 | Childhood cancer in relation to prenatal exposure to chickenpox | population-based case control | | chicken pox | | childhood cancer | | 2823 | 2484 | 0.8 | 0.3 | 2.1 |
| Blot | 1980 | Childhood cancer in relation to prenatal exposure to chickenpox | population-based case control | | herpes zoster | | childhood cancer | | 2823 | 2484 | 2.64 | 0.28 | 25.4 |
| Blot | 1980 | Childhood cancer in relation to prenatal exposure to chickenpox | population-based case control | | Rubella | | childhood cancer | | 2823 | 2484 | 2 | 0.9 | 4.6 |
| Bogdanovic | 2016 | Virome characterisation from Guthrie cards in children who later developed acute lymphoblastic leukaemia | Case-Control | | Human herpes virus 6 | | ALL | | 94 | 95 | 0.67 | 0.11 | 4.08 |
| Bogdanovic | 2016 | Virome characterisation from Guthrie cards in children who later developed acute lymphoblastic leukaemia | Case-Control | | parvovirus B 19 | | ALL | | 94 | 95 |  |  |  |
| Bonaventure | 2025 | Maternal illnesses during pregnancy and the risk of childhood cancer: A medical-record based analysis (UKCCS) | Case-Control | | any infection | | leukaemia | | 1347 | 5499 | 0.95 | 0.83 | 1.09 |
| Bonaventure | 2025 | Maternal illnesses during pregnancy and the risk of childhood cancer: A medical-record based analysis (UKCCS) | Case-Control | | any infection | | ALL | | 1130 | 5499 | 0.94 | 0.81 | 1.09 |
| Bonaventure | 2025 | Maternal illnesses during pregnancy and the risk of childhood cancer: A medical-record based analysis (UKCCS) | Case-Control | | any infection | | CNS tumours | | 510 | 5499 | 1.06 | 0.85 | 1.31 |
| Bonaventure | 2025 | Maternal illnesses during pregnancy and the risk of childhood cancer: A medical-record based analysis (UKCCS) | Case-Control | | any infection | | nephroblastoma | | 137 | 5499 | 1.07 | 0.72 | 1.59 |
| Bonaventure | 2025 | Maternal illnesses during pregnancy and the risk of childhood cancer: A medical-record based analysis (UKCCS) | Case-control | | any infection | | lymphoma | | 254 | 5499 | 1.37 | 1.02 | 1.85 |
| Bonaventure | 2025 | Maternal illnesses during pregnancy and the risk of childhood cancer: A medical-record based analysis (UKCCS) | Case control | | influenza | | leukaemia | | 1347 | 5499 | 1.04 | 0.64 | 1.69 |
| Bonaventure | 2025 | Maternal illnesses during pregnancy and the risk of childhood cancer: A medical-record based analysis (UKCCS) | Case control | | genital infection | | leukaemia | | 1347 | 5499 | 0.93 | 0.71 | 1.21 |
| Bonaventure | 2025 | Maternal illnesses during pregnancy and the risk of childhood cancer: A medical-record based analysis (UKCCS) | Case control | | UTI | | leukaemia | | 1347 | 5499 | 0.98 | 0.76 | 1.26 |
| Bonaventure | 2025 | Maternal illnesses during pregnancy and the risk of childhood cancer: A medical-record based analysis (UKCCS) | Case control | | UTI | | ALL | | 1130 | 5499 | 0.97 | 0.74 | 1.27 |
| Bonaventure | 2025 | Maternal illnesses during pregnancy and the risk of childhood cancer: A medical-record based analysis (UKCCS) | Case control | | UTI | | CNS tumours | | 510 | 5499 | 1.06 | 0.72 | 1.56 |
| Bonaventure | 2025 | Maternal illnesses during pregnancy and the risk of childhood cancer: A medical-record based analysis (UKCCS) | Case control | | chicken pox | | leukaemia | | 1347 | 5499 | 0.84 | 0.34 | 2.1 |
| Bunin | 1987 | Gestational risk factors for Wilms' tumour: results of a case-control study | population-based Case-control | | vaginal infection | | Wilm's Tumour | | 88 | 88 | 5.5 | 1 | 71.9 |
| Bunin | 1987 | Gestational risk factors for Wilms' tumour: results of a case-control study | population-based Case-control | | vaginal infection | | Wilm's Tumour | | 88 | 88 | 6 | 0.6 | infinity |
| Bzhalava | 2016 | Viremia during pregnancy and risk of childhood leukaemias and lymphomas in the offspring: Nested case-control study | Nested-Case Control | | TTV1 | | leukaemias | | 47 | 47 | 0.3 | 0.1 | 1.1 |
| Bzhalava | 2016 | Viremia during pregnancy and risk of childhood leukaemias and lymphomas in the offspring: Nested case-control study | Nested-Case Control | | TTV2 | | leukaemias | | 47 | 47 |  |  |  |
| Bzhalava | 2016 | Viremia during pregnancy and risk of childhood leukaemias and lymphomas in the offspring: Nested case-control study | Nested-Case Control | | TTV3 | | leukaemias | | 47 | 47 | 0.6 | 0.2 | 1.6 |
| Bzhalava | 2016 | Viremia during pregnancy and risk of childhood leukaemia and lymphomas in the offspring: Nested case-control study | Nested-Case Control | | TTV5 | | leukaemias | | 47 | 47 | 0.4 | 0.1 | 1.4 |
| Bzhalava | 2016 | Viremia during pregnancy and risk of childhood leukaemia and lymphomas in the offspring: Nested case-control study | Nested-Case Control | | TTV7 | | leukaemia | | 47 | 47 | 0.3 | 0 | 3.2 |
| Bzhalava | 2016 | Viremia during pregnancy and risk of childhood leukaemia and lymphomas in the offspring: Nested case-control study | Nested-Case Control | | TTV8 | | leukaemia | | 47 | 47 | 0.4 | 0.1 | 1.1 |
| Bzhalava | 2016 | Viremia during pregnancy and risk of childhood leukaemia and lymphomas in the offspring: Nested case-control study | Nested-Case Control | | TTV9 | | leukaemia | | 47 | 47 |  |  |  |
| Bzhalava | 2016 | Viremia during pregnancy and risk of childhood leukaemia and lymphomas in the offspring: Nested case-control study | Nested-Case Control | | TTV10 | | leukaemia | | 47 | 47 | 0.6 | 0.1 | 2.5 |
| Bzhalava | 2016 | Viremia during pregnancy and risk of childhood leukaemia and lymphomas in the offspring: Nested case-control study | Nested-Case Control | | TTV12 | | leukaemia | | 47 | 47 | 0.1 | 0 | 1.1 |
| Bzhalava | 2016 | Viremia during pregnancy and risk of childhood leukaemia and lymphomas in the offspring: Nested case-control study | Nested-Case Control | | TTV13 | | leukaemia | | 47 | 47 | 0.5 | 0.1 | 2 |
| Bzhalava | 2016 | Viremia during pregnancy and risk of childhood leukaemia and lymphomas in the offspring: Nested case-control study | Nested-Case Control | | TTV14 | | leukaemia | | 47 | 47 | 0.5 | 0.1 | 2 |
| Bzhalava | 2016 | Viremia during pregnancy and risk of childhood leukaemia and lymphomas in the offspring: Nested case-control study | Nested-Case Control | | TTV15 | | leukaemia | | 47 | 47 | 0.7 | 0.3 | 1.6 |
| Bzhalava | 2016 | Viremia during pregnancy and risk of childhood leukaemia and lymphomas in the offspring: Nested case-control study | Nested-Case Control | | TTV16 | | leukaemia | | 47 | 47 | 1 | 0.3 | 3.4 |
| Bzhalava | 2016 | Viremia during pregnancy and risk of childhood leukaemia and lymphomas in the offspring: Nested case-control study | Nested-Case Control | | TTV19 | | leukaemia | | 47 | 47 | 0.5 | 0.2 | 1.1 |
| Bzhalava | 2016 | Viremia during pregnancy and risk of childhood leukaemia and lymphomas in the offspring: Nested case-control study | Nested-Case Control | | TTV20 | | leukaemia | | 47 | 47 | 1 | 0.2 | 5.2 |
| Bzhalava | 2016 | Viremia during pregnancy and risk of childhood leukaemia and lymphomas in the offspring: Nested case-control study | Nested-Case Control | | TTV21 | | leukaemia | | 47 | 47 | 0.2 | 0 | 1.6 |
| Bzhalava | 2016 | Viremia during pregnancy and risk of childhood leukaemia and lymphomas in the offspring: Nested case-control study | Nested-Case Control | | TTV23 | | leukaemia | | 47 | 47 | 0.1 | 0 | 1.1 |
| Bzhalava | 2016 | Viremia during pregnancy and risk of childhood leukaemia and lymphomas in the offspring: Nested case-control study | Nested-Case Control | | TTV24 | | leukaemia | | 47 | 47 | 0.5 | 0.2 | 1.6 |
| Bzhalava | 2016 | Viremia during pregnancy and risk of childhood leukaemia and lymphomas in the offspring: Nested case-control study | Nested-Case Control | | TTV27 | | leukaemia | | 47 | 47 |  |  |  |
| Bzhalava | 2016 | Viremia during pregnancy and risk of childhood leukaemia and lymphomas in the offspring: Nested case-control study | Nested-Case Control | | TTV28 | | leukaemia | | 47 | 47 |  |  |  |
| Bzhalava | 2016 | Viremia during pregnancy and risk of childhood leukaemia and lymphomas in the offspring: Nested case-control study | Nested-Case Control | | TTV29 | | leukaemia | | 47 | 47 | 0.7 | 0.2 | 3.5 |
| Bzhalava | 2016 | Viremia during pregnancy and risk of childhood leukaemia and lymphomas in the offspring: Nested case-control study | Nested-Case Control | | Alphatorquevirus total | | leukaemia | | 47 | 47 | 0.6 | 0.2 | 1.5 |
| Bzhalava | 2016 | Viremia during pregnancy and risk of childhood leukaemia and lymphomas in the offspring: Nested case-control study | Nested-Case Control | | TTMV1 | | leukaemia | | 47 | 47 | 0.6 | 0.2 | 2.4 |
| Bzhalava | 2016 | Viremia during pregnancy and risk of childhood leukaemia and lymphomas in the offspring: Nested case-control study | Nested-Case Control | | TTMV2 | | leukaemia | | 47 | 47 |  |  |  |
| Bzhalava | 2016 | Viremia during pregnancy and risk of childhood leukaemia and lymphomas in the offspring: Nested case-control study | Nested-Case Control | | TTMV3 | | leukaemia | | 47 | 47 | 0.2 | 0 | 1.6 |
| Bzhalava | 2016 | Viremia during pregnancy and risk of childhood leukaemia and lymphomas in the offspring: Nested case-control study | Nested-Case Control | | TTMV4 | | leukaemia | | 47 | 47 | 0.7 | 0.2 | 3.5 |
| Bzhalava | 2016 | Viremia during pregnancy and risk of childhood leukaemia and lymphomas in the offspring: Nested case-control study | Nested-Case Control | | TTMV5 | | leukaemia | | 47 | 47 | 0.7 | 0.2 | 3.5 |
| Bzhalava | 2016 | Viremia during pregnancy and risk of childhood leukaemia and lymphomas in the offspring: Nested case-control study | Nested-Case Control | | TTMV6 | | leukaemia | | 47 | 47 | 0.3 | 0 | 3.2 |
| Bzhalava | 2016 | Viremia during pregnancy and risk of childhood leukaemia and lymphomas in the offspring: Nested case-control study | Nested-Case Control | | TTMV7 | | leukaemia | | 47 | 47 | 0.4 | 0.1 | 1.6 |
| Bzhalava | 2016 | Viremia during pregnancy and risk of childhood leukaemia and lymphomas in the offspring: Nested case-control study | Nested-Case Control | | TTMV8 | | leukaemia | | 47 | 47 | 0.4 | 0.1 | 1.4 |
| Bzhalava | 2016 | Viremia during pregnancy and risk of childhood leukaemia and lymphomas in the offspring: Nested case-control study | Nested-Case Control | | TTMV9 | | leukaemia | | 47 | 47 | 0.8 | 0.2 | 2.9 |
| Bzhalava | 2016 | Viremia during pregnancy and risk of childhood leukaemia and lymphomas in the offspring: Nested case-control study | Nested-Case Control | | TTMV10 | | leukaemia | | 47 | 47 | 0.2 | 0 | 1.6 |
| Bzhalava | 2016 | Viremia during pregnancy and risk of childhood leukaemia and lymphomas in the offspring: Nested case-control study | Nested-Case Control | | TTMV11 | | leukaemia | | 47 | 47 | 0.4 | 0.1 | 2 |
| Bzhalava | 2016 | Viremia during pregnancy and risk of childhood leukaemia and lymphomas in the offspring: Nested case-control study | Nested-Case Control | | TTMV12 | | leukaemia | | 47 | 47 | 1 | 0.2 | 5.2 |
| Bzhalava | 2016 | Viremia during pregnancy and risk of childhood leukaemia and lymphomas in the offspring: Nested case-control study | Nested-Case Control | | Betatorquevirus total | | leukaemia | | 47 | 47 | 0.6 | 0.2 | 1.5 |
| Bzhalava | 2016 | Viremia during pregnancy and risk of childhood leukaemia and lymphomas in the offspring: Nested case-control study | Nested-Case Control | | TTMDV1 | | leukaemia | | 47 | 47 | 0.6 | 0.2 | 1.6 |
| Bzhalava | 2016 | Viremia during pregnancy and risk of childhood leukaemia and lymphomas in the offspring: Nested case-control study | Nested-Case Control | | TTMDV2 | | leukaemia | | 47 | 47 | 0.6 | 0.2 | 1.6 |
| Bzhalava | 2016 | Viremia during pregnancy and risk of childhood leukaemia and lymphomas in the offspring: Nested case-control study | Nested-Case Control | | TTMDV3 | | leukaemia | | 47 | 47 | 0.5 | 0 | 5.6 |
| Bzhalava | 2016 | Viremia during pregnancy and risk of childhood leukaemia and lymphomas in the offspring: Nested case-control study | Nested-Case Control | | TTMDV5 | | leukaemia | | 47 | 47 | 1 | 0.3 | 3.4 |
| Bzhalava | 2016 | Viremia during pregnancy and risk of childhood leukaemia and lymphomas in the offspring: Nested case-control study | Nested-Case Control | | TTMDV6 | | leukaemia | | 47 | 47 | 1 | 0.1 | 16.5 |
| Bzhalava | 2016 | Viremia during pregnancy and risk of childhood leukaemia and lymphomas in the offspring: Nested case-control study | Nested-Case Control | | TTMDV7 | | leukaemia | | 47 | 47 | 0.5 | 0.1 | 2.7 |
| Bzhalava | 2016 | Viremia during pregnancy and risk of childhood leukaemia and lymphomas in the offspring: Nested case-control study | Nested-Case Control | | TTMDV8 | | leukaemia | | 47 | 47 | 1.7 | 0.4 | 7.8 |
| Bzhalava | 2016 | Viremia during pregnancy and risk of childhood leukaemia and lymphomas in the offspring: Nested case-control study | Nested-Case Control | | TTMDV9 | | leukaemia | | 47 | 47 |  |  |  |
| Bzhalava | 2016 | Viremia during pregnancy and risk of childhood leukaemia and lymphomas in the offspring: Nested case-control study | Nested-Case Control | | TTMDV10 | | leukaemia | | 47 | 47 | 0.7 | 0.2 | 3.5 |
| Bzhalava | 2016 | Viremia during pregnancy and risk of childhood leukaemia and lymphomas in the offspring: Nested case-control study | Nested-Case Control | | TTMDV11 | | leukaemia | | 47 | 47 |  |  |  |
| Bzhalava | 2016 | Viremia during pregnancy and risk of childhood leukaemia and lymphomas in the offspring: Nested case-control study | Nested-Case Control | | TTMDV13 | | leukaemia | | 47 | 47 | 0.2 | 0 | 2.2 |
| Bzhalava | 2016 | Viremia during pregnancy and risk of childhood leukaemia and lymphomas in the offspring: Nested case-control study | Nested-Case Control | | TTMDV14 | | leukaemia | | 47 | 47 | 0.6 | 0.2 | 1.9 |
| Bzhalava | 2016 | Viremia during pregnancy and risk of childhood leukaemia and lymphomas in the offspring: Nested case-control study | Nested-Case Control | | TTMDV15 | | leukaemia | | 47 | 47 |  |  |  |
| Bzhalava | 2016 | Viremia during pregnancy and risk of childhood leukaemia and lymphomas in the offspring: Nested case-control study | Nested-Case Control | | all gammatorqueviruses | | leukaemia | | 47 | 47 | 0.4 | 0.2 | 0.9 |
| Bzhalava | 2016 | Viremia during pregnancy and risk of childhood leukaemia and lymphomas in the offspring: Nested case-control study | Nested-Case Control | | all Anelloviridae | | leukaemia | | 47 | 47 | 0.6 | 0.2 | 1.9 |
| Bzhalava | 2016 | Viremia during pregnancy and risk of childhood leukaemia and lymphomas in the offspring: Nested case-control study | Nested-Case Control | | Anelloviridae from environmental samples | | leukaemia | | 47 | 47 | 0.2 | 0.02 | 1.6 |
| Bzhalava | 2016 | Viremia during pregnancy and risk of childhood leukaemia and lymphomas in the offspring: Nested case-control study | Nested-Case Control | | “Unclassified” viruses from environmental samples | | leukaemia | | 47 | 47 | 0.6 | 0.2 | 1.6 |
| Bzhalava | 2016 | Viremia during pregnancy and risk of childhood leukaemia and lymphomas in the offspring: Nested case-control study | Nested-Case Control | | All viruses from environmental samples | | leukaemia | | 47 | 47 | 0.4 | 0.1 | 1 |
| Bzhalava | 2016 | Viremia during pregnancy and risk of childhood leukaemia and lymphomas in the offspring: Nested case-control study | Nested-Case Control | | Papillomaviridae | | leukaemia | | 47 | 47 |  |  |  |
| Bzhalava | 2016 | Viremia during pregnancy and risk of childhood leukaemia and lymphomas in the offspring: Nested case-control study | Nested-Case Control | | Gemycircularvirus group | | leukaemia | | 47 | 47 | 1.7 | 0.6 | 4.5 |
| Bzhalava | 2016 | Viremia during pregnancy and risk of childhood leukaemia and lymphomas in the offspring: Nested case-control study | Nested-Case Control | | Other “unclassified” viruses | | leukaemia | | 47 | 47 |  |  |  |
| Bzhalava | 2016 | Viremia during pregnancy and risk of childhood leukaemia and lymphomas in the offspring: Nested case-control study | Nested-Case Control | | all “unclassified” viruses | | leukaemia | | 47 | 47 | 1.6 | 0.6 | 4.2 |
| Dockerty | 1999 | Infections, vaccinations, and the risk of childhood cancer | population-based case control | | Any infection (rubella, measles, chicken pox, shingles, mumps, glandular fever, pneumonia, hepatitis B, malaria, leptospirosis) | | leukaemia | | 121 | 303 | 1.45 | 0.55 | 3.82 |
| Dockerty | 1999 | Infections, vaccinations, and the risk of childhood cancer | population-based case control | | Cystitis or kidney infection | | leukaemia | | 121 | 303 | 0.5 | 0.14 | 1.85 |
| Dockerty | 1999 | Infections, vaccinations, and the risk of childhood cancer | population-based case control | | Cold sores/oral herpes | | leukaemia | | 121 | 303 | 0.98 | 0.38 | 2.5 |
| Dockerty | 1999 | Infections, vaccinations, and the risk of childhood cancer | population-based case control | | Any other infection | | leukaemia | | 121 | 303 | 1.45 | 0.59 | 3.57 |
| Dockerty | 1999 | Infections, vaccinations, and the risk of childhood cancer | population-based case control | | influenza | | leukaemia | | 121 | 303 | 0.58 | 0.24 | 1.41 |
| Dockerty | 1999 | Infections, vaccinations, and the risk of childhood cancer | population-based case control | | Cystitis or kidney infection | | leukaemia | | 121 | 303 | 0.56 | 0.15 | 2.16 |
| Dockerty | 1999 | Infections, vaccinations, and the risk of childhood cancer | Population-based Case-control | | Cold sores/oral herpes | | leukaemia | | 121 | 303 | 0.7 | 0.25 | 1.99 |
| Dockerty | 1999 | Malignant neoplasms of the brain during childhood: the role of prenatal and neonatal factors (United Kingdom) | Population-based Case-control | | Any other infection | | leukaemia | | 121 | 303 | 1.45 | 0.55 | 3.82 |
| Farwell | 1979 | Effect of SV40 Virus-contaminated Polio Vaccine on the Incidence and Type of CNS Neoplasms in children: a Population-Based Study | Population-based Case-Control | | SV40 virus | | CNS tumours | | 52 | 38 | 2.16 | 0.82 | 5.65 |
| Fear | 2001 | Malignant neoplasms of the brain during childhood: the role of prenatal and neonatal factors (United Kingdom) | Population-based Case-Control | | Infections (ICD-10: P35-P39) | | malignant neoplasms of the brain or other parts of nervous system | | 83 | 166 | 0.8 | 0.3 | 2.3 |
| Fear | 2001 | Malignant neoplasms of the brain during childhood: the role of prenatal and neonatal factors (United Kingdom) | Population-based Case-Control | | infection of definite viral origin | | malignant neoplasms of the brain or other parts of nervous system | | 83 | 166 | 10.6 | 1.1 | 503.2 |
| Fear | 2001 | Malignant neoplasms of the brain during childhood: the role of prenatal and neonatal factors (United Kingdom) | Population-based Case-Control | | infection of probable viral origin (includes respiratory infections and influenza) | | malignant neoplasms of the brain or other parts of nervous system | | 83 | 166 | 2.2 | 0.6 | 7.2 |
| Fedrick | 1972 | Reported influenza in pregnancy and subsequent cancer in the child | Prospective cohort | | influenza | | ALL | | 10 | 16730 | 11.36 | 3.2 | 40.28 |
| Fedrick | 1972 | Reported influenza in pregnancy and subsequent cancer in the child | Prospective cohort | | influenza | | leukaemia | | 11 | 16730 | 9.09 | 2.77 | 29.8 |
| Fedrick | 1972 | Reported influenza in pregnancy and subsequent cancer in the child | Prospective cohort | | influenza | | Wilm's Tumour | | 4 | 16730 | 2.51 | 0.26 | 24.19 |
| Fedrick | 1972 | Reported influenza in pregnancy and subsequent cancer in the child | Prospective cohort | | influenza | | lymphoma | | 2 | 16730 | 7.55 | 0.47 | 120.82 |
| Fedrick | 1972 | Reported influenza in pregnancy and subsequent cancer in the child | Prospective cohort | | influenza | | childhood cancer | | 20 | 16730 | 5.1 | 1.93 | 13.46 |
| Fedrick | 1972 | Reported influenza in pregnancy and subsequent cancer in the child | Prospective cohort | | influenza | | CNS tumours | | 3 | 16730 | 1.08 | 0.06 | 20.86 |
| Fine | 1985 | Long term effects of exposure to viral infections in utero | cohort study | | viral infection | | childhood cancer | | 14 | 5031 | 2.51 | 0.79 | 8.02 |
| Fine | 1985 | Long term effects of exposure to viral infections in utero | cohort study | | influenza | | ALL | | 2 | 5027 | 1.5 | 0.07 | 31.9 |
| Fine | 1985 | Long term effects of exposure to viral infections in utero | cohort study | | Varicella | | ALL | | 2 | 5027 | 13.57 | 0.85 | 217.45 |
| Fine | 1985 | Long term effects of exposure to viral infections in utero | cohort study | | CMV | | ALL | | 2 | 5027 | 52.34 | 3.25 | 843.06 |
| Fine | 1985 | Long term effects of exposure to viral infections in utero | cohort study | | mumps | | ALL | | 2 | 5027 | 1.71 | 0.08 | 35.74 |
| Fine | 1985 | Long term effects of exposure to viral infections in utero | cohort study | | rubella | | ALL | | 2 | 5027 | 1.08 | 0.05 | 22.59 |
| Fine | 1985 | Long term effects of exposure to viral infections in utero | cohort study | | Viral infection | | ALL | | 2 | 5039 | 4.82 | 0.23 | 100.43 |
| Fine | 1985 | Long term effects of exposure to viral infections in utero | cohort study | | Viral infection | | leukaemia | | 6 | 5039 | 1.93 | 0.35 | 10.53 |
| Fine | 1985 | Long term effects of exposure to viral infections in utero | cohort study | | influenza | | leukaemia | | 6 | 5039 | 0.59 | 0.03 | 10.46 |
| Fine | 1985 | Long term effects of exposure to viral infections in utero | cohort study | | influenza | | all cancers | | 14 | 5031 | 0.33 | 0.04 | 2.5 |
| Fine | 1985 | Long term effects of exposure to viral infections in utero | cohort study | | CMV | | Leukaemia | | 6 | 5027 | 10.27 | 1.19 | 88.77 |
| Fine | 1985 | Long term effects of exposure to viral infections in utero | cohort study | | Varicella | | Leukaemia | | 6 | 5039 | 0.48 | 0.09 | 2.63 |
| Fine | 1985 | Long term effects of exposure to viral infections in utero | cohort study | | any infection | | CNS tumours | | 2 | 5043 | 4.82 | 0.23 | 100.43 |
| Fine | 1985 | Long term effects of exposure to viral infections in utero | cohort study | | any infection | | solid tumours | | 6 | 5039 | 1.93 | 0.35 | 10.53 |
| Fine | 1985 | Long term effects of exposure to viral infections in utero | cohort study | | CMV | | all cancers | | 14 | 5031 | 4.32 | 0.95 | 19.58 |
| Fine | 1985 | Long term effects of exposure to viral infections in utero | cohort study | | rubella | | all cancers | | 14 | 5031 | 0.86 | 0.24 | 31.1 |
| Fine | 1985 | Long term effects of exposure to viral infections in utero | cohort study | | Varicella | | all cancers | | 14 | 5031 | 1.19 | 0.27 | 5.35 |
| Francis | 2017 | In utero cytomegalovirus infection and development of childhood acute lymphoblastic leukaemia | Nested case-control (cases&controls from same source) | | CMV | | ALL | | 268 | 270 | 3.71 | 1.71 | 8.95 |
| Francis | 2017 | In utero cytomegalovirus infection and development of childhood acute lymphoblastic leukaemia | Nested case-control (cases&controls from same source) | | EBV | | ALL | | 268 | 270 | 1.01 | 0.42 | 2.42 |
| Gardner | 1990 | Results of case-control study of leukaemia and lymphoma among young people near Sellafield nuclear plant in West Cumbria | population-based case control | | viral infection | | Leukaemia | | 35 | 103 | 1.23 | 0.22 | 7.04 |
| Gustafsson | 2007 | Adenovirus DNA is detected at increased frequency in Guthrie cards from children who develop acute lymphoblastic leukaemia | Case-control | | C adenovirus | | ALL | | 49 | 47 | 5.3 | 1.3 | 31 |
| Geris | 2023 | Evaluation of the Association Between Congenital Cytomegalovirus Infection and Paediatric Acute Lymphoblastic Leukaemia | Case-control | | CMV | | ALL | | 1189 | 4756 | 1.30 | 0.52 | 3.24 |
| Hamrik | 2001 | Association of pregnancy history and birth characteristics with neuroblastoma: a report from the Children's Cancer Group and the Paediatric Oncology Group | Population-based Case-Control | | Influenza in the month prior to pregnancy | | neuroblastoma | | 504 | 504 | 2.3 | 1.2 | 4.7 |
| Hamrik | 2001 | Association of pregnancy history and birth characteristics with neuroblastoma: a report from the Children's Cancer Group and the Paediatric Oncology Group | Population-based Case-Control | | Influenza during the entire pregnancy period | | neuroblastoma | | 504 | 504 | 0.9 | 0.7 | 1.4 |
| Hamrik | 2001 | Association of pregnancy history and birth characteristics with neuroblastoma: a report from the Children's Cancer Group and the Paediatric Oncology Group | Population-based Case-Control | | chicken pox | | neuroblastoma | | 504 | 504 | 13.1 | 0.74 | 234.18 |
| Hamrik | 2001 | Association of pregnancy history and birth characteristics with neuroblastoma: a report from the Children's Cancer Group and the Paediatric Oncology Group | Population-based Case-Control | | Urinary tract infection | | neuroblastoma | | 504 | 504 | 1.2 | 0.8 | 1.7 |
| Hamrik | 2001 | Association of pregnancy history and birth characteristics with neuroblastoma: a report from the Children's Cancer Group and the Paediatric Oncology Group | Population-based Case-Control | | vaginitis | | neuroblastoma | | 504 | 504 | 1 | 0.7 | 1.6 |
| Hamrik | 2001 | Association of pregnancy history and birth characteristics with neuroblastoma: a report from the Children's Cancer Group and the Paediatric Oncology Group | Population-based Case-Control | | STD | | neuroblastoma | | 504 | 504 | 3.1 | 1.4 | 7 |
| He | 2023 | Evaluation of Maternal Infection During Pregnancy and Childhood Leukaemia Among Offspring in Denmark | population-based Cohort | | any infection | | Leukaemia | | 1307 | 2221490 | 1.35 | 1.04 | 1.77 |
| He | 2023 | Evaluation of Maternal Infection During Pregnancy and Childhood Leukaemia Among Offspring in Denmark | population-based Cohort | | genitourinary tract infection | | Leukaemia | | 1307 | 2221490 | 1.74 | 1.29 | 2.35 |
| He | 2023 | Evaluation of Maternal Infection During Pregnancy and Childhood Leukaemia Among Offspring in Denmark | population-based Cohort | | genitourinary tract infection | | Leukaemia | | 1307 | 2221490 | 1.65 | 1.15 | 2.36 |
| He | 2023 | Evaluation of Maternal Infection During Pregnancy and Childhood Leukaemia Among Offspring in Denmark | population-based Cohort | | genitourinary tract infection | | Leukaemia | | 1307 | 2221490 | 2.42 | 1.5 | 3.92 |
| He | 2023 | Evaluation of Maternal Infection During Pregnancy and Childhood Leukaemia Among Offspring in Denmark | population-based Cohort | | sexually transmitted infection | | Leukaemia | | 1307 | 2218435 | 3.13 | 1.73 | 5.67 |
| He | 2023 | Evaluation of Maternal Infection During Pregnancy and Childhood Leukaemia Among Offspring in Denmark | population-based Cohort | | respiratory tract infection | | Leukaemia | | 1307 | 2218435 | 0.89 | 0.26 | 2.5 |
| He | 2023 | Evaluation of Maternal Infection During Pregnancy and Childhood Leukaemia Among Offspring in Denmark | population-based Cohort | | digestive tract infection | | Leukaemia | | 1307 | 2218435 | 0.89 | 0.37 | 2.15 |
| He | 2023 | Evaluation of Maternal Infection During Pregnancy and Childhood Leukaemia Among Offspring in Denmark | population-based Cohort | | other infections | | Leukaemia | | 1307 | 2218435 | 0.93 | 0.48 | 1.8 |
| He | 2023 | Evaluation of Maternal Infection During Pregnancy and Childhood Leukaemia Among Offspring in Denmark | population-based Cohort | | any infection | | brain tumour | | 1267 | 2218435 | 1.24 | 0.94 | 1.65 |
| He | 2023 | Evaluation of Maternal Infection During Pregnancy and Childhood Leukaemia Among Offspring in Denmark | population-based Cohort | | genitourinary tract infection | | brain tumour | | 1267 | 2218435 | 1.21 | 0.83 | 1.75 |
| He | 2023 | Evaluation of Maternal Infection During Pregnancy and Childhood Leukaemia Among Offspring in Denmark | population-based Cohort | | Urinary tract infection | | brain tumour | | 1267 | 2218435 | 1.21 | 0.78 | 1.86 |
| He | 2023 | Evaluation of Maternal Infection During Pregnancy and Childhood Leukaemia Among Offspring in Denmark | population-based Cohort | | genital tract | | brain tumour | | 1267 | 2218435 | 1.28 | 0.67 | 2.47 |
| He | 2023 | Evaluation of Maternal Infection During Pregnancy and Childhood Leukaemia Among Offspring in Denmark | population-based Cohort | | respiratory tract infection | | brain tumour | | 1267 | 2218435 | 1.93 | 0.92 | 4.05 |
| He | 2023 | Evaluation of Maternal Infection During Pregnancy and Childhood Leukaemia Among Offspring in Denmark | population-based Cohort | | digestive tract infection | | brain tumour | | 1267 | 2218435 | 1.1 | 0.49 | 2.45 |
| He | 2023 | Evaluation of Maternal Infection During Pregnancy and Childhood Leukaemia Among Offspring in Denmark | population-based Cohort | | other infections | | brain tumour | | 1267 | 2218435 | 1.2 | 0.66 | 2.18 |
| He | 2023 | Evaluation of Maternal Infection During Pregnancy and Childhood Leukaemia Among Offspring in Denmark | population-based Cohort | | any infection | | lymphoma | | 224 | 2218435 | 1.29 | 0.7 | 2.37 |
| He | 2023 | Evaluation of Maternal Infection During Pregnancy and Childhood Leukaemia Among Offspring in Denmark | population-based Cohort | | genitourinary tract infection | | lymphoma | | 224 | 2218435 | 1.32 | 0.62 | 2.81 |
| He | 2023 | Evaluation of Maternal Infection During Pregnancy and Childhood Leukaemia Among Offspring in Denmark | population-based Cohort | | Urinary tract infection | | lymphoma | | 224 | 2218435 | 0.99 | 0.37 | 2.66 |
| He | 2023 | Evaluation of Maternal Infection During Pregnancy and Childhood Leukaemia Among Offspring in Denmark | population-based Cohort | | genital tract infection | | lymphoma | | 224 | 2218435 | 2.2 | 0.7 | 6.89 |
| He | 2023 | Evaluation of Maternal Infection During Pregnancy and Childhood Leukaemia Among Offspring in Denmark | population-based Cohort | | respiratory tract infection | | lymphoma | | 224 | 2218435 | 2.76 | 0.69 | 11.13 |
| He | 2023 | Evaluation of Maternal Infection During Pregnancy and Childhood Leukaemia Among Offspring in Denmark | population-based Cohort | | other infections | | lymphoma | | 224 | 2218435 | 1.09 | 0.27 | 4.38 |
| He | 2023 | Evaluation of Maternal Infection During Pregnancy and Childhood Leukaemia Among Offspring in Denmark | population-based Cohort | | any infection | | other cancer (not including leukaemia) | | 1564 | 2218435 | 1.09 | 0.83 | 1.43 |
| He | 2023 | Evaluation of Maternal Infection During Pregnancy and Childhood Leukaemia Among Offspring in Denmark | population-based Cohort | | genitourinary tract infection | | other cancer (not including leukaemia) | | 1564 | 2218435 | 1.17 | 0.84 | 1.64 |
| He | 2023 | Evaluation of Maternal Infection During Pregnancy and Childhood Leukaemia Among Offspring in Denmark | population-based Cohort | | Urinary tract infection | | other cancer (not including leukaemia) | | 1564 | 2218435 | 1.21 | 0.82 | 1.78 |
| He | 2023 | Evaluation of Maternal Infection During Pregnancy and Childhood Leukaemia Among Offspring in Denmark | population-based Cohort | | genital tract infection | | other cancer (not including leukaemia) | | 1564 | 2218435 | 1.14 | 0.61 | 2.13 |
| He | 2023 | Evaluation of Maternal Infection During Pregnancy and Childhood Leukaemia Among Offspring in Denmark | population-based Cohort | | respiratory tract infection | | other cancer (not including leukaemia) | | 1564 | 2218435 | 0.45 | 0.11 | 1.78 |
| He | 2023 | Evaluation of Maternal Infection During Pregnancy and Childhood Leukaemia Among Offspring in Denmark | population-based Cohort | | digestive tract infection | | other cancer (not including leukaemia) | | 1564 | 2218435 | 0.75 | 0.31 | 1.8 |
| He | 2023 | Evaluation of Maternal Infection During Pregnancy and Childhood Leukaemia Among Offspring in Denmark | population-based Cohort | | other infections | | other cancer (not including leukaemia) | | 1564 | 2218435 | 1.22 | 0.72 | 2.06 |
| Heck | 2012 | Perinatal characteristics and retinoblastoma | population-based case control | | sexually transmitted disease | | all retinoblastoma | | 609 | 209051 | 1.8 | 0.96 | 3.38 |
| Heck | 2012 | Perinatal characteristics and retinoblastoma | population-based case control | | sexually transmitted disease | | retinoblastoma | | 420 | 209051 | 1.03 | 0.39 | 2.78 |
| Heck | 2012 | Perinatal characteristics and retinoblastoma | population-based case control | | sexually transmitted disease | | retinoblastoma | | 187 | 209051 | 3.59 | 1.58 | 8.15 |
| Heck | 2012 | Perinatal characteristics and retinoblastoma | population-based case control | | sexually transmitted disease - genital herpes | | retinoblastoma | | 187 | 209051 | 3.06 | 0.97 | 9.67 |
| Heck | 2012 | Perinatal characteristics and retinoblastoma | population-based case control | | other sexually transmitted disease | | retinoblastoma | | 187 | 209051 | 4.04 | 1.28 | 12.74 |
| Heck | 2021 | Cohort study of familial viral hepatitis and risks of paediatric cancers | cohort study | | hepatitis B virus | | all cancers | | 2160 | 2076877 | 0.91 | 0.69 | 1.2 |
| Heck | 2021 | Cohort study of familial viral hepatitis and risks of paediatric cancers | cohort study | | hepatitis B virus | | all leukemias | | 710 | 2076877 | 0.79 | 0.49 | 1.28 |
| Heck | 2021 | Cohort study of familial viral hepatitis and risks of paediatric cancers | cohort study | | hepatitis B virus | | ALL | | 529 | 2076877 | 0.75 | 0.42 | 1.32 |
| Heck | 2021 | Cohort study of familial viral hepatitis and risks of paediatric cancers | cohort study | | hepatitis B virus | | AML | |  | 2076877 | 1.24 | 0.5 | 3.05 |
| Heck | 2021 | Cohort study of familial viral hepatitis and risks of paediatric cancers | cohort study | | hepatitis B virus | | NHL | |  | 2076877 | 0.38 | 0.16 | 0.91 |
| Heck | 2021 | Cohort study of familial viral hepatitis and risks of paediatric cancers | cohort study | | hepatitis B virus | | CNS tumours | | 245 | 2076877 | 1.09 | 0.54 | 2.2 |
| Heck | 2021 | Cohort study of familial viral hepatitis and risks of paediatric cancers | cohort study | | hepatitis B virus | | neuroblastoma | |  | 2076877 | 1.22 | 0.6 | 2.48 |
| Heck | 2021 | Cohort study of familial viral hepatitis and risks of paediatric cancers | cohort study | | hepatitis B virus | | all hepatic tumours | |  | 2076877 | 1.2 | 0.51 | 2.8 |
| Heck | 2021 | Cohort study of familial viral hepatitis and risks of paediatric cancers | cohort study | | hepatitis B virus | | hepatoblastoma | | 168 | 2076877 | 1.4 | 0.56 | 3.52 |
| Heck | 2021 | Cohort study of familial viral hepatitis and risks of paediatric cancers | cohort study | | hepatitis B virus | | germ cell tumours | |  | 2076877 | 0.58 | 0.19 | 1.82 |
| Heck | 2021 | Cohort study of familial viral hepatitis and risks of paediatric cancers | cohort study | | hepatitis C virus | | all cancers | | 2160 | 2076877 | 1.17 | 0.58 | 2.34 |
| Heck | 2015 | A case–control study of sporadic retinoblastoma in relation to maternal health conditions and reproductive factors: a report from the Children’s Oncology group | Case-control | | any infection | | unilateral retinoblastoma | | 165 | 136 | 1 | 0.5 | 1.7 |
| Heck | 2015 | A case–control study of sporadic retinoblastoma in relation to maternal health conditions and reproductive factors: a report from the Children’s Oncology group | Case-control | | respiratory infection | | unilateral retinoblastoma | | 165 | 136 | 0.9 | 0.4 | 2 |
| Heck | 2015 | A case–control study of sporadic retinoblastoma in relation to maternal health conditions and reproductive factors: a report from the Children’s Oncology group | Case-control | | flu or cold | | unilateral retinoblastoma | | 165 | 136 | 1.2 | 0.5 | 3.1 |
| Heck | 2015 | A case–control study of sporadic retinoblastoma in relation to maternal health conditions and reproductive factors: a report from the Children’s Oncology group | Case-control | | other viral infections | | unilateral retinoblastoma | | 165 | 136 | 3.6 | 0.3 | 47.9 |
| Heck | 2015 | A case–control study of sporadic retinoblastoma in relation to maternal health conditions and reproductive factors: a report from the Children’s Oncology group | Case-control | | all bacterial infections | | unilateral retinoblastoma | | 165 | 136 | 1.1 | 0.5 | 2.4 |
| Heck | 2015 | A case–control study of sporadic retinoblastoma in relation to maternal health conditions and reproductive factors: a report from the Children’s Oncology group | Case-control | | Urinary tract infection | | unilateral retinoblastoma | | 165 | 136 | 0.8 | 0.2 | 3 |
| Heck | 2015 | A case–control study of sporadic retinoblastoma in relation to maternal health conditions and reproductive factors: a report from the Children’s Oncology group | case-control | | any infectious disease | | bilateral retinoblastoma | | 87 | 136 | 0.8 | 0.4 | 1.6 |
| Heck | 2015 | A case–control study of sporadic retinoblastoma in relation to maternal health conditions and reproductive factors: a report from the Children’s Oncology group | case-control | | respiratory infection | | bilateral retinoblastoma | | 87 | 136 | 0.4 | 0.1 | 1.2 |
| Heck | 2015 | A case–control study of sporadic retinoblastoma in relation to maternal health conditions and reproductive factors: a report from the Children’s Oncology group | case-control | | flu or cold | | bilateral retinoblastoma | | 87 | 136 | 0.3 | 0.1 | 1.4 |
| Heck | 2015 | A case–control study of sporadic retinoblastoma in relation to maternal health conditions and reproductive factors: a report from the Children’s Oncology group | case-control | | other viral infections | | bilateral retinoblastoma | | 87 | 136 | 3.7 | 0.3 | 3.5 |
| Heck | 2015 | A case–control study of sporadic retinoblastoma in relation to maternal health conditions and reproductive factors: a report from the Children’s Oncology group | case-control | | all bacterial infections | | bilateral retinoblastoma | | 87 | 136 | 0.8 | 0.3 | 2.1 |
| Heck | 2015 | A case–control study of sporadic retinoblastoma in relation to maternal health conditions and reproductive factors: a report from the Children’s Oncology group | case-control | | Urinary tract infection | | bilateral retinoblastoma | | 87 | 136 | 0.2 | 0.1 | 1.8 |
| Heininonen | 1973 | Immunization During Pregnancy Against Poliomyelitis and Influenza in Relation to Childhood Malignancy | Prospective cohort | | spontaneous viral infection | | any malignancy | | 24 | 50873 | 0.32 | 0.02 | 5.26 |
| Heininonen | 1973 | Immunization During Pregnancy Against Poliomyelitis and Influenza in Relation to Childhood Malignancy | Prospective cohort | | spontaneous viral infection | | Leukaemia | | 8 | 50873 | 0.92 | 0.05 | 15.98 |
| Heininonen | 1973 | Immunization During Pregnancy Against Poliomyelitis and Influenza in Relation to Childhood Malignancy | Prospective cohort | | spontaneous viral infection | | ALL | | 2 | 50873 | 3.13 | 0.15 | 65.3 |
| Heininonen | 1973 | Immunization During Pregnancy Against Poliomyelitis and Influenza in Relation to Childhood Malignancy | Prospective cohort | | spontaneous viral infection | | CNS and neuroblastomas | | 7 | 50873 | 1.04 | 0.06 | 18.3 |
| Heininonen | 1973 | Immunization During Pregnancy Against Poliomyelitis and Influenza in Relation to Childhood Malignancy | Prospective cohort | | spontaneous viral infection | | Solid tumours | | 9 | 50873 | 0.82 | 0.05 | 14.18 |
| Holl | 2008 | Maternal Epstein-Barr virus and cytomegalovirus infections and risk of testicular cancer in the offspring: a nested case-control study | nested case-control | | Epstein-Barr virus IgM seropositive | | testicular cancer | | 66 | 258 |  |  |  |
| Holl | 2008 | Maternal Epstein-Barr virus and cytomegalovirus infections and risk of testicular cancer in the offspring: a nested case-control study | nested case-control | | Epstein-Barr virus IgM low antibody level | | testicular cancer | | 66 | 258 |  |  |  |
| Holl | 2008 | Maternal Epstein-Barr virus and cytomegalovirus infections and risk of testicular cancer in the offspring: a nested case-control study | nested case-control | | Epstein-Barr virus IgM high antibody level | | testicular cancer | | 66 | 258 |  |  |  |
| Holl | 2008 | Maternal Epstein-Barr virus and cytomegalovirus infections and risk of testicular cancer in the offspring: a nested case-control study | nested case-control | | Epstein-Barr virus - IgG high antibody level | | testicular cancer | | 66 | 258 | 2.5 | 1.15 | 5.4 |
| Holl | 2008 | Maternal Epstein-Barr virus and cytomegalovirus infections and risk of testicular cancer in the offspring: a nested case-control study | nested case-control | | Cytomegalovirus IgM seropositive | | testicular cancer | | 66 | 258 | 0.57 | 0.07 | 4.64 |
| Holl | 2008 | Maternal Epstein-Barr virus and cytomegalovirus infections and risk of testicular cancer in the offspring: a nested case-control study | nested case-control | | Cytomegalovirus - IgM Low antibody level | | testicular cancer | | 66 | 258 | 1.33 | 0.14 | 12.81 |
| Holl | 2008 | Maternal Epstein-Barr virus and cytomegalovirus infections and risk of testicular cancer in the offspring: a nested case-control study | nested case-control | | Cytomegalovirus - IgM seropositive high antibody level | | testicular cancer | | 66 | 258 |  |  |  |
| Holl | 2008 | Maternal Epstein-Barr virus and cytomegalovirus infections and risk of testicular cancer in the offspring: a nested case-control study | nested case-control | | CMV - IgG Seropositive | | testicular cancer | | 66 | 258 | 0.95 | 0.48 | 1.86 |
| Holl | 2008 | Maternal Epstein-Barr virus and cytomegalovirus infections and risk of testicular cancer in the offspring: a nested case-control study | nested case-control | | CMV - IgG low antibody level | | testicular cancer | | 66 | 258 | 0.89 | 0.42 | 1.87 |
| Holl | 2008 | Maternal Epstein-Barr virus and cytomegalovirus infections and risk of testicular cancer in the offspring: a nested case-control study | nested case-control | | CMV - IgG high antibody level | | testicular cancer | | 66 | 258 | 1.02 | 0.48 | 2.18 |
| Holl | 2008 | Maternal Epstein-Barr virus and cytomegalovirus infections and risk of testicular cancer in the offspring: a nested case-control study | nested case-control | | EBV-IgG (low and high antibody levels) | | testicular cancer | | 66 | 258 | 2.72 | 1.05 | 7.04 |
| Holl | 2008 | Maternal Epstein-Barr virus and cytomegalovirus infections and risk of testicular cancer in the offspring: a nested case-control study | nested case-control | | EBV-IgG (low and high antibody levels) | | testicular cancer | | 66 | 258 | 2.96 | 0.88 | 9.39 |
| Holl | 2008 | Maternal Epstein-Barr virus and cytomegalovirus infections and risk of testicular cancer in the offspring: a nested case-control study | nested case-control | | CMV-IgM | | testicular cancer | | 66 | 258 | 1 | 0.11 | 8.94 |
| Holl | 2008 | Maternal Epstein-Barr virus and cytomegalovirus infections and risk of testicular cancer in the offspring: a nested case-control study | nested case-control | | CMV-IgG | | testicular cancer | | 66 | 258 | 0.35 | 0.14 | 0.89 |
| Holl | 2008 | Maternal Epstein-Barr virus and cytomegalovirus infections and risk of testicular cancer in the offspring: a nested case-control study | nested case-control | | CMV-IgG | | testicular cancer | | 66 | 258 | 2.97 | 0.87 | 10.17 |
| Holl | 2008 | Maternal Epstein-Barr virus and cytomegalovirus infections and risk of testicular cancer in the offspring: a nested case-control study | nested case-control | | EBV - IgG | | testicular cancer - non-seminoma | | 66 | 258 | 2.73 | 1.25 | 5.99 |
| Holl | 2008 | Maternal Epstein-Barr virus and cytomegalovirus infections and risk of testicular cancer in the offspring: a nested case-control study | nested case-control | | EBV - IgG | | testicular cancer - non-seminoma | | 66 | 258 | 2.72 | 1.05 | 7.04 |
| Holl | 2008 | Maternal Epstein-Barr virus and cytomegalovirus infections and risk of testicular cancer in the offspring: a nested case-control study | nested case-control | | EBV - IgG | | testicular cancer - non-seminoma | | 66 | 258 | 2.75 | 0.69 | 11.01 |
| Holl | 2008 | Maternal Epstein-Barr virus and cytomegalovirus infections and risk of testicular cancer in the offspring: a nested case-control study | nested case-control | | EBV - IgG | | testicular cancer - seminoma | | 66 | 258 | 3.68 | 0.31 | 43.26 |
| Holl | 2008 | Maternal Epstein-Barr virus and cytomegalovirus infections and risk of testicular cancer in the offspring: a nested case-control study | nested case-control | | EBV - IgG | | testicular cancer - seminoma | | 66 | 258 | 3.68 | 0.31 | 43.26 |
| Holl | 2008 | Maternal Epstein-Barr virus and cytomegalovirus infections and risk of testicular cancer in the offspring: a nested case-control study | nested case-control | | CMV-IgM | | testicular cancer non-seminoma | | 66 | 258 | 0.66 | 0.08 | 5.53 |
| Holl | 2008 | Maternal Epstein-Barr virus and cytomegalovirus infections and risk of testicular cancer in the offspring: a nested case-control study | nested case-control | | CMV-IgM | | testicular cancer non-seminoma | | 66 | 258 | 1.33 | 0.14 | 12.81 |
| Holl | 2008 | Maternal Epstein-Barr virus and cytomegalovirus infections and risk of testicular cancer in the offspring: a nested case-control study | nested case-control | | CMV-IgG | | testicular cancer non-seminoma | | 66 | 258 | 0.8 | 0.39 | 1.64 |
| Holl | 2008 | Maternal Epstein-Barr virus and cytomegalovirus infections and risk of testicular cancer in the offspring: a nested case-control study | nested case-control | | CMV-IgG | | testicular cancer non-seminoma | | 66 | 258 | 0.35 | 0.14 | 0.89 |
| Holl | 2008 | Maternal Epstein-Barr virus and cytomegalovirus infections and risk of testicular cancer in the offspring: a nested case-control study | nested case-control | | CMV-IgG | | testicular cancer non-seminoma | | 66 | 258 | 3.08 | 0.68 | 13.94 |
| Honkaniemi | 2010 | Adenovirus DNA in Guthrie cards from children who develop acute lymphoblastic leukaemia (ALL) | Case-Control | | C adenovirus DNA | | ALL | | 243 | 484 | 10.03 | 0.48 | 209.77 |
| Kumar | 2014 | Maternal factors and risk of childhood leukaemia | population-based Case-Control | | infection | | leukaemia | | 132 | 132 | 0.86 | 0.4 | 1.84 |
| Kwan | 2007 | Maternal illness and drug/medication use during the period surrounding pregnancy and risk of childhood leukaemia among offspring | population-based Case-Control | | Influenza/pneumonia | | leukaemia | | 365 | 460 | 1.77 | 1.17 | 2.68 |
| Kwan | 2007 | Maternal illness and drug/medication use during the period surrounding pregnancy and risk of childhood leukaemia among offspring | population-based Case-Control | | Urinary tract infection | | leukaemia | | 365 | 460 | 0.68 | 0.42 | 1.09 |
| Kwan | 2007 | Maternal illness and drug/medication use during the period surrounding pregnancy and risk of childhood leukaemia among offspring | population-based Case-Control | | sexually transmitted diseases (chlamydia, genital herpes, and human papillomavirus) | | leukaemia | | 365 | 460 | 7.59 | 1.58 | 36.56 |
| Kwan | 2007 | Maternal illness and drug/medication use during the period surrounding pregnancy and risk of childhood leukaemia among offspring | population-based Case-Control | | Influenza/pneumonia | | ALL | |  |  | 2.02 | 1.28 | 3.18 |
| Kwan | 2007 | Maternal illness and drug/medication use during the period surrounding pregnancy and risk of childhood leukaemia among offspring | population-based Case-Control | | Urinary tract infection | | ALL | |  |  | 0.7 | 0.42 | 1.17 |
| Kwan | 2007 | Maternal illness and drug/medication use during the period surrounding pregnancy and risk of childhood leukaemia among offspring | population-based Case-Control | | sexually transmitted diseases (chlamydia, genital herpes, and human papillomavirus) | | ALL | |  |  | 6.65 | 1.37 | 32.28 |
| Kwan | 2007 | Maternal illness and drug/medication use during the period surrounding pregnancy and risk of childhood leukaemia among offspring | population-based Case-Control | | Influenza/pneumonia | | common ALL | |  |  | 1.36 | 0.7 | 2.65 |
| Kwan | 2007 | Maternal illness and drug/medication use during the period surrounding pregnancy and risk of childhood leukaemia among offspring | population-based Case-Control | | Urinary tract infection | | common ALL | |  |  | 0.78 | 0.36 | 1.68 |
| Kwan | 2007 | Maternal illness and drug/medication use during the period surrounding pregnancy and risk of childhood leukaemia among offspring | population-based Case-Control | | sexually transmitted diseases (chlamydia, genital herpes, and human papillomavirus) | | common ALL | | 365 | 460 | 13.58 | 0.93 | 198.71 |
| Lehtinen | 2003 | Maternal herpesvirus infections and risk of acute lymphoblastic leukaemia in the offspring | Nested Case-control | | CMV IgG | | ALL | |  |  | 0.9 | 0.7 | 1.2 |
| Lehtinen | 2003 | Maternal herpesvirus infections and risk of acute lymphoblastic leukaemia in the offspring | Nested Case-control | | CMV IgM | | ALL | |  |  | 1.3 | 0.9 | 1.9 |
| Lehtinen | 2003 | Maternal herpesvirus infections and risk of acute lymphoblastic leukaemia in the offspring | Nested Case-control | | CMV IgG | | non-ALL | |  |  | 1.3 | 0.6 | 2.8 |
| Lehtinen | 2003 | Maternal herpesvirus infections and risk of acute lymphoblastic leukaemia in the offspring | Nested Case-control | | CMV IgM | | non-ALL | |  |  | 0.5 | 0.1 | 1.6 |
| Lehtinen | 2003 | Maternal herpesvirus infections and risk of acute lymphoblastic leukaemia in the offspring | Nested Case-control | | CMV IgG | | leukaemia total | | 403 | 1216 | 0.9 | 0.7 | 1.2 |
| Lehtinen | 2003 | Maternal herpesvirus infections and risk of acute lymphoblastic leukaemia in the offspring | Nested Case-control | | CMV IgG | | leukaemia total | | 403 | 1216 | 1.1 | 0.8 | 1.6 |
| Lehtinen | 2003 | Maternal herpesvirus infections and risk of acute lymphoblastic leukaemia in the offspring | Nested-Case Control | | EBV IgG | | ALL | |  |  | 1.6 | 0.8 | 3.2 |
| Lehtinen | 2003 | Maternal herpesvirus infections and risk of acute lymphoblastic leukaemia in the offspring | Nested-Case Control | | EBV IgM | | ALL | |  |  | 1.8 | 1.1 | 2.9 |
| Lehtinen | 2003 | Maternal herpesvirus infections and risk of acute lymphoblastic leukaemia in the offspring | Nested-Case Control | | EBV IgG | | non-ALL | |  |  | 1.2 | 0.3 | 4.4 |
| Lehtinen | 2003 | Maternal herpesvirus infections and risk of acute lymphoblastic leukaemia in the offspring | Nested-Case Control | | EBV IgM | | non-ALL | |  |  | 3.2 | 0.8 | 12 |
| Lehtinen | 2003 | Maternal herpesvirus infections and risk of acute lymphoblastic leukaemia in the offspring | Nested-Case Control | | EBV IgG | | leukaemia total | | 403 | 1216 | 1.5 | 0.8 | 2.8 |
| Lehtinen | 2003 | Maternal herpesvirus infections and risk of acute lymphoblastic leukaemia in the offspring | Nested-Case Control | | EBV IgM | | leukaemia total | | 403 | 1216 | 1.9 | 1.2 | 3 |
| Lehtinen | 2003 | Maternal herpesvirus infections and risk of acute lymphoblastic leukaemia in the offspring | Nested-Case Control | | EBV IgM | | ALL and other infant leukemias | |  |  | 2.9 | 1.5 | 5.8 |
| Lehtinen | 2003 | Maternal herpesvirus infections and risk of acute lymphoblastic leukaemia in the offspring | Nested-Case Control | | Human herpes virus 6 IgG | | ALL | |  |  | 0.8 | 0.6 | 1.1 |
| Lehtinen | 2003 | Maternal herpesvirus infections and risk of acute lymphoblastic leukaemia in the offspring | Nested-Case Control | | human herpes virus 6 IgM | | ALL | |  |  | 0.6 | 0.8 | 1.2 |
| Lehtinen | 2003 | Maternal herpesvirus infections and risk of acute lymphoblastic leukaemia in the offspring | Nested-Case Control | | human herpes virus 6 IgG | | non-ALL | |  |  | 1 | 0.5 | 2 |
| Lehtinen | 2003 | Maternal herpesvirus infections and risk of acute lymphoblastic leukaemia in the offspring | Nested-Case Control | | human herpes virus 6 IgM | | non-ALL | |  |  | 1 | 0.5 | 2.1 |
| Lehtinen | 2003 | Maternal herpesvirus infections and risk of acute lymphoblastic leukaemia in the offspring | Nested-Case Control | | human herpes virus 6 IgG | | leukaemia total | | 403 | 1216 | 0.8 | 0.6 | 1.1 |
| Lehtinen | 2003 | Maternal herpesvirus infections and risk of acute lymphoblastic leukaemia in the offspring | Nested-Case Control | | human herpes virus 6 IgM | | leukaemia total | | 403 | 1216 | 0.8 | 0.6 | 1.2 |
| Lehtinen | 2005 | Associations between three types of maternal bacterial infection and risk of leukaemia in the offspring | Nested Case-control | | Mycoplasma pneumoniae IgG | | ALL | |  |  | 8 | 0 | ∞ |
| Lehtinen | 2005 | Associations between three types of maternal bacterial infection and risk of leukaemia in the offspring | Nested Case-control | | Mycoplasma pneumoniae IgG | | ALL | |  |  | 1.2 | 0.8 | 1.6 |
| Lehtinen | 2005 | Associations between three types of maternal bacterial infection and risk of leukaemia in the offspring | Nested Case-control | | Mycoplasma pneumoniae IgM | | ALL | |  |  | 1.7 | 1 | 2.6 |
| Lehtinen | 2005 | Associations between three types of maternal bacterial infection and risk of leukaemia in the offspring | Nested Case-control | | Mycoplasma pneumoniae IgG | | ALL | |  |  | 1.2 | 0.9 | 1.7 |
| Lehtinen | 2005 | Associations between three types of maternal bacterial infection and risk of leukaemia in the offspring | Nested Case-control | | Mycoplasma pneumoniae IgM | | ALL | |  |  | 1.6 | 1 | 2.6 |
| Lehtinen | 2005 | Associations between three types of maternal bacterial infection and risk of leukaemia in the offspring | Nested Case-control | | Mycoplasma pneumoniae IgG | | non-ALL | |  |  | 0.3 | 0 | 2.3 |
| Lehtinen | 2005 | Associations between three types of maternal bacterial infection and risk of leukaemia in the offspring | Nested Case-control | | Mycoplasma pneumoniae IgG | | non-ALL | |  |  | 1.3 | 0.6 | 2.8 |
| Lehtinen | 2005 | Associations between three types of maternal bacterial infection and risk of leukaemia in the offspring | Nested Case-control | | Mycoplasma pneumoniae IgM | | non-ALL | |  |  | 2.2 | 0.6 | 8.2 |
| Lehtinen | 2005 | Associations between three types of maternal bacterial infection and risk of leukaemia in the offspring | Nested Case-control | | Mycoplasma pneumoniae IgG | | non-ALL | |  |  | 1.1 | 0.6 | 2.3 |
| Lehtinen | 2005 | Associations between three types of maternal bacterial infection and risk of leukaemia in the offspring | Nested Case-control | | Mycoplasma pneumoniae IgM | | non-ALL | |  |  | 1.5 | 0.4 | 5 |
| Lehtinen | 2005 | Associations between three types of maternal bacterial infection and risk of leukaemia in the offspring | Nested Case-control | | Mycoplasma pneumoniae IgG | | total leukemias | | 402 | 1212 | 1.3 | 0.3 | 6.2 |
| Lehtinen | 2005 | Associations between three types of maternal bacterial infection and risk of leukaemia in the offspring | Nested Case-control | | Mycoplasma pneumoniae IgG | | total leukemias | | 402 | 1212 | 1.2 | 0.9 | 1.6 |
| Lehtinen | 2005 | Associations between three types of maternal bacterial infection and risk of leukaemia in the offspring | Nested Case-control | | Mycoplasma pneumoniae IgM | | total leukemias | | 402 | 1212 | 1.8 | 1.1 | 2.8 |
| Lehtinen | 2005 | Associations between three types of maternal bacterial infection and risk of leukaemia in the offspring | Nested Case-control | | Mycoplasma pneumoniae IgG | | total leukemias | | 402 | 1212 | 1.2 | 0.9 | 1.6 |
| Lehtinen | 2005 | Associations between three types of maternal bacterial infection and risk of leukaemia in the offspring | Nested Case-control | | Mycoplasma pneumoniae IgM | | total leukemias | | 402 | 1212 | 1.6 | 1 | 2.5 |
| Lehtinen | 2005 | Associations between three types of maternal bacterial infection and risk of leukaemia in the offspring | Nested Case-control | | Chlamydia trachomatis IgG | | ALL | |  |  | 2.2 | 0.7 | 6.8 |
| Lehtinen | 2005 | Associations between three types of maternal bacterial infection and risk of leukaemia in the offspring | Nested Case-control | | Chlamydia trachomatis IgM | | ALL | |  |  | 8 | 0 | ∞ |
| Lehtinen | 2005 | Associations between three types of maternal bacterial infection and risk of leukaemia in the offspring | Nested Case-control | | Chlamydia trachomatis IgG | | ALL | |  |  | 1 | 0.7 | 1.4 |
| Lehtinen | 2005 | Associations between three types of maternal bacterial infection and risk of leukaemia in the offspring | Nested Case-control | | Chlamydia trachomatis IgM | | ALL | |  |  | 0.4 | 0.1 | 1.9 |
| Lehtinen | 2005 | Associations between three types of maternal bacterial infection and risk of leukaemia in the offspring | Nested Case-control | | Chlamydia trachomatis IgG | | ALL | |  |  | 1 | 0.8 | 1.4 |
| Lehtinen | 2005 | Associations between three types of maternal bacterial infection and risk of leukaemia in the offspring | Nested Case-control | | Chlamydia trachomatis IgM | | ALL | |  |  | 0.7 | 0.2 | 2.3 |
| Lehtinen | 2005 | Associations between three types of maternal bacterial infection and risk of leukaemia in the offspring | Nested Case-control | | Chlamydia trachomatis IgG | | non-ALL leukaemia | |  |  | 0.7 | 1.4 | 4.8 |
| Lehtinen | 2005 | Associations between three types of maternal bacterial infection and risk of leukaemia in the offspring | Nested Case-control | | Chlamydia trachomatis IgG | | non-ALL leukaemia | |  |  | 1.8 | 0.8 | 3.8 |
| Lehtinen | 2005 | Associations between three types of maternal bacterial infection and risk of leukaemia in the offspring | Nested Case-control | | Chlamydia trachomatis IgM | | non-ALL leukaemia | |  |  | 1 | 0.1 | 9.6 |
| Lehtinen | 2005 | Associations between three types of maternal bacterial infection and risk of leukaemia in the offspring | Nested Case-control | | Chlamydia trachomatis IgG | | non-ALL leukaemia | |  |  | 1.6 | 0.8 | 3.2 |
| Lehtinen | 2005 | Associations between three types of maternal bacterial infection and risk of leukaemia in the offspring | Nested Case-control | | Chlamydia trachomatis IgM | | non-ALL leukaemia | |  |  | 0.8 | 0.1 | 7.1 |
| Lehtinen | 2005 | Associations between three types of maternal bacterial infection and risk of leukaemia in the offspring | Nested Case-control | | Chlamydia trachomatis IgG | | total leukemias | | 402 | 1212 | 1.6 | 0.6 | 4.3 |
| Lehtinen | 2005 | Associations between three types of maternal bacterial infection and risk of leukaemia in the offspring | Nested Case-control | | Chlamydia trachomatis IgM | | total leukemias | | 402 | 1212 | 4 | 0.3 | 64 |
| Lehtinen | 2005 | Associations between three types of maternal bacterial infection and risk of leukaemia in the offspring | Nested Case-control | | Chlamydia trachomatis IgG | | total leukemias | | 402 | 1212 | 1.1 | 0.8 | 1.4 |
| Lehtinen | 2005 | Associations between three types of maternal bacterial infection and risk of leukaemia in the offspring | Nested Case-control | | Chlamydia trachomatis IgM | | total leukemias | | 402 | 1212 | 0.5 | 0.2 | 1.8 |
| Lehtinen | 2005 | Associations between three types of maternal bacterial infection and risk of leukaemia in the offspring | Nested Case-control | | Chlamydia trachomatis IgG | | total leukemias | | 402 | 1212 | 1.1 | 0.8 | 1.5 |
| Lehtinen | 2005 | Associations between three types of maternal bacterial infection and risk of leukaemia in the offspring | Nested Case-control | | Chlamydia trachomatis IgM | | total leukemias | | 402 | 1212 | 0.7 | 0.2 | 2 |
| Lehtinen | 2005 | Associations between three types of maternal bacterial infection and risk of leukaemia in the offspring | Nested Case-control | | Helicobacter pylori IgG | | ALL | |  |  | 2.2 | 0.8 | 6.1 |
| Lehtinen | 2005 | Associations between three types of maternal bacterial infection and risk of leukaemia in the offspring | Nested Case-control | | Helicobacter pylori IgG | | ALL | |  |  | 0.9 | 0.7 | 1.3 |
| Lehtinen | 2005 | Associations between three types of maternal bacterial infection and risk of leukaemia in the offspring |  | | Helicobacter pylori IgM | | ALL | |  |  | 0.8 | 0.3 | 2.1 |
| Lehtinen | 2005 | Associations between three types of maternal bacterial infection and risk of leukaemia in the offspring | Nested Case-control | | Helicobacter pylori IgG | | ALL | |  |  | 1 | 0.8 | 1.3 |
| Lehtinen | 2005 | Associations between three types of maternal bacterial infection and risk of leukaemia in the offspring | Nested Case-control | | Helicobacter pylori IgM | | ALL | |  |  | 0.8 | 0.3 | 1.9 |
| Lehtinen | 2005 | Associations between three types of maternal bacterial infection and risk of leukaemia in the offspring | Nested Case-control | | Helicobacter pylori IgG | | non-ALL leukaemia | |  |  | 7.6 | 0.8 | 74 |
| Lehtinen | 2005 | Associations between three types of maternal bacterial infection and risk of leukaemia in the offspring | Nested Case-control | | Helicobacter pylori IgM | | non-ALL leukaemia | |  |  | 8 | 0 | ∞ |
| Lehtinen | 2005 | Associations between three types of maternal bacterial infection and risk of leukaemia in the offspring | Nested Case-control | | Helicobacter pylori IgG | | non-ALL leukaemia | |  |  | 0.6 | 0.2 | 1.2 |
| Lehtinen | 2005 | Associations between three types of maternal bacterial infection and risk of leukaemia in the offspring | Nested Case-control | | Helicobacter pylori IgM | | non-ALL leukaemia | |  |  | 1.5 | 0.1 | 17 |
| Lehtinen | 2005 | Associations between three types of maternal bacterial infection and risk of leukaemia in the offspring | Nested Case-control | | Helicobacter pylori IgG | | non-ALL leukaemia | |  |  | 0.8 | 0.4 | 1.6 |
| Lehtinen | 2005 | Associations between three types of maternal bacterial infection and risk of leukaemia in the offspring | Nested Case-control | | Helicobacter pylori IgM | | non-ALL leukaemia | |  |  | 5.1 | 0.8 | 30 |
| Lehtinen | 2005 | Associations between three types of maternal bacterial infection and risk of leukaemia in the offspring | Nested Case-control | | Helicobacter pylori IgG | | total leukemias | | 402 | 1212 | 2.8 | 1.1 | 6.9 |
| Lehtinen | 2005 | Associations between three types of maternal bacterial infection and risk of leukaemia in the offspring | Nested Case-control | | Helicobacter pylori IgM | | total leukemias | | 402 | 1212 | 4 | 0.6 | 28 |
| Lehtinen | 2005 | Associations between three types of maternal bacterial infection and risk of leukaemia in the offspring | Nested Case-control | | Helicobacter pylori IgG | | total leukemias | | 402 | 1212 | 0.9 | 0.7 | 1.2 |
| Lehtinen | 2005 | Associations between three types of maternal bacterial infection and risk of leukaemia in the offspring | Nested Case-control | | Helicobacter pylori IgM | | total leukemias | | 402 | 1212 | 0.9 | 0.4 | 2.1 |
| Lehtinen | 2005 | Associations between three types of maternal bacterial infection and risk of leukaemia in the offspring | Nested Case-control | | Helicobacter pylori IgG | | total leukemias | | 402 | 1212 | 1 | 0.8 | 1.2 |
| Lehtinen | 2005 | Associations between three types of maternal bacterial infection and risk of leukaemia in the offspring | Nested Case-control | | Helicobacter pylori IgM | | total leukemias | | 402 | 1212 | 1.1 | 0.5 | 2.4 |
| Linos | 1998 | Reported influenza in pregnancy and childhood tumour | Hospital-based Case-control | | influenza | | brain tumours and neuroblastomas | | 94 | 210 | 3.15 | 1.13 | 8.77 |
| Linos | 1998 | Reported influenza in pregnancy and childhood tumour | Hospital-based Case-control | | influenza | | brain tumours and neuroblastomas | | 94 | 210 | 3.29 | 1.03 | 10.7 |
| Ma | 2021 | Association between exposure during pregnancy and the risk of developing  solid tumours in second children: Results from a Chinese matched  case-control study | Hospital-based Case-Control | | common cold | | second-child solid tumours | | 80 | 160 | 0.84 | 0.38 | 1.86 |
| McKinney | 1999 | Pre- and perinatal risk factors for childhood leukaemia and other malignancies: a Scottish case control study | Population-based Case-Control | | any infection | | total leukaemias | | 144 | 271 | 1.43 | 0.85 | 2.42 |
| McKinney | 1999 | Pre- and perinatal risk factors for childhood leukaemia and other malignancies: a Scottish case control study | Population-based Case-Control | | respiratory tract infection | | total leukemias | | 144 | 271 | 1.46 | 0.58 | 3.67 |
| McKinney | 1999 | Pre- and perinatal risk factors for childhood leukaemia and other malignancies: a Scottish case control study | Population-based Case-Control | | viral infection | | total leukemias | | 144 | 271 | 1.18 | 0.38 | 3.68 |
| McKinney | 1999 | Pre- and perinatal risk factors for childhood leukaemia and other malignancies: a Scottish case control study | Population-based Case-Control | | genitourinary tract infection | | total leukemias | | 144 | 271 | 1.36 | 0.66 | 2.83 |
| McKinney | 1999 | Pre- and perinatal risk factors for childhood leukaemia and other malignancies: a Scottish case control study | Population-based Case-Control | | fungal infection | | total leukemias | | 144 | 271 |  |  |  |
| McKinney | 1999 | Pre- and perinatal risk factors for childhood leukaemia and other malignancies: a Scottish case control study | Population-based Case-Control | | any infection | | ALL | | 124 | 236 | 1.44 | 0.81 | 2.55 |
| McKinney | 1999 | Pre- and perinatal risk factors for childhood leukaemia and other malignancies: a Scottish case control study | Population-based Case-Control | | respiratory tract infection | | ALL | | 124 | 236 | 1.64 | 0.6 | 4.46 |
| McKinney | 1999 | Pre- and perinatal risk factors for childhood leukaemia and other malignancies: a Scottish case control study | Population-based Case-Control | | Viral infection | | ALL | | 124 | 236 |  |  |  |
| McKinney | 1999 | Pre- and perinatal risk factors for childhood leukaemia and other malignancies: a Scottish case control study | Population-based Case-Control | | genitourinary tract infection | | ALL | | 124 | 236 | 1.18 | 0.5 | 2.79 |
| McKinney | 1999 | Pre- and perinatal risk factors for childhood leukaemia and other malignancies: a Scottish case control study | Population-based Case-Control | | fungal infection | | ALL | | 124 | 236 |  |  |  |
| McKinney | 1999 | Pre- and perinatal risk factors for childhood leukaemia and other malignancies: a Scottish case control study | Population-based Case-Control | | any infection | | lymphoma | | 45 | 82 | 0.82 | 0.28 | 2.41 |
| McKinney | 1999 | Pre- and perinatal risk factors for childhood leukaemia and other malignancies: a Scottish case control study | Population-based Case-Control | | respiratory | | lymphoma | | 45 | 82 |  |  |  |
| McKinney | 1999 | Pre- and perinatal risk factors for childhood leukaemia and other malignancies: a Scottish case control study | Population-based Case-Control | | viral infection | | lymphoma | | 45 | 82 |  |  |  |
| McKinney | 1999 | Pre- and perinatal risk factors for childhood leukaemia and other malignancies: a Scottish case control study | Population-based Case-Control | | genitourinary tract infection | | lymphoma | | 45 | 82 | 1.5 | 0.34 | 6.7 |
| McKinney | 1999 | Pre- and perinatal risk factors for childhood leukaemia and other malignancies: a Scottish case control study | Population-based Case-Control | | fungal infection | | lymphoma | | 45 | 82 |  |  |  |
| McKinney | 1999 | Pre- and perinatal risk factors for childhood leukaemia and other malignancies: a Scottish case control study | Population-based Case-Control | | any infection | | CNS tumours | | 75 | 133 | 0.64 | 0.3 | 1.34 |
| McKinney | 1999 | Pre- and perinatal risk factors for childhood leukaemia and other malignancies: a Scottish case control study | Population-based Case-Control | | respiratory tract infection | | CNS tumours | | 75 | 133 | 0.38 | 0.08 | 1.97 |
| McKinney | 1999 | Pre- and perinatal risk factors for childhood leukaemia and other malignancies: a Scottish case control study | Population-based Case-Control | | viral infection | | CNS tumours | | 75 | 133 |  |  |  |
| McKinney | 1999 | Pre- and perinatal risk factors for childhood leukaemia and other malignancies: a Scottish case control study | Population-based Case-Control | | genitourinary tract infection | | CNS tumours | | 75 | 133 | 0.91 | 0.38 | 2.18 |
| McKinney | 1999 | Pre- and perinatal risk factors for childhood leukaemia and other malignancies: a Scottish case control study | Population-based Case-Control | | fungal infection | | CNS tumours | | 75 | 133 |  |  |  |
| McKinney | 1999 | Pre- and perinatal risk factors for childhood leukaemia and other malignancies: a Scottish case control study | Population-based Case-Control | | any infection | | other solid tumours | | 126 | 230 | 1.81 | 1.07 | 3.06 |
| McKinney | 1999 | Pre- and perinatal risk factors for childhood leukaemia and other malignancies: a Scottish case control study | Population-based Case-Control | | respiratory infection | | other solid tumours | | 126 | 230 | 14.1 | 1.76 | 113.7 |
| McKinney | 1999 | Pre- and perinatal risk factors for childhood leukaemia and other malignancies: a Scottish case control study | Population-based Case-Control | | viral infection | | other solid tumours | | 126 | 230 |  |  |  |
| McKinney | 1999 | Pre- and perinatal risk factors for childhood leukaemia and other malignancies: a Scottish case control study | Population-based Case-Control | | genitourinary tract infection | | other solid tumours | | 126 | 230 | 2.2 | 0.95 | 5.08 |
| McKinney | 1999 | Pre- and perinatal risk factors for childhood leukaemia and other malignancies: a Scottish case control study | Population-based Case-Control | | fungal infection | | other solid tumours | | 126 | 230 |  |  |  |
| Naumburg | 2002 | Perinatal Exposure To Infection and Risk of Childhood Leukaemia | Population-based Case-Control | | any infection | | childhood leukaemia (ALL and AML) | | 652 | 652 | 1.25 | 0.95 | 1.64 |
| Naumburg | 2002 | Perinatal Exposure To Infection and Risk of Childhood Leukaemia | Population-based Case-Control | | any infection | | ALL | | 578 | 578 | 1.22 | 0.92 | 1.63 |
| Naumburg | 2002 | Perinatal Exposure To Infection and Risk of Childhood Leukaemia | Population-based Case-Control | | any infection | | AML | |  |  | 1.44 | 0.62 | 3.38 |
| Naumburg | 2002 | Perinatal Exposure To Infection and Risk of Childhood Leukaemia | Population-based Case-Control | | lower genital tract infection | | childhood leukaemia (ALL and AML) | | 652 | 652 | 1.78 | 1.17 | 2.72 |
| Naumburg | 2002 | Perinatal Exposure To Infection and Risk of Childhood Leukaemia | Population-based Case-Control | | lower genital tract infection | | ALL | | 578 | 578 | 1.63 | 1.04 | 2.53 |
| Naumburg | 2002 | Perinatal Exposure To Infection and Risk of Childhood Leukaemia | Population-based Case-Control | | lower genital tract infection | | AML | |  |  | 4 | 0.85 | 18.8 |
| Naumburg | 2002 | Perinatal Exposure To Infection and Risk of Childhood Leukaemia | Population-based Case-Control | | Urinary tract infection | | childhood leukaemia (ALL and AML) | | 652 | 652 | 1.27 | 0.69 | 2.34 |
| Naumburg | 2002 | Perinatal Exposure To Infection and Risk of Childhood Leukaemia | Population-based Case-Control | | Urinary tract infection | | ALL | | 578 | 578 | 1.92 | 0.95 | 3.85 |
| Naumburg | 2002 | Perinatal Exposure To Infection and Risk of Childhood Leukaemia | Population-based Case-Control | | Urinary tract infection | | AML | |  |  |  |  |  |
| Naumburg | 2002 | Perinatal Exposure To Infection and Risk of Childhood Leukaemia | Population-based Case-Control | | other maternal infection | | childhood leukaemia (ALL and AML) | | 652 | 652 | 1.13 | 0.83 | 1.55 |
| Naumburg | 2002 | Perinatal Exposure To Infection and Risk of Childhood Leukaemia | Population-based Case-Control | | other maternal infection | | ALL | | 578 | 578 | 1.13 | 0.81 | 1.6 |
| Naumburg | 2002 | Perinatal Exposure To Infection and Risk of Childhood Leukaemia | Population-based Case-Control | | other maternal infection | | AML | |  |  | 1.14 | 0.41 | 3.15 |
| Oksuzyana | 2013 | Birth weight and other perinatal factors and childhood CNS tumours: A case–control study in California | Population-based Case-Control | | genital herpes | | CNS tumours | | 3308 | 3308 | 2.79 | 1.17 | 6.62 |
| Oksuzyana | 2013 | Birth weight and other perinatal factors and childhood CNS tumours: A case–control study in California | Population-based Case-Control | | infections (non-sexually transmitted) include -pyelonephritis, hepatitis B and rubella | | CNS tumours | | 3308 | 3308 | 0.28 | 0.09 | 0.85 |
| Olshan | 1993 | Risk Factors for Wilms Tumour | Population-based Case-Control | | venereal disease, or vaginal or urinary tract infections | | Wilm's Tumour | | 200 | 233 | 0.8 | 0.48 | 1.35 |
| Olshan | 1993 | Risk Factors for Wilms Tumour | Population-based Case-Control | | other infections | | Wilm's Tumour | | 200 | 233 | 2.16 | 1.11 | 4.19 |
| Roman | 1997 | Leukaemia and non-Hodgkin's lymphoma in children and young adults: are prenatal and neonatal factors important determinants of disease? | Case-Control | | viral infection | | total leukaemias | | 143 | 236 | 6 | 1.2 | 29.7 |
| Roman | 1997 | Leukaemia and non-Hodgkin's lymphoma in children and young adults: are prenatal and neonatal factors important determinants of disease? | Case-Control | | viral infection | | ALL | | 113 | 226 | 4 | 0.7 | 21.8 |
| Roman | 1997 | Leukaemia and non-Hodgkin's lymphoma in children and young adults: are prenatal and neonatal factors important determinants of disease? | Case-Control | | viral infection | | NHL | | 34 | 68 | 10.5 | 0.49 | 225.88 |
| Roman | 1997 | Leukaemia and non-Hodgkin's lymphoma in children and young adults: are prenatal and neonatal factors important determinants of disease? | Case-Control | | Influenza | | ALL | | 113 | 226 | 3.05 | 0.5 | 18.55 |
| Sepúlveda-Robles | 2025 | Early Infection Incidence and Risk of Acute Leukaemia Development Among Mexican Children | Case-Control | | Any infection | | Leukaemia | | 1455 | 1455 | 0.61 | 0.52 | 0.72 |
| Sepúlveda-Robles | 2025 | Early Infection Incidence and Risk of Acute Leukaemia Development Among Mexican Children | Case-Control | | Any infection | | ALL | | 1253 | 1455 | 0.59 | 0.5 | 0.7 |
| Sepúlveda-Robles | 2025 | Early Infection Incidence and Risk of Acute Leukaemia Development Among Mexican Children | Case-Control | | UTI | | Leukaemia | | 1455 | 1455 | 0.59 | 0.47 | 0.72 |
| Sepúlveda-Robles | 2025 | Early Infection Incidence and Risk of Acute Leukaemia Development Among Mexican Children | Case-Control | | UTI | | ALL | | 1253 | 1455 | 0.56 | 0.45 | 0.7 |
| Sepúlveda-Robles | 2025 | Early Infection Incidence and Risk of Acute Leukaemia Development Among Mexican Children | Case-Control | | Respiratory infection | | Leukaemia | | 1455 | 1455 | 0.58 | 0.49 | 0.69 |
| Sepúlveda-Robles | 2025 | Early Infection Incidence and Risk of Acute Leukaemia Development Among Mexican Children | | Case-Control | | Vaginal | | Leukaemia | 1455 | 1455 | 1.03 | 0.54 | 1.98 |
| Shu | 1995 | An exploratory analysis of risk factors for childhood malignant germ-cell tumours: report from the Childrens Cancer Group (Canada, United States) | population-based Case-Control | | any virus infection | | malignant germ cell tumours | | 105 | 639 | 0.4 | 0.1 | 1.3 |
| Shu | 1995 | An exploratory analysis of risk factors for childhood malignant germ-cell tumours: report from the Childrens Cancer Group (Canada, United States) | population-based Case-Control | | Urinary tract infection | | malignant germ cell tumours | | 105 | 639 | 3.1 | 1.5 | 6.6 |
| Shu | 1995 | An exploratory analysis of risk factors for childhood malignant germ-cell tumours: report from the Childrens Cancer Group (Canada, United States) | population-based Case-Control | | other infection | | malignant germ cell tumours | | 105 | 639 | 1.1 | 0.5 | 2.4 |
| Sirirungreung | 2024 | Maternal medically diagnosed infection and antibiotic | Cohort study | | medically diagnosed infection | | ALL | | 798 | 2265777 | 1.15 | 0.99 | 1.35 |
| Sirirungreung | 2024 | Maternal medically diagnosed infection and antibiotic | Cohort study | | viral infection | | ALL | | 798 | 2265777 | 1.07 | 0.86 | 1.34 |
| Sirirungreung | 2024 | Maternal medically diagnosed infection and antibiotic | Cohort study | | bacterial infection | | ALL | | 798 | 2265777 | 1.11 | 0.94 | 1.32 |
| Sirirungreung | 2024 | Maternal medically diagnosed infection and antibiotic | Cohort study | | respiratory infection | | ALL | | 798 | 2265777 | 1.14 | 0.95 | 1.36 |
| Sirirungreung | 2024 | Maternal medically diagnosed infection and antibiotic | Cohort study | | Urinary tract infection | | ALL | | 798 | 2265777 | 1.15 | 0.89 | 1.47 |
| Sirirungreung | 2024 | Maternal medically diagnosed infection and antibiotic | Cohort study | | medically diagnosed infection | | CNS tumours | | 389 | 2265777 | 0.98 | 0.79 | 1.22 |
| Sirirungreung | 2024 | Maternal medically diagnosed infection and antibiotic | Cohort study | | medically diagnosed infection | | hepatoblastoma | | 136 | 2265777 | 1.34 | 0.9 | 1.98 |
| Sirirungreung | 202 | Maternal medically diagnosed infection and antibiotic | Cohort study | | viral infection | | CNS tumours | | 389 | 2265777 | 1.13 | 0.84 | 1.52 |
| Sirirungreung | 2024 | Maternal medically diagnosed infection and antibiotic | Cohort study | | Urinary tract infection | | CNS tumours | | 389 | 2265777 | 1.02 | 0.72 | 1.46 |
| Sirirungreung | 2024 | Maternal medically diagnosed infection and antibiotic | Cohort study | | viral infection | | hepatoblastoma | | 136 | 2265777 | 1.54 | 0.92 | 2.56 |
| Sirirungreung | 2024 | Maternal medically diagnosed infection and antibiotic | Cohort study | | Urinary tract infection | | hepatoblastoma | | 136 | 2265777 | 0.84 | 0.41 | 1.71 |
| Stewart | 1957 | A Survey of Childhood Malignancies | Case-control | | all infection | | any cancer | | 619 | 619 | 2.69 | 0.71 | 10.18 |
| Stewart | 1958 | A Survey of Childhood Malignancies | Case-control | | genitourinary tract infection | | leukemias | | 619 | 619 | 2.69 | 0.71 | 10.18 |
| Stewart | 1958 | A Survey of Childhood Malignancies | Case-control | | genitourinary tract infection | | other cancer (not including leukaemia) | | 680 | 680 | 1.58 | 0.61 | 4.1 |
| Stewart | 1958 | A Survey of Childhood Malignancies | Case-Control | | herpes zoster | | leukaemia | | 619 | 619 | 2 | 0.18 | 22.15 |
| Stewart | 1958 | A Survey of Childhood Malignancies | Case-Control | | herpes zoster | | leukaemia | | 680 | 680 | 1 |  |  |
| Stewart | 1958 | A Survey of Childhood Malignancies | Case-Control | | mumps | | leukaemia | | 619 | 619 | 3 | 0.12 | 73.91 |
| Stewart | 1958 | A Survey of Childhood Malignancies | Case-Control | | mumps | | other cancer (not including leukaemia) | | 680 | 680 | 3 | 0.12 | 73.89 |
| Stewart | 1958 | A Survey of Childhood Malignancies | Case-Control | | infectious hepatitis | | leukaemia | | 619 | 619 | 3 | 0.12 | 73.91 |
| Stewart | 1958 | A Survey of Childhood Malignancies | Case-Control | | infectious hepatitis | | other cancer (not including leukaemia) | | 680 | 680 | 3 | 1.22 | 73.89 |
| Stolt | 2004 | Maternal Human Polyomavirus Infection and Risk of Neuroblastoma in the Child | Case-Control | | BKV IgG positive | | neuroblastoma | | 115 | 918 | 0.8 | 0.5 | 1.3 |
| Stolt | 2004 | Maternal Human Polyomavirus Infection and Risk of Neuroblastoma in the Child | Case-Control | | BKV IgM positive | | neuroblastoma | | 115 | 918 | 0.6 | 0.2 | 1.9 |
| Stolt | 2004 | Maternal Human Polyomavirus Infection and Risk of Neuroblastoma in the Child | Case-Control | | JCV IgG positive | | neuroblastoma | | 115 | 918 | 0.9 | 0.6 | 1.4 |
| Stolt | 2004 | Maternal Human Polyomavirus Infection and Risk of Neuroblastoma in the Child | Case-Control | | JCV IgM positive | | neuroblastoma | | 115 | 918 | 0.9 | 0.4 | 1.9 |
| Stolt | 2004 | Maternal Human Polyomavirus Infection and Risk of Neuroblastoma in the Child | Case-Control | | BKV IgG positive | | neuroblastoma diagnosed before 1 year of age | | 115 | 918 | 0.6 | 0.3 | 1.3 |
| Stolt | 2004 | Maternal Human Polyomavirus Infection and Risk of Neuroblastoma in the Child | Case-Control | | BKV IgM positive | | neuroblastoma diagnosed before 1 year of age | | 115 | 918 | 0 | 0 | 1.7 |
| Stolt | 2004 | Maternal Human Polyomavirus Infection and Risk of Neuroblastoma in the Child | Case-Control | | JCV IgG positive | | neuroblastoma diagnosed before 1 year of age | | 115 | 918 | 0.7 | 0.3 | 1.3 |
| Stolt | 2004 | Maternal Human Polyomavirus Infection and Risk of Neuroblastoma in the Child | Case-Control | | JCV IgM positive | | neuroblastoma diagnosed before 1 year of age | | 115 | 918 | 0.7 | 0.2 | 2.2 |
| Swerdlow | 1982 | Prenatal factors in the aetiology of testicular cancer:  an epidemiological study of childhood testicular  cancer deaths in Great Britain, 1953-73 | population-based? Case-Control | | cystitis | | testicular cancer | | 87 | 10128 | 4.02 |  |  |
| Swerdlow | 1982 | Prenatal factors in the aetiology of testicular cancer:  an epidemiological study of childhood testicular  cancer deaths in Great Britain, 1953-74 | population-based? Case-Control | | renal infections | | testicular cancer | | 87 | 10128 | 1.48 |  |  |
| Swerdlow | 1982 | Prenatal factors in the aetiology of testicular cancer:  an epidemiological study of childhood testicular  cancer deaths in Great Britain, 1953-75 | population-based? Case-Control | | Any non-venereal urinary tract infection | | testicular cancer | | 87 | 10128 | 2.44 | 0.98 | 6.07 |
| Swerdlow | 1982 | Prenatal factors in the aetiology of testicular cancer:  an epidemiological study of childhood testicular  cancer deaths in Great Britain, 1953-76 | population-based? Case-Control | | tuberculosis | | testicular cancer | | 87 | 10128 | 12.52 |  |  |
| Tedeschi | 2009 | No Risk of Maternal EBV Infection for Childhood Leukaemia | Nested-Case Control | | EBV VCA IgM | | ALL | |  |  | 0.9 | 0.5 | 1.8 |
| Tedeschi | 2009 | No Risk of Maternal EBV Infection for Childhood Leukaemia | Nested-Case Control | | EBV EA IgG | | ALL | |  |  | 1 | 0.8 | 1.2 |
| Tedeschi | 2009 | No Risk of Maternal EBV Infection for Childhood Leukaemia | Nested-Case Control | | EBV ZEBRA IgG | | ALL | |  |  | 1.1 | 0.7 | 1.5 |
| Tedeschi | 2009 | No Risk of Maternal EBV Infection for Childhood Leukaemia | Nested-Case Control | | EBV ZEBRA IgG or EA IgG | | ALL | |  |  | 1 | 0.8 | 1.2 |
| Tedeschi | 2009 | No Risk of Maternal EBV Infection for Childhood Leukaemia | Nested-Case Control | | EBV ZEBRA IgG and EA IgG | | ALL | |  |  | 0.9 | 0.6 | 1.4 |
| Tedeschi | 2009 | No Risk of Maternal EBV Infection for Childhood Leukaemia | Nested-Case Control | | EBV VCA IgM | | non-ALL leukaemia | |  |  | 1 | 0.3 | 4.1 |
| Tedeschi | 2009 | No Risk of Maternal EBV Infection for Childhood Leukaemia | Nested-Case Control | | EBV EA IgG | | non-ALL leukaemia | |  |  | 1.2 | 0.8 | 1.9 |
| Tedeschi | 2009 | No Risk of Maternal EBV Infection for Childhood Leukaemia | Nested-Case Control | | EBV ZEBRA IgG | | non-ALL leukaemia | |  |  | 1.1 | 0.6 | 2.3 |
| Tedeschi | 2009 | No Risk of Maternal EBV Infection for Childhood Leukaemia | Nested-Case Control | | EBV ZEBRA IgG or EA IgG | | non-ALL leukaemia | |  |  | 1.3 | 0.9 | 1.9 |
| Tedeschi | 2009 | No Risk of Maternal EBV Infection for Childhood Leukaemia | Nested-Case Control | | EBV ZEBRA IgG and EA IgG | | non-ALL leukaemia | |  |  | 1 | 0.4 | 2.6 |
| Tedeschi | 2009 | No Risk of Maternal EBV Infection for Childhood Leukaemia | Nested-Case Control | | EBV EA IgG | | ALL | |  |  | 1.4 | 0.6 | 3.4 |
| Tedeschi | 2009 | No Risk of Maternal EBV Infection for Childhood Leukaemia | Nested-Case Control | | EBV EA IgG | | ALL | |  |  | 1.2 | 0.6 | 2.4 |
| Tedeschi | 2009 | No Risk of Maternal EBV Infection for Childhood Leukaemia | Nested-Case Control | | EBV EA IgG | | ALL | |  |  | 0.9 | 0.7 | 1.2 |
| Tedeschi | 2009 | No Risk of Maternal EBV Infection for Childhood Leukaemia | Nested-Case Control | | EBV EA IgG | | ALL | |  |  | 0.9 | 0.6 | 1.3 |
| Tedeschi | 2009 | No Risk of Maternal EBV Infection for Childhood Leukaemia | Nested-Case Control | | EBV EA IgG | | ALL | |  |  | 1 | 0.8 | 1.2 |
| Tedeschi | 2009 | No Risk of Maternal EBV Infection for Childhood Leukaemia | Nested-Case Control | | EBV Zebra IgG | | ALL | |  |  | 1.7 | 0.4 | 8.1 |
| Tedeschi | 2009 | No Risk of Maternal EBV Infection for Childhood Leukaemia | Nested-Case Control | | EBV Zebra IgG | | ALL | |  |  | 1.4 | 0.4 | 4.8 |
| Tedeschi | 2009 | No Risk of Maternal EBV Infection for Childhood Leukaemia | Nested-Case Control | | EBV Zebra IgG | | ALL | |  |  | 1.1 | 0.7 | 1.6 |
| Tedeschi | 2009 | No Risk of Maternal EBV Infection for Childhood Leukaemia | Nested-Case Control | | EBV Zebra IgG | | ALL | |  |  | 0.9 | 0.5 | 1.6 |
| Tedeschi | 2009 | No Risk of Maternal EBV Infection for Childhood Leukaemia | Nested-Case Control | | EBV Zebra IgG | | ALL | |  |  | 1 | 0.7 | 1.5 |
| Tedeschi | 2009 | No Risk of Maternal EBV Infection for Childhood Leukaemia | Nested-Case Control | | EBV EA IgG | | non-ALL leukaemia | |  |  | 0.9 | 0.3 | 2.8 |
| Tedeschi | 2009 | No Risk of Maternal EBV Infection for Childhood Leukaemia | Nested-Case Control | | EBV EA IgG | | non-ALL leukaemia | |  |  | 1.1 | 0.4 | 2.9 |
| Tedeschi | 2009 | No Risk of Maternal EBV Infection for Childhood Leukaemia | Nested-Case Control | | EBV EA IgG | | non-ALL leukaemia | |  |  | 1 | 0.5 | 2.1 |
| Tedeschi | 2009 | No Risk of Maternal EBV Infection for Childhood Leukaemia | Nested-Case Control | | EBV EA IgG | | non-ALL leukaemia | |  |  | 1.8 | 0.9 | 3.6 |
| Tedeschi | 2009 | No Risk of Maternal EBV Infection for Childhood Leukaemia | Nested-Case Control | | EBV EA IgG | | non-ALL leukaemia | |  |  | 1.2 | 0.8 | 1.9 |
| Tedeschi | 2009 | No Risk of Maternal EBV Infection for Childhood Leukaemia | Nested-Case Control | | EBV Zebra IgG | | non-ALL leukaemia | |  |  | 0.9 | 0.1 | 6.2 |
| Tedeschi | 2009 | No Risk of Maternal EBV Infection for Childhood Leukaemia | Nested-Case Control | | EBV Zebra IgG | | non-ALL leukaemia | |  |  | 3.9 | 0.6 | 24 |
| Tedeschi | 2009 | No Risk of Maternal EBV Infection for Childhood Leukaemia | Nested-Case Control | | EBV Zebra IgG | | non-ALL leukaemia | |  |  | 1.5 | 0.4 | 4.9 |
| Tedeschi | 2009 | No Risk of Maternal EBV Infection for Childhood Leukaemia | Nested-Case Control | | EBV Zebra IgG | | non-ALL leukaemia | |  |  | 0.5 | 0.1 | 1.8 |
| Tedeschi | 2009 | No Risk of Maternal EBV Infection for Childhood Leukaemia | Nested-Case Control | | EBV Zebra IgG | | non-ALL leukaemia | |  |  | 1.1 | 0.6 | 2.3 |
| Tedeschi | 2009 | No Risk of Maternal EBV Infection for Childhood Leukaemia | Nested-Case Control | | EBV VCA IgM | | ALL and non-ALL | | 705 | 2105 | 0.89 | 0.49 | 1.62 |
| Vasconcelos | 2008 | Adenovirus detection in Guthrie cards from paediatric leukaemia cases and controls | population-based Case-control | | Adenovirus DNA | | ALL | | 89 | 100 | 0.37 | 0.01 | 9.21 |
| canteens-Moll | 1985 | Are maternal fertility problems related to childhood leukaemia? | population-based Case-control | | viral infection | | ALL | | 519 | 507 | 1.4 | 0.7 | 3.1 |
| Wang | 2019 | Maternal prenatal exposure to environmental factors and risk of childhood acute lymphocytic leukaemia: A hospital-based case-control study in China | hospital based Case-Control | | viral infectious disease (rubella, measles, cytomegalovirus, toxoplasma infection, but not including common viral infections like colds and influenza) | | ALL | | 345 | 345 | 0.72 | 0.19 | 2.75 |
| Wiemels | 2019 | Early infection with cytomegalovirus and risk of childhood haematological malignancies | population-based Cohort | | CMV | | haematological malignancies (ICD-9: 200–208) | | 2265 | 2779309 | 13.5 | 1.9 | 96 |
| Wiemels | 2019 | Early infection with cytomegalovirus and risk of childhood haematological malignancies | population-based Cohort | | CMV | | haematological malignancies (ICD-9: 200–208) | | 2265 | 2779309 | 13.5 | 1.9 | 96 |
| Wiemels | 2019 | Early infection with cytomegalovirus and risk of childhood haematological malignancies | population-based Cohort | | CMV | | haematological malignancies (ICD-9: 200–208) | | 2265 | 2779309 | 13.6 | 1.9 | 96.5 |

Abbreviations: AML, acute myeloid leukaemia; ALL; acute lymphoblastic leukaemia; CMV, cytomegalovirus; CNS, central nervous system; EBV, Epstein Barr virus; ES estimate; IgG, immunoglobulin G; IgM, immunoglobulin M; LCI, lower confidence interval; NHL, non-Hodgkin lymphoma; TTV, torque teno virus; TTMV, torque teno mini virus ; TTMDV, torque teno midi virus; UCI, upper confidence interval; UTI, urinary tract infection.
