# Supplementary material for: Maternal infection during pregnancy and the risk of childhood cancer: a systematic review and meta-analysis
Source: BMC Med. 2026 Jan 14;24:51. doi: 10.1186/s12916-026-04625-1 (PMC12849171; doi:10.1186/s12916-026-04625-1)
Supplement: Supplementary file 4 — Additional file 4: Table. S4: Studies excluded from systematic review and meta-analysis. [file 12916_2026_4625_MOESM4_ESM.docx]

**Additional file 4: Table. S4: Studies excluded from systematic review and meta-analysis**

| **Author** | **Title** | **Reason for exclusion** |
| --- | --- | --- |
| Aldestein, 1972 | Malignant Disease in Children whose Mothers had Chickenpox, Mumps, or Rubella in | case report |
| Behnaz, 2024 | Examining the Effect of Parental COVID‐19 Vaccination Prior to Birth and the Association Between COVID‐19 and Cancer in Children Under Six | maternal infection not in pregnancy |
| Birch, 1982 | Pre-natal factors in the origin of germ cell tumours of childhood | no control group |
| Bogdanovic, 2004 | Human herpes virus 6 or Epstein-Barr virus were not detected in Guthrie cards from children who later developed leukaemia | no exposure in cases and controls |
| Chan, 2014 | Vertically transmitted nasopharyngeal infection of the human papillomavirus: Does it play an aetiological role in nasopharyngeal cancer? | Review |
| Chang, 1989 | Maternal transmission of hepatitis B virus in childhood hepatocellular carcinoma | maternal infection not in pregnancy |
| Engels, 2004 | Poliovirus vaccination during pregnancy, maternal seroconversion to simian virus 40, and risk of childhood cancer | indirect measure of maternal infection |
| Gallant, 2023 | Associations between early-life and in utero infections and cytomegalovirus-positive acute lymphoblastic leukaemia in children | no control group |
| He, 2022 | Common maternal infections during pregnancy and childhood leukaemia in the offspring: findings from six international birth cohorts | overlap in study-population |
| Isa, 2004 | Human parvovirus B19 DNA is not detected in Guthrie cards from children who have developed acute lymphoblastic leukaemia | no exposure in cases and controls |
| Knox, 1980 | Childhood leukaemia and mother-foetus infection | maternal infection not in pregnancy |
| Knox, 1983 | Foetal infection, childhood leukaemia and cancer | indirect measure of maternal infection |
| Leppik, 2007 | In vivo and in vitro intragenomic rearrangement of torque teno viruses | no estimates |
| Magnani, 2014 | SETIL: Italian multicentric epidemiological case-control study on risk factors for childhood leukaemia, non-Hodgkin lymphoma and neuroblastoma: study population and prevalence of risk factors in Italy | prevalence study |
| Mackenzie, 1974 | Influenza infections during pregnancy: association with congenital malformations and with subsequent neoplasms in children, and potential hazards of live virus vaccines | case report |
| Priftakis, 2003 | Human polyomavirus DNA is not detected in Guthrie cards (dried blood spots) from children who developed acute lymphoblastic leukaemia | no exposure in cases and controls |
| Tedeschi, 2006 | Activation of Maternal Epstein-Barr Virus Infection and Risk of Acute Leukaemia in the Offspring | overlap in study-population |
