# Supplementary material for: Maternal infection during pregnancy and the risk of childhood cancer: a systematic review and meta-analysis
Source: BMC Med. 2026 Jan 14;24:51. doi: 10.1186/s12916-026-04625-1 (PMC12849171; doi:10.1186/s12916-026-04625-1)
Supplement: Supplementary file 5 — Additional file 5: Fig.S1: Publication bias for maternal infection during pregnancy and risk of cancer. Trim and fill analyses were conducted for meta-analyses with evidence of publication bias. Abbreviations: CI, confidence interval; Conf, confidence; Eff, effect; Err, error; logor, log odds ratio; MSE, mean square error; P, P-value; REML, restricted maximum likelihood; se; standard error; SND, standard normal deviate; Std, standard. [file 12916_2026_4625_MOESM5_ESM.pdf]

Maternal overall infection during pregnancy and risk of overall cancer

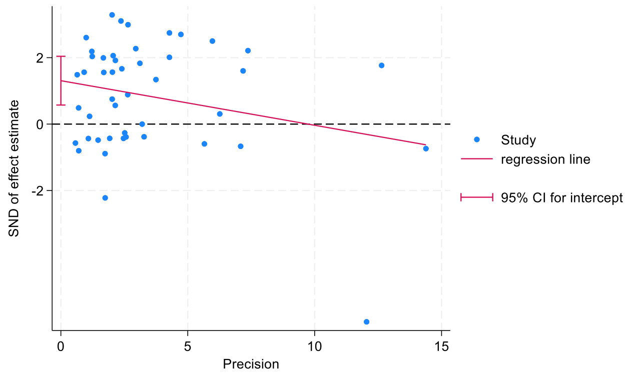

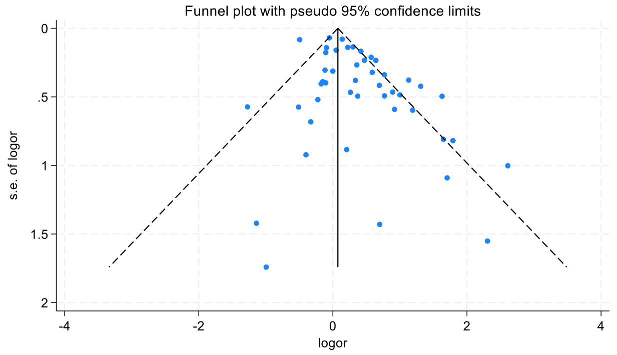

Number of studies = 46 Root MSE = 1.659

Std_Eff	Coefficient	Std. err.	t	P>|t|	[95% conf.	interval]
slope	-.1342607	.0787546	-1.70	0.095	-.2929802	.0244587
bias	1.307669	.3642178	3.59	0.001	.5736367	2.041702

Test of H0: no small-study effects P = 0.001

Trim and fill analysis of publication bias

Iteration	Number of studies =	50	
Model: Random-effects	observed =	46	
Method: REML	imputed =	4	
Pooling			
Model: Random-effects			
Method: REML			
Studies	Effect size	[95% conf. interval]	
Observed	1.36	1.17	1.59
Observed + Imputed	1.32	1.13	1.54

Infection and risk of leukaemia

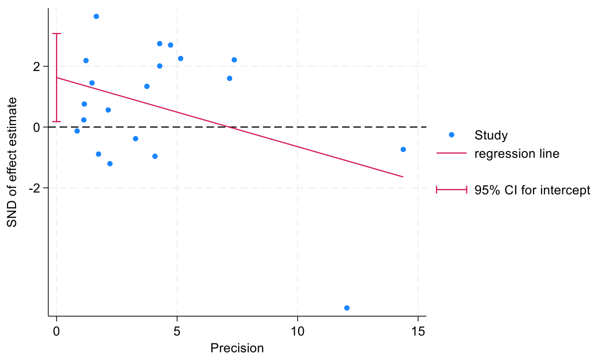

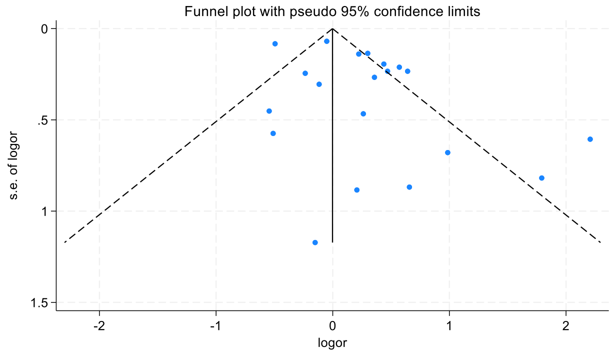

Number of studies = 20 Root MSE = 1.993

Std_Eff	Coefficient	Std. err.	t	P>|t|	[95% conf.	interval]
slope	-.227455	.1252034	-1.82	0.086	-.4904975	.0355876
bias	1.628585	.689802	2.36	0.030	.1793644	3.077805

Test of H0: no small-study effects P = 0.030

Trim and fill analysis of publication bias

Iteration	Number of studies =	23	
Model: Random-effects	observed =	20	
Method: REML	imputed =	3	
Pooling			
Model: Random-effects			
Method: REML			
Studies	Effect size	[95% conf. interval]	
Observed	1.27	1.01	1.60
Observed + Imputed	1.14	0.87	1.49

Infection and risk of acute lymphoblastic leukaemia

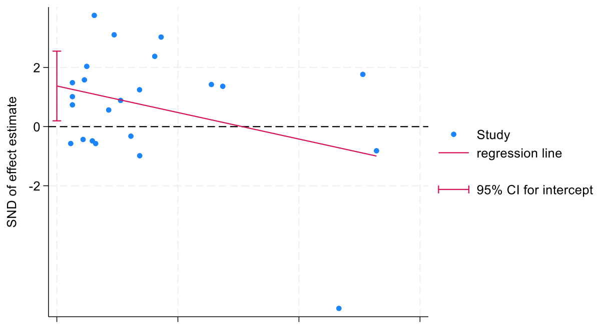

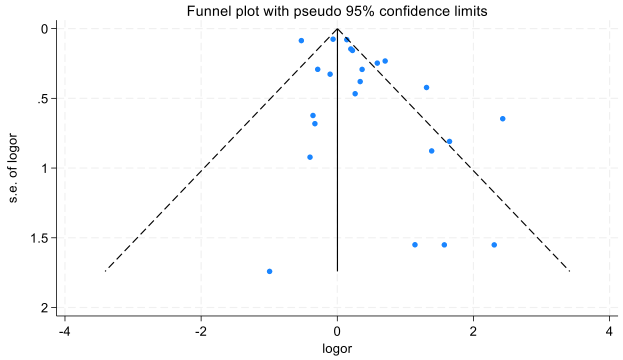

Number of studies = 23 Root MSE = 1.922

Std_Eff	Coefficient	Std. err.	t	P>|t|	[95% conf.	interval]
slope	-.179452	.1060909	-1.69	0.106	-.4000801	.0411761
bias	1.374173	.5661729	2.43	0.024	.1967523	2.551594

Test of H0: no small-study effects P = 0.024

Trim and fill analysis of publication bias

Iteration	Number of studies =	27	
Model: Random-effects	observed =	23	
Method: REML	imputed =	4	
Pooling			
Model: Random-effects			
Method: REML			
Studies	Effect size	[95% conf. interval]	
Observed	1.31	1.04	1.64
Observed + Imputed	1.20	0.89	1.62

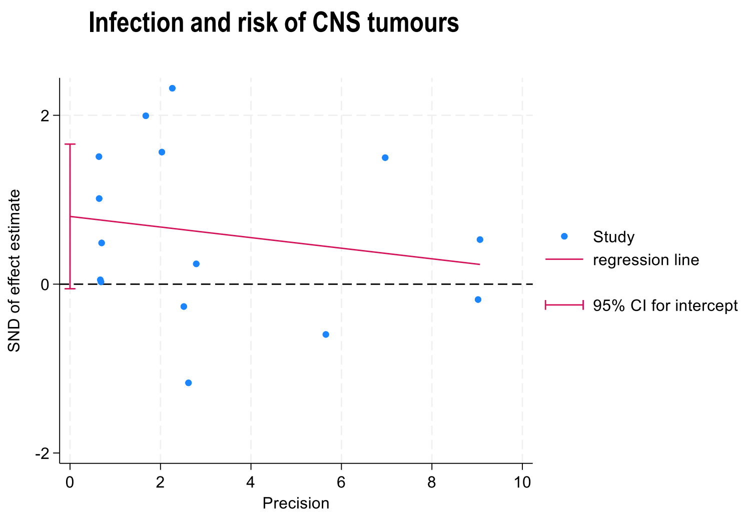

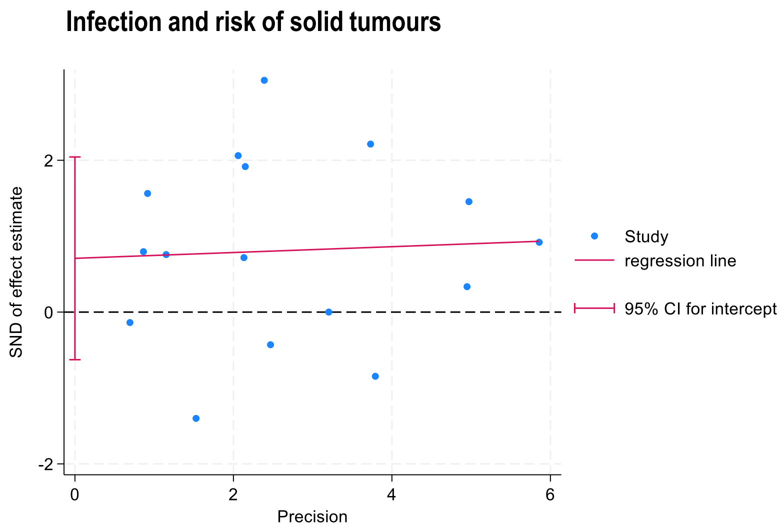

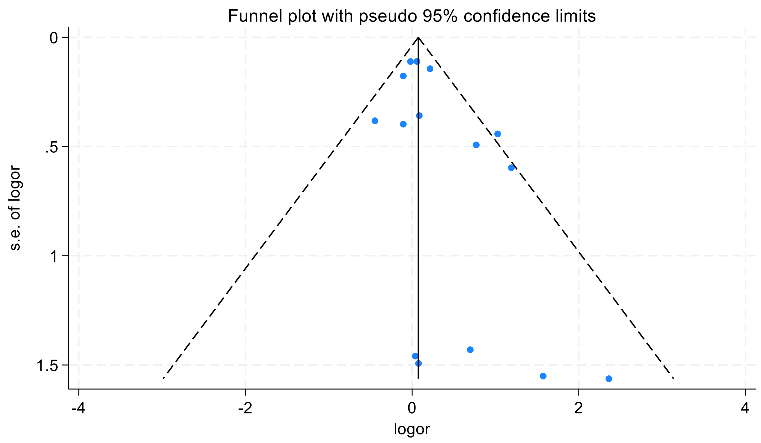

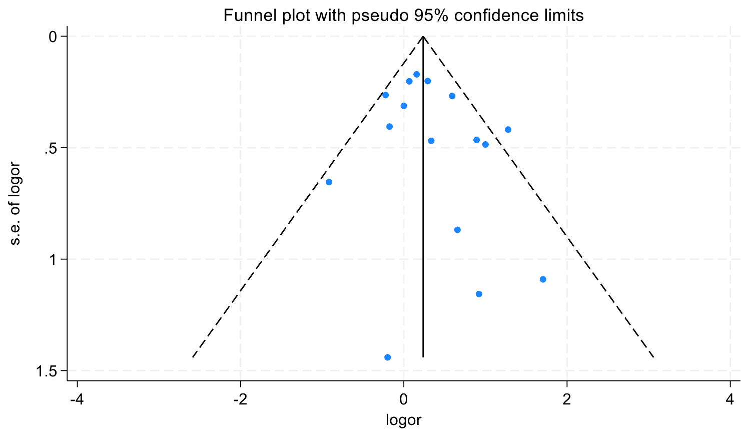

Number of studies = 15 Root MSE = 1.032

Std_Eff	Coefficient	Std. err.	t	P>|t|	[95% conf. interval]	
slope	-.062732	.091903	-0.68	0.507	-.2612763	.1358124
bias	.8022195	.396553	2.02	0.064	-.0544811	1.65892

Test of H0: no small-study effects P = 0.064

Number of studies = 16 Root MSE = 1.245

Std_Eff	Coefficient	Std. err.	t	P>|t|	[95% conf. interval]	
slope	.0383529	.2013619	0.19	0.852	-.3935255	.4702313
bias	.7082238	.622726	1.14	0.275	-.6273908	2.043838

Test of H0: no small-study effects P = 0.275

Viral infection and risk of childhood cancer

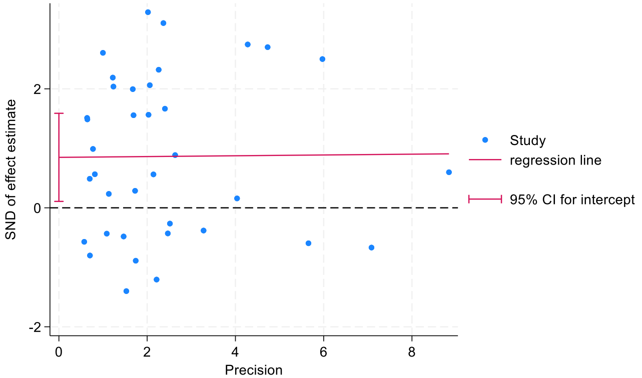

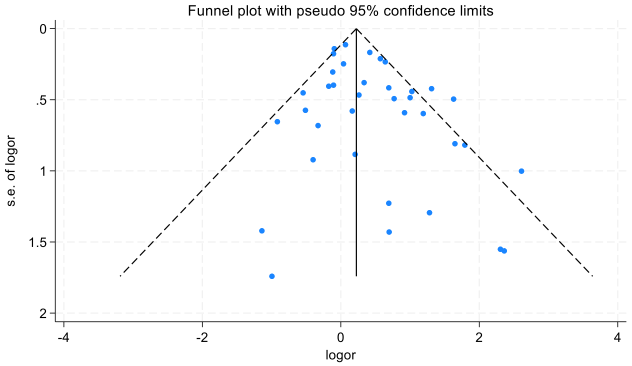

Number of studies = 37 Root MSE = 1.366

Std_Eff	Coefficient	Std. err.	t	P>|t|	[95% conf. interval]	
slope	.006692	.1190216	0.06	0.955	-.2349347	.2483186
bias	.8479754	.3647533	2.32	0.026	.1074869	1.588464

Test of H0: no small-study effects P = 0.026

Trim and fill analysis of publication bias

Iteration	Number of studies =	40	
Model: Random-effects	observed =	37	
Method: REML	imputed =	3	
Pooling			
Model: Random-effects			
Method: REML			
Studies	Effect size	[95% conf. interval]	
Observed	1.43	1.18	1.74
Observed + Imputed	1.40	1.14	1.71

Influenza infection and risk of childhood cancer

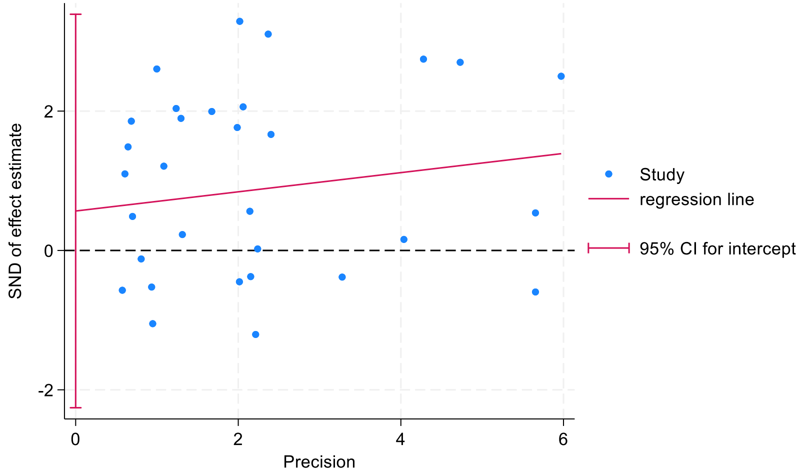

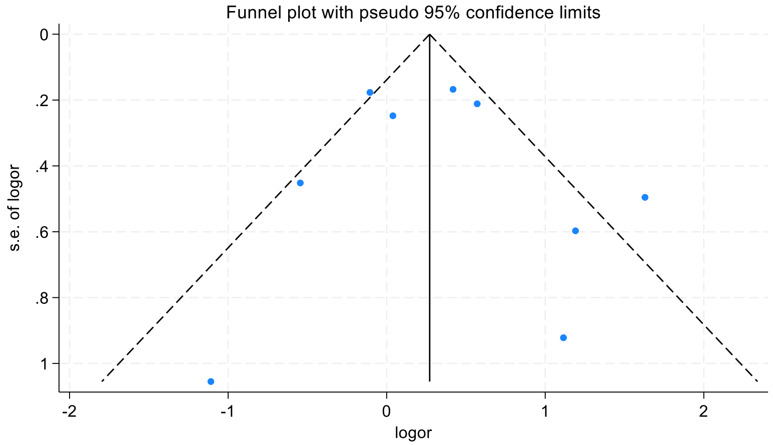

Number of studies = 9 Root MSE = 1.819

Std_Eff	Coefficient	Std. err.	t	P>|t|	[95% conf. interval]	
slope	.1377748	.3268072	0.42	0.686	-.6350014	.9105509
bias	.5661458	1.194148	0.47	0.650	-2.257565	3.389857

Test of H0: no small-study effects P = 0.650

Cytomegalovirus infection and risk of childhood cancer

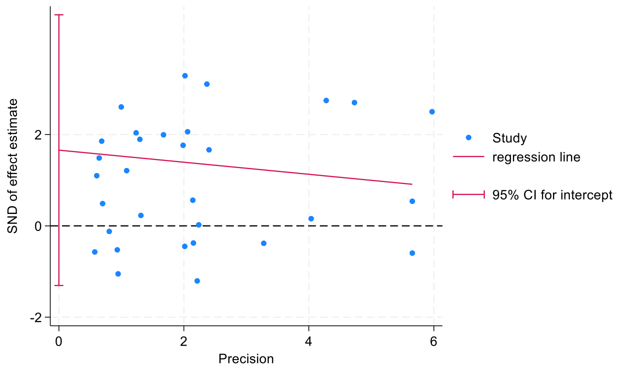

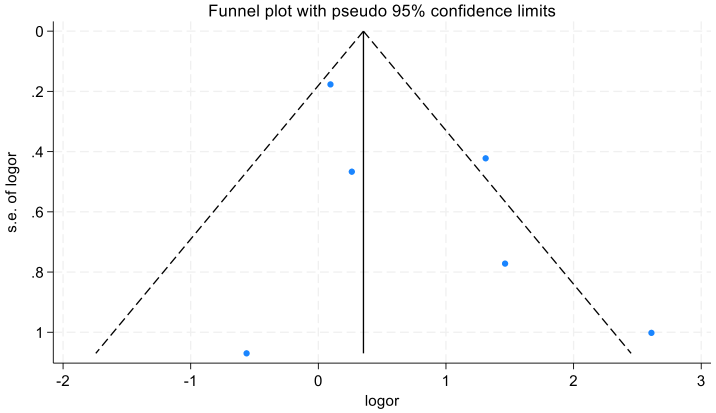

Number of studies = 6 Root MSE = 1.539

Std_Eff	Coefficient	Std. err.	t	P>|t|	[95% conf. interval]	
slope	-.1322436	.3867482	-0.34	0.750	-1.206029	.9415415
bias	1.658792	1.067725	1.55	0.195	-1.305689	4.623273

Test of H0: no small-study effects P = 0.195

Varicella infection and risk of childhood cancer

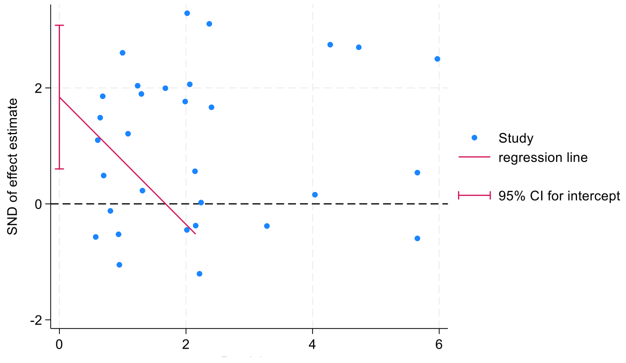

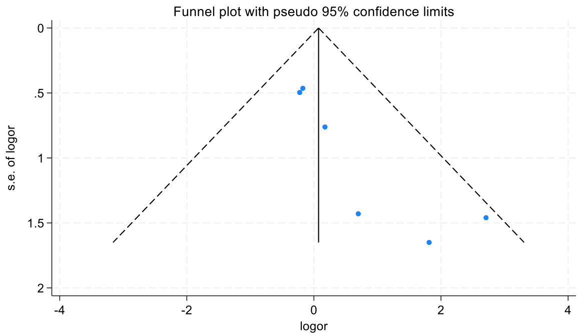

Number of studies = 6 Root MSE = .4982

Std_Eff	Coefficient	Std. err.	t	P>|t|	[95% conf. interval]	
slope	-1.09774	.3189106	-3.44	0.026	-1.983178	-.2123024
bias	1.841129	.4461335	4.13	0.015	.6024636	3.079794

Test of H0: no small-study effects P = 0.015

Trim and fill analysis of publication bias

Iteration	Number of studies = 9		
Model: Random-effects	observed = 6		
Method: REML	imputed = 3		
Pooling			
Model: Random-effects			
Method: REML			
Studies	Effect size	[95% conf. interval]	
Observed	1.10	0.60	2.00
Observed + Imputed	0.87	0.51	1.51

Genitourinary tract infection and risk of childhood cancer

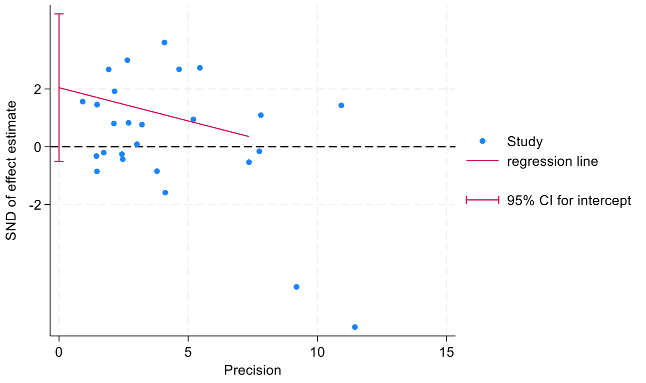

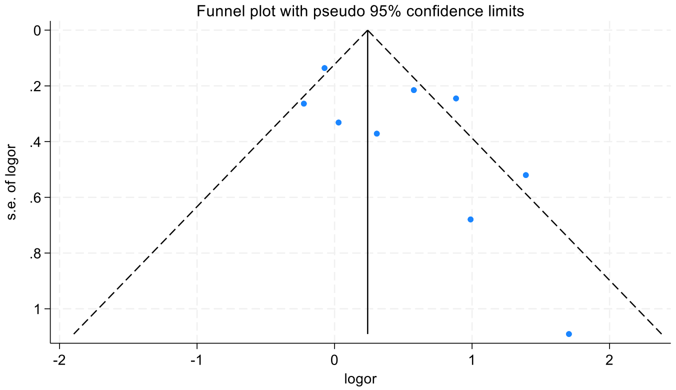

Number of studies = 9 Root MSE = 1.57

Std_Eff	Coefficient	Std. err.	t	P>|t|	[95% conf. interval]	
slope	-.2298926	.2844486	-0.81	0.446	-.9025067	.4427214
bias	2.043318	1.07998	1.89	0.100	-.5104287	4.597065

Test of H0: no small-study effects P = 0.100

Respiratory infection and risk of childhood cancer

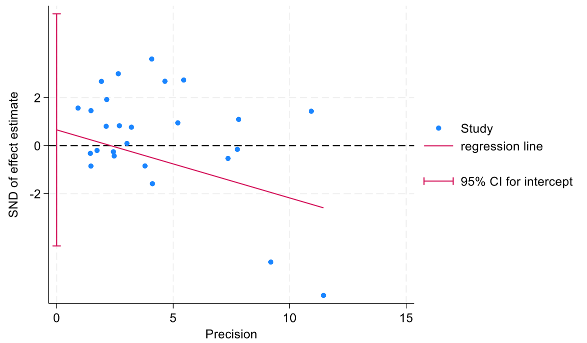

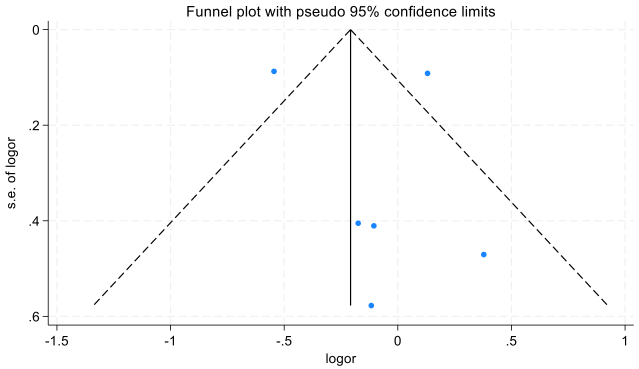

Number of studies = 6 Root MSE = 2.702

Std_Eff	Coefficient	Std. err.	t	P>|t|	[95% conf. interval]	
slope	-.2834529	.2594391	-1.09	0.336	-1.003771	.4368656
bias	.6558153	1.740607	0.38	0.725	-4.176885	5.488515

Test of H0: no small-study effects P = 0.725

Urinary tract infection and risk of childhood cancer

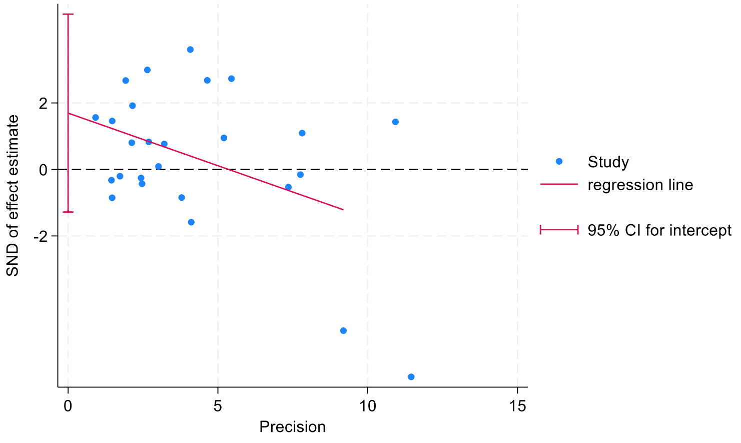

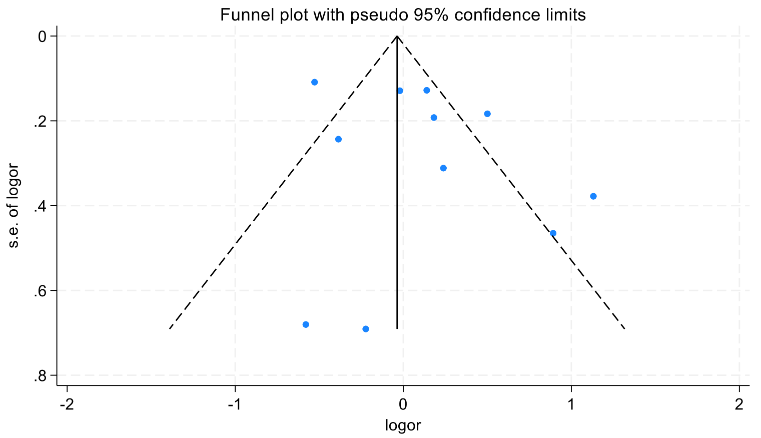

Number of studies = 11   Root MSE = 2.149

Std_Eff	Coefficient	Std. err.	t	P>|t|	[95% conf. interval]	
slope	-.3164344	.2495949	-1.27	0.237	-.8810572	.2481884
bias	1.69493	1.315302	1.29	0.230	-1.280489	4.670349

Test of H0: no small-study effects   P = 0.230

Sexually transmitted infection and risk of childhood cancer

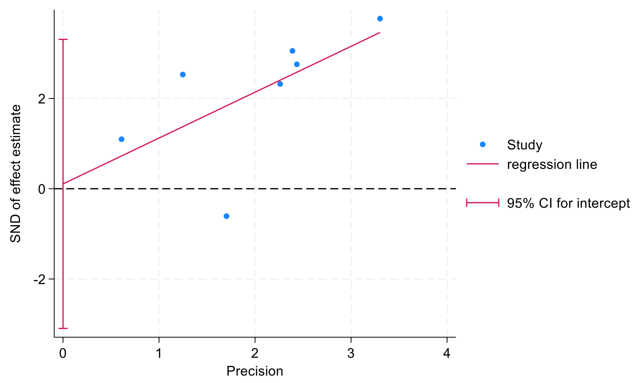

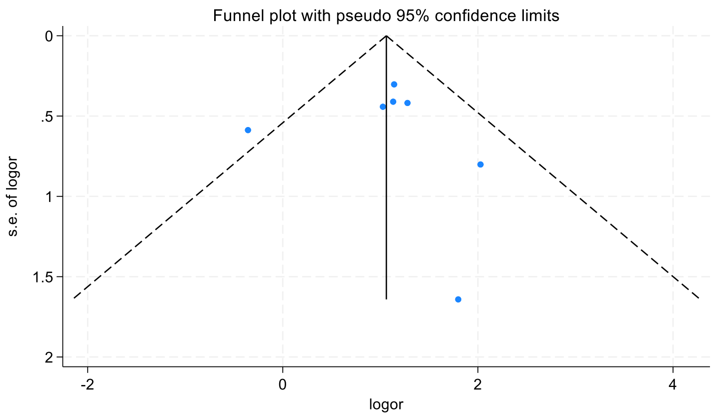

Number of studies = 7   Root MSE = 1.252

Std_Eff	Coefficient	Std. err.	t	P>|t|	[95% conf. interval]	
slope	1.015813	.5780553	1.76	0.139	-.4701255	2.501751
bias	.1066826	1.245338	0.09	0.935	-3.094559	3.307925

Test of H0: no small-study effects   P = 0.935

Viral infection and risk of leukaemia

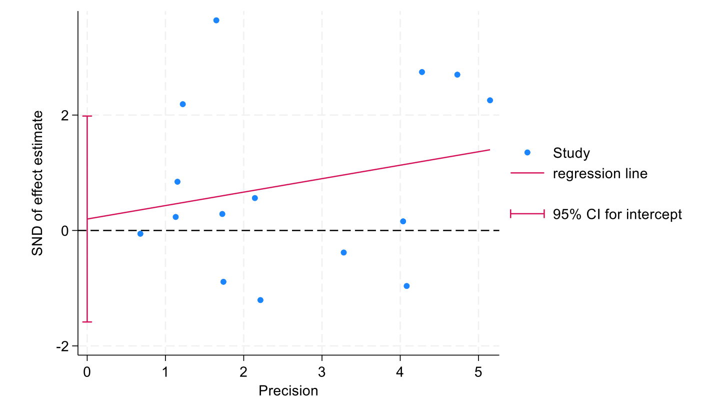

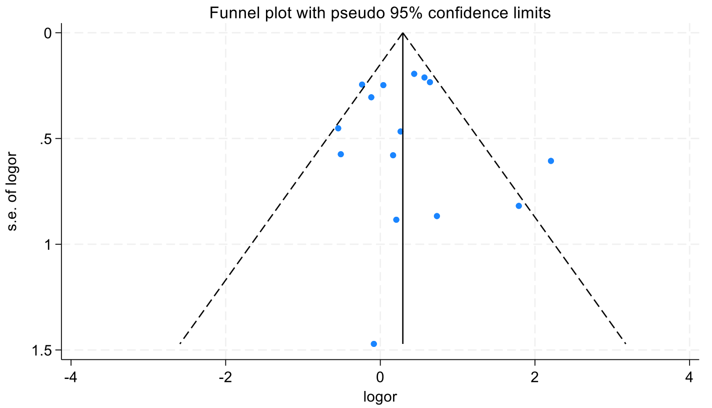

Number of studies = 15 Root MSE = 1.544

Std_Eff	Coefficient	Std. err.	t	P>|t|	[95% conf. interval]	
slope	.2331545	.2766358	0.84	0.415	-.3644807	.8307897
bias	.1986304	.825818	0.24	0.814	-1.585441	1.982702

Test of H0: no small-study effects P = 0.814

Genitourinary tract infection and risk of leukaemia

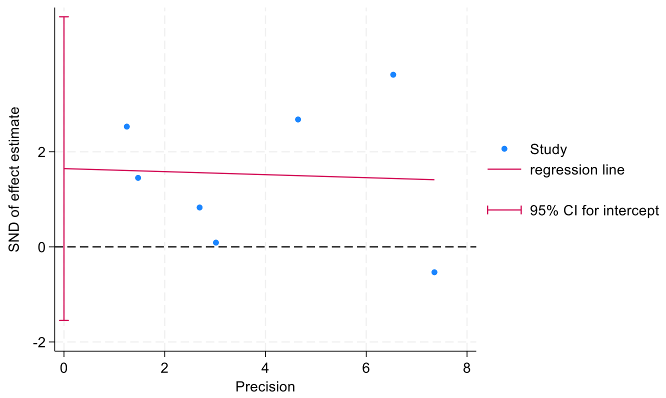

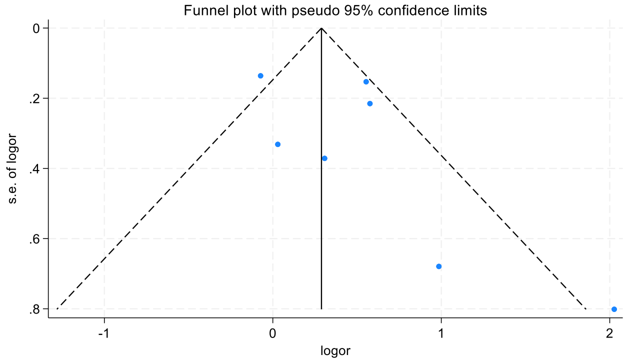

Number of studies = 7 Root MSE = 1.642

Std_Eff	Coefficient	Std. err.	t	P>|t|	[95% conf. interval]	
slope	-.0318003	.2792852	-0.11	0.914	-.7497257	.6861252
bias	1.64581	1.242079	1.33	0.242	-1.547055	4.838675

Test of H0: no small-study effects P = 0.242

Viral infection and risk of acute lymphoblastic leukaemia

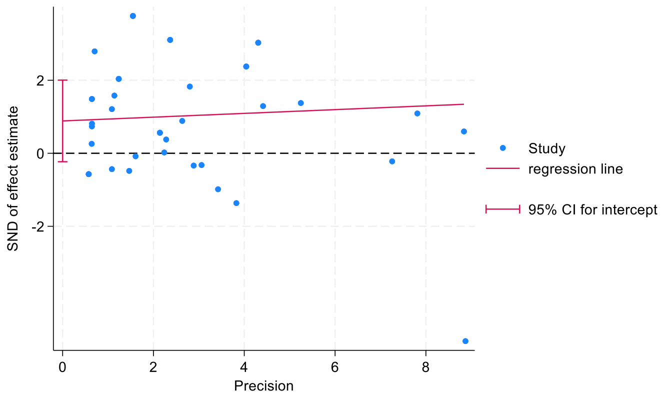

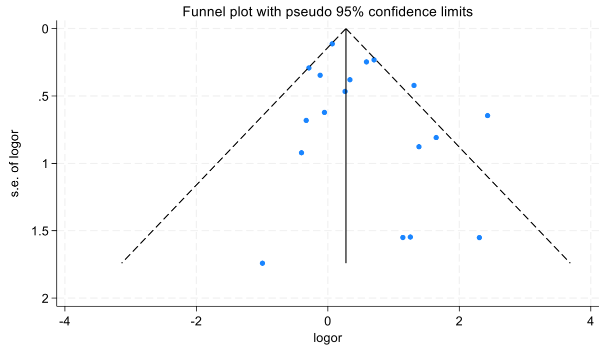

Number of studies = 18 Root MSE = 1.45

Std_Eff	Coefficient	Std. err.	t	P>|t|	[95% conf. interval]	
slope	.0516358	.1752054	0.29	0.772	-.319783	.4230547
bias	.8861263	.5271327	1.68	0.112	-.231345	2.003598

Test of H0: no small-study effects P = 0.112

Urinary tract infection and risk of acute lymphoblastic leukaemia

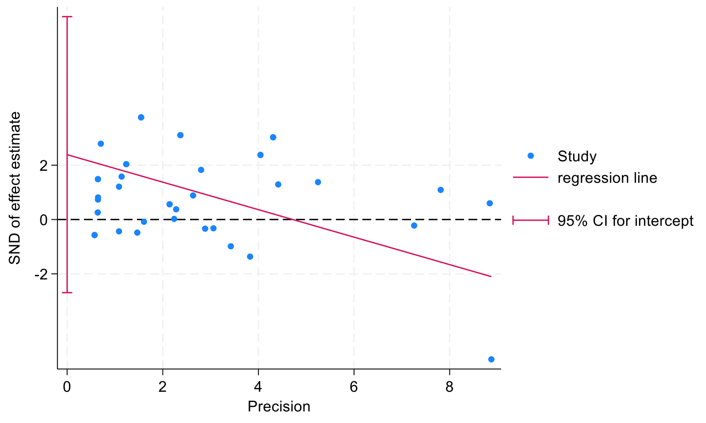

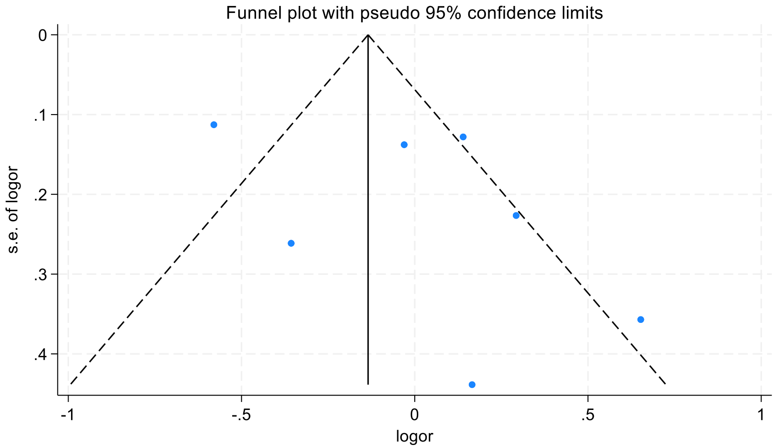

Number of studies = 7 Root MSE = 2.168

Std_Eff	Coefficient	Std. err.	t	P>|t|	[95% conf. interval]	
slope	-.5059589	.3377578	-1.50	0.194	-1.374193	.3622751
bias	2.387497	1.976009	1.21	0.281	-2.691996	7.46699

Test of H0: no small-study effects P = 0.281

Genitourinary tract infection and risk of solid tumours

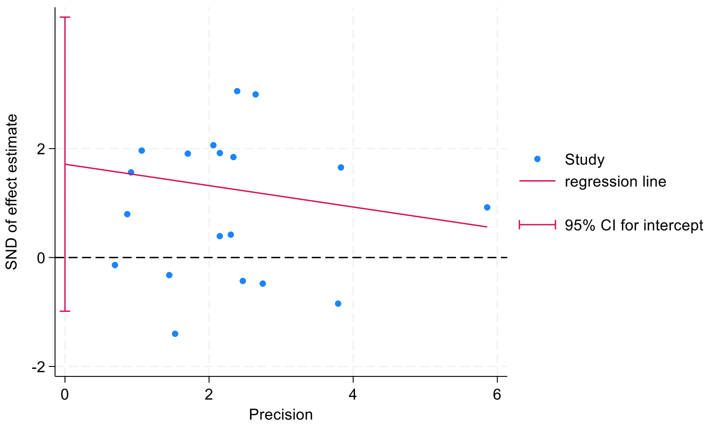

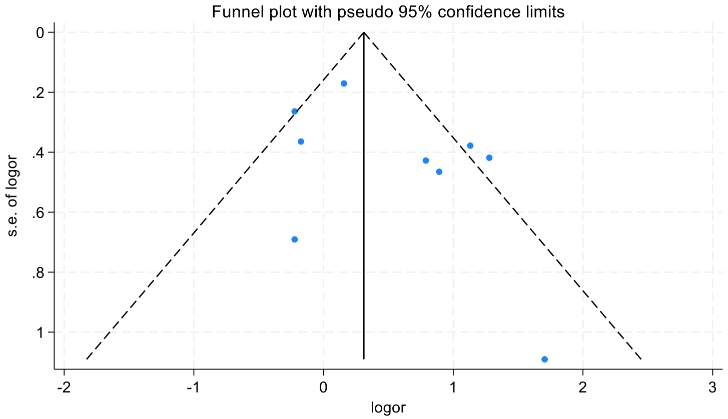

Number of studies = 9 Root MSE = 1.534

Std_Eff	Coefficient	Std. err.	t	P>|t|	[95% conf. interval]	
slope	-.1965062	.3782483	-0.52	0.619	-1.090921	.6979088
bias	1.712826	1.141494	1.50	0.177	-.986378	4.41203

Test of H0: no small-study effects P = 0.177

Viral infection and risk of solid tumours

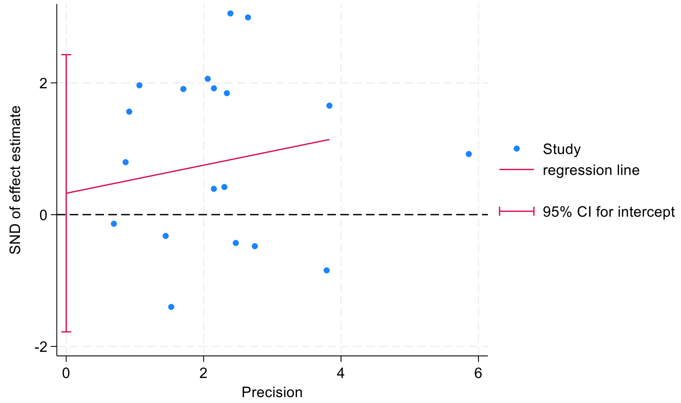

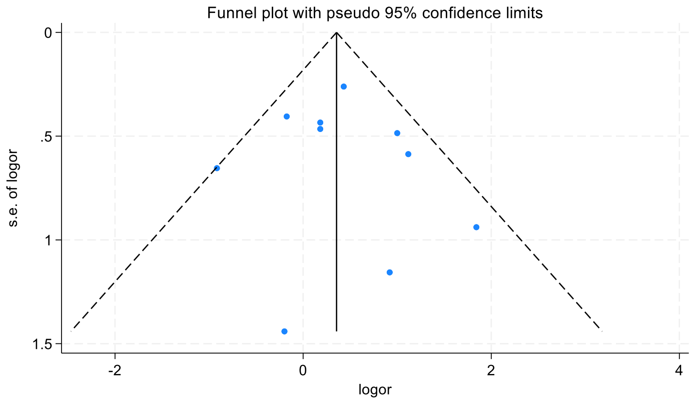

Number of studies = 10 Root MSE = 1.226

Std_Eff	Coefficient	Std. err.	t	P>|t|	[95% conf. interval]	
slope	.2130647	.4423772	0.48	0.643	-.8070589	1.233188
bias	.324602	.912298	0.36	0.731	-1.779161	2.428365

Test of H0: no small-study effects P = 0.731
