# Supplementary material for: Maternal infection during pregnancy and the risk of childhood cancer: a systematic review and meta-analysis
Source: BMC Med. 2026 Jan 14;24:51. doi: 10.1186/s12916-026-04625-1 (PMC12849171; doi:10.1186/s12916-026-04625-1)
Supplement: Supplementary file 6 — Additional file 6: Table. S5: Sensitivity analysis – leave-one-out meta-analysis. [file 12916_2026_4625_MOESM6_ESM.docx]

**Additional file 6: Table. S5: Sensitivity analyses – leave-one-out meta-analysis**

| **Model Description** | **ES (95% CI)** | **I- squared** | **P value** |
| --- | --- | --- | --- |
| **OMITTED STUDY: Overall maternal infection and risk of overall childhood cancer** |  |  |  |
| **Original effect estimate** | **1.36 1.17 1.59** | **71.20%** | **0.000** |
| Stewart, 1958 | 1.38 1.18 1.62 | 71.90% | 0.000 |
| Fedrick, 1972 | 1.33 1.14 1.54 | 70.00% | 0.000 |
| Bithell, 1973 | 1.36 1.16 1.59 | 71.10% | 0.000 |
| Heinonen, 1973 | 1.37 1.17 1.59 | 71.80% | 0.000 |
| Farwell, 1979 | 1.35 1.16 1.58 | 71.50% | 0.000 |
| Blot, 1980 | 1.35 1.16 1.58 | 71.50% | 0.000 |
| Swerdlow, 1982 | 1.35 1.15 1.57 | 71.30% | 0.000 |
| vanSteensel-Moll, 1985 | 1.36 1.16 1.59 | 71.80% | 0.000 |
| Fine, 1985 | 1.35 1.16 1.57 | 71.50% | 0.000 |
| Bunin, 1987 | 1.35 1.16 1.57 | 71.50% | 0.000 |
| Gardner, 1990 | 1.36 1.17 1.59 | 71.90% | 0.000 |
| Birch, 1990 | 1.36 1.17 1.59 | 71.80% | 0.000 |
| Olshan, 1993 | 1.34 1.15 1.57 | 71.10% | 0.000 |
| Shu, 1995 | 1.33 1.14 1.55 | 70.40% | 0.000 |
| Roman, 1997 | 1.34 1.15 1.56 | 71.10% | 0.000 |
| Linos, 1998 | 1.34 1.15 1.57 | 71.20% | 0.000 |
| Dockerty, 1999 | 1.36 1.16 1.59 | 71.80% | 0.000 |
| McKinney, 1999 | 1.36 1.16 1.59 | 71.70% | 0.000 |
| Fear, 2001 | 1.37 1.18 1.60 | 71.80% | 0.000 |
| Hamrik, 2001 | 1.39 1.18 1.62 | 71.70% | 0.000 |
| Naumburg, 2002 | 1.37 1.17 1.61 | 71.70% | 0.000 |
| Lehtinen, 2003 | 1.34 1.15 1.57 | 70.80% | 0.000 |
| Stolt, 2004 | 1.37 1.18 1.61 | 71.80% | 0.000 |
| Lehtinen, 2005 | 1.35 1.16 1.58 | 71.40% | 0.000 |
| Gustafsson, 2007 | 1.34 1.15 1.57 | 71.20% | 0.000 |
| Kwan, 2007 | 1.35 1.15 1.57 | 70.80% | 0.000 |
| Holl, 2008 | 1.34 1.15 1.57 | 71.20% | 0.000 |
| Vasconcelos, 2008 | 1.36 1.17 1.59 | 71.80% | 0.000 |
| Tedeschi, 2009 | 1.38 1.18 1.61 | 71.80% | 0.000 |
| Honkaniemi, 2010 | 1.35 1.16 1.57 | 71.50% | 0.000 |
| Heck, 2012 | 1.35 1.16 1.58 | 71.40% | 0.000 |
| Oksuzyana, 2013 | 1.39 1.19 1.62 | 70.80% | 0.000 |
| Kumar, 2014 | 1.38 1.18 1.61 | 71.80% | 0.000 |
| Heck, 2015 | 1.37 1.18 1.61 | 71.90% | 0.000 |
| Bzhalava, 2016 | 1.38 1.18 1.61 | 71.70% | 0.000 |
| Bogdanovic | 1.37 1.17 1.60 | 71.80% | 0.000 |
| Francis, 2017 | 1.33 1.14 1.55 | 70.20% | 0.000 |
| Wiemels, 2019 | 1.34 1.15 1.56 | 70.70% | 0.000 |
| Wang, 2019 | 1.37 1.17 1.60 | 71.80% | 0.000 |
| Ma, 2021 | 1.38 1.18 1.61 | 71.80% | 0.000 |
| Heck, 2021 | 1.39 1.18 1.63 | 71.60% | 0.000 |
| He, 2023 | 1.36 1.16 1.60 | 71.40% | 0.000 |
| Geris, 2023 | 1.36 1.17 1.60 | 71.90% | 0.000 |
| Sirirungreung, 2024 | 1.39 1.17 1.64 | 71.70% | 0.000 |
| Bonaventure, 2025 | 1.40 1.18 1.65 | 71.10% | 0.000 |
| Sepúlveda, 2025 | 1.38 1.21 1.59 | 56.60% | 0.000 |
| **OMITTED STUDY: Infection and risk of leukaemia** |  |  |  |
| **Original effect estimate** | **1.27 1.01 1.60** | 79.70% | 0.000 |
| Stewart, 1958 | 1.25 0.99 1.57 | 80.30% | 0.000 |
| Fedrick, 1972 | 1.20 0.96 1.49 | 77.60% | 0.000 |
| Bithell, 1973 | 1.25 0.98 1.59 | 79.60% | 0.000 |
| Fine, 1985 | 1.26 1.00 1.59 | 80.70% | 0.000 |
| Gardner, 1990 | 1.27 1.01 1.60 | 80.80% | 0.000 |
| Roman, 1997 | 1.23 0.98 1.55 | 79.70% | 0.000 |
| Dockerty, 1999 | 1.31 1.03 1.66 | 80.50% | 0.000 |
| McKinney, 1999 | 1.26 0.99 1.60 | 80.40% | 0.000 |
| Naumburg, 2001 | 1.28 0.99 1.63 | 80.20% | 0.000 |
| Lehtinen, 2003 | 1.23 0.97 1.55 | 79.00% | 0.000 |
| Lehtinen, 2005 | 1.23 0.98 1.58 | 79.90% | 0.000 |
| Kwan, 2007 | 1.23 0.98 1.56 | 79.10% | 0.000 |
| Tedeschi, 2009 | 1.30 1.02 1.65 | 80.80% | 0.000 |
| Kumar, 2014 | 1.27 1.01 1.61 | 80.80% | 0.000 |
| Bzhalava, 2016 | 1.30 1.03 1.64 | 80.60% | 0.000 |
| Heck, 2021 | 1.31 1.03 1.67 | 80.60% | 0.000 |
| He, 2023 | 1.27 0.99 1.62 | 79.60% | 0.000 |
| Geris, 2023 | 1.27 1.00 1.61 | 80.70% | 0.000 |
| Bonaventure, 2025 | 1.27 1.01 1.60 | 79.70% | 0.000 |
| Sepúlveda, 2025 | 1.34 1.10 1.63 | 62.00% | 0.000 |

| **Model Description** | **ES (95% CI)** | **I- squared** | **P value** |
| --- | --- | --- | --- |
| **OMMITED STUDY: Infection and risk of acute lymphoblastic leukaemia** |  |  |  |
| **Original effect estimate** | **1.31 1.04 1.64** | **77.80%** | **0.000** |
| Fedrick, 1972 | 1.23 0.99 1.53 | 75.30% | 0.000 |
| Heinonen, 1973 | 1.30 1.04 1.64 | 78.70% | 0.000 |
| Fine, 1985 | 1.30 1.03 1.63 | 78.60% | 0.000 |
| vanSteensel-Moll, 1985 | 1.31 1.03 1.65 | 78.70% | 0.000 |
| Roman, 1997 | 1.28 1.02 1.61 | 78.30% | 0.000 |
| McKinney, 1999 | 1.30 1.03 1.65 | 78.50% | 0.000 |
| Naumburg, 2002 | 1.33 1.04 1.69 | 78.40% | 0.000 |
| Lehtinen, 2003 | 1.28 1.01 1.61 | 77.60% | 0.000 |
| Lehtinen, 2005 | 1.33 1.05 1.68 | 78.80% | 0.000 |
| Gustafsson, 2007 | 1.27 1.02 1.60 | 77.90% | 0.000 |
| Kwan, 2007 | 1.23 1.00 1.59 | 76.60% | 0.001 |
| Vasconcelos, 2008 | 1.32 1.05 1.65 | 78.80% | 0.000 |
| Tedeschi, 2009 | 1.34 1.06 1.70 | 78.80% | 0.000 |
| Honkaniemi, 2010 | 1.29 1.03 1.62 | 78.40% | 0.000 |
| Bogdanovic, 2016 | 1.32 1.05 1.66 | 78.80% | 0.000 |
| Francis, 2017 | 1.24 0.99 1.55 | 76.60% | 0.000 |
| Wang,2019 | 1.33 1.05 1.67 | 78.80% | 0.000 |
| Heck, 2021 | 1.36 1.07 1.72 | 78.60% | 0.000 |
| He, 2023 | 1.32 1.03 1.69 | 78.40% | 0.000 |
| Geris, 2023 | 1.31 1.04 1.66 | 78.80% | 0.000 |
| Sirirungreung, 2024 | 1.35 1.04 1.76 | 78.00% | 0.000 |
| Bonaventure, 2025 | 1.38 1.06 1.81 | 78.60% | 0.000 |
| Sepúlveda, 2025 | 1.36 1.12 1.65 | 59.00% | 0.000 |
| **OMITTED STUDY: Infection and risk of CNS tumours and neuroblastomas** |  |  |  |
| **Original effect estimate** | **1.11 0.94 1.31** | **23.10%** | **0.198** |
| Fedrick, 1972 | 1.12 0.94 1.33 | 28.60% | 0.150 |
| Heinonen, 1973 | 1.13 0.94 1.33 | 28.60% | 0.150 |
| Farwell, 1979 | 1.09 0.93 1.27 | 19.60% | 0.240 |
| Fine, 1985 | 1.11 0.94 1.31 | 24.70% | 0.187 |
| Birch, 1990 | 1.11 0.94 1.32 | 27.80% | 0.157 |
| Linos, 1998 | 1.08 0.93 1.24 | 11.40% | 0.328 |
| McKinney, 1999 | 1.13 0.96 1.33 | 20.20% | 0.234 |
| Fear, 2001 | 1.10 0.94 1.28 | 19.00% | 0.246 |
| Hamrik, 2001 | 1.15 0.96 1.39 | 23.60% | 0.198 |
| Stolt, 2004 | 1.13 0.94 1.35 | 27.70% | 0.158 |
| Oksuzyana, 2013 | 1.06 0.94 1.20 | 3.60% | 0.411 |
| Heck, 2021 | 1.12 0.94 1.34 | 28.60% | 0.150 |
| He, 2023 | 1.09 0.90 1.32 | 23.70% | 0.197 |
| Sirirungreung, 2024 | 1.16 0.95 1.43 | 24.20% | 0.192 |
| Bonaventure, 2025 | 1.15 0.93 1.43 | 28.40% | 0.151 |
| **OMITTED STUDY: Infection and risk of solid tumours** |  |  |  |
| **Original effect estimate** | **1.32 1.05 1.66** | **36.70%** | **0.070** |
| Fedrick, 1972 | 1.32 1.04 1.66 | 40.00% | 0.055 |
| Heinonen, 1973 | 1.33 1.05 1.68 | 40.70% | 0.051 |
| Swerdlow, 1982 | 1.28 1.02 1.61 | 35.30% | 0.086 |
| Fine, 1985 | 1.32 1.04 1.67 | 40.30% | 0.053 |
| Bunin, 1987 | 1.30 1.04 1.63 | 36.00% | 0.081 |
| Olshan, 1993 | 1.39 1.11 1.75 | 31.20% | 0.120 |
| Shu, 1995 | 1.36 1.09 1.68 | 31.90% | 0.114 |
| McKinney, 1999 | 1.28 1.01 1.62 | 35.60% | 0.084 |
| Holl, 2008 | 1.28 1.02 1.60 | 33.80% | 0.098 |
| Heck, 2012 | 1.23 1.01 1.50 | 18.90% | 0.242 |
| Heck, 2015 | 1.36 1.07 1.74 | 39.30% | 0.059 |
| Heck, 2021 | 1.33 1.04 1.69 | 40.80% | 0.050 |
| Ma, 2021 | 1.43 1.09 1.86 | 38.10% | 0.067 |
| He, 2023 | 1.36 1.05 1.78 | 40.20% | 0.054 |
| Sirirungreung, 2024 | 1.33 1.03 1.73 | 40.70% | 0.051 |
| Bonaventure, 2025 | 1.37 1.06 1.77 | 38.80% | 0.062 |
| **OMITTED STUDY: Infection and risk of lymphoma** |  |  |  |
| **Original effect estimate** | **1.10 0.63 1.91** | **56.90%** | **0.041** |
| Fedrick, 1972 | 1.03 0.59 1.77 | 59.80% | 0.041 |
| Roman, 1997 | 1.03 0.60 1.76 | 58.80% | 0.046 |
| McKinney, 1999 | 1.17 0.61 2.25 | 63.80% | 0.026 |
| Heck, 2021 | 1.35 1.04 1.77 | 1.30% | 0.399 |
| He, 2023 | 1.10 0.48 2.52 | 65.40% | 0.021 |
| Bonaventure, 2025 | 1.07 0.46 2.51 | 59.20% | 0.044 |

| **Model Description** | **ES (95% CI)** | **I- squared** | **P value** | | |
| --- | --- | --- | --- | --- | --- |
| **OMITTED STUDY: Viral infection and risk of childhood cancer** |  |  |  | | |
| **Original effect estimate** | **1.43 1.18 1.74** | **52.30%** | **0.000** | | |
| Stewart, 1958 | 1.43 1.18 1.74 | 53.50% | 0.000 | | |
| Fedrick, 1972 | 1.38 1.14 1.66 | 48.00% | 0.001 | | |
| Bithell, 1973 | 1.44 1.17 1.76 | 52.70% | 0.000 | | |
| Heinonen, 1973 | 1.44 1.19 1.75 | 53.00% | 0.000 | | |
| Farwell, 1979 | 1.42 1.17 1.73 | 52.80% | 0.000 | | |
| Blot, 1980 | 1.42 1.17 1.73 | 52.80% | 0.000 | | |
| Fine, 1985 | 1.42 1.17 1.72 | 52.70% | 0.000 | | |
| vanSteensel-Moll, 1985 | 1.44 1.18 1.76 | 53.50% | 0.000 | | |
| Gardner, 1990 | 1.44 1.18 1.75 | 53.60% | 0.000 | | |
| Birch, 1990 | 1.43 1.18 1.74 | 53.50% | 0.000 | | |
| Shu, 1995 | 1.46 1.21 1.77 | 51.60% | 0.000 | | |
| Roman, 1997 | 1.41 1.16 1.70 | 51.20% | 0.000 | | |
| Linos, 1998 | 1.41 1.16 1.71 | 51.90% | 0.000 | | |
| Dockerty, 1999 | 1.47 1.21 1.79 | 51.70% | 0.000 | | |
| McKinney, 1999 | 1.44 1.19 1.76 | 53.60% | 0.000 | | |
| Fear, 2001 | 1.42 1.17 1.72 | 52.40% | 0.000 | | |
| Hamrik, 2001 | 1.48 1.21 1.81 | 51.40% | 0.000 | | |
| Lehtinen, 2003 | 1.41 1.16 1.72 | 53.20% | 0.000 | | |
| Stolt, 2004 | 1.46 1.20 1.78 | 51.60% | 0.000 | | |
| Gustafsson, 2007 | 1.41 1.16 1.71 | 51.80% | 0.000 | | |
| Kwan, 2007 | 1.42 1.16 1.73 | 53.30% | 0.000 | | |
| Vasconcelos, 2008 | 1.44 1.19 1.75 | 51.90% | 0.000 | | |
| Holl, 2008 | 1.41 1.16 1.71 | 52.80% | 0.000 | | |
| Tedeschi, 2009 | 1.47 1.20 1.79 | 52.50% | 0.000 | | |
| Honkaniemi, 2010 | 1.42 1.17 1.72 | 51.40% | 0.000 | | |
| Oksuzyana, 2013 | 1.40 1.16 1.70 | 52.30% | 0.000 | | |
| Heck, 2015 | 1.43 1.18 1.74 | 52.60% | 0.000 | | |
| Bzhalava, 2016 | 1.46 1.20 1.78 | 53.30% | 0.000 | | |
| Bogdanovic, 2016 | 1.45 1.19 1.76 | 49.10% | 0.100 | | |
| Francis, 2017 | 1.38 1.14 1.68 | 49.80% | 0.000 | | |
| Wiemels, 2019 | 1.40 1.16 1.69 | 53.20% | 0.000 | | |
| Wang, 2019 | 1.45 1.19 1.77 | 50.60% | 0.000 | | |
| Heck, 2021 | 1.48 1.21 1.81 | 53.00% | 0.000 | | |
| Ma, 2021 | 1.46 1.20 1.78 | 52.00% | 0.000 | | |
| Geris, 2023 | 1.44 1.18 1.76 | 53.60% | 0.000 | | |
| Sirirungreung, 2024 | 1.48 1.20 1.83 | 53.20% | 0.000 | | |
| Bonaventure, 2025 | 1.46 1.20 1.79 | 51.10% | 0.000 | | |
| **OMITTED STUDY: Bacterial infection and risk of childhood cancer** |  |  |  | | |
| **Original effect estimate** | **1.58 0.92 2.73** | **75.20%** | **0.007** | | |
| Heck, 2015 | 1.87 0.92 3.81 | 83.40% | 0.002 | | |
| Lehtinen, 2005 | 1.88 0.72 4.91 | 80.50% | 0.006 | | |
| Swerdlow, 1982 | 1.17 0.97 1.41 | 7.80% | 0.338 | | |
| Sirirungreung, 2024 | 2.22 0.84 5.85 | 75.90% | 0.016 | | |
| **OMITTED STUDY: Adenovirus infection and risk of childhood cancer** |  |  |  | | |
| **Original effect estimate** | **3.78 0.85 16.77** | **14.00%** | **0.313** | | |
| Gustafsson, 2007 | 2.12 0.08 53.46 | 50.10% | 0.157 | | |
| Vasconcelos, 2008 | 5.99 1.47 24.42 | 0.00% | 0.707 | | |
| Honkaniemi, 2010 | 2.18 0.19 24.84 | 47.20% | 0.169 | | |
| **OMITTED STUDY: Cytomegalovirus infection and risk of childhood cancer** |  |  |  | | |
| **Original effect estimate** | **2.10 1.02 4.34** | **67.10%** | **0.010** | | |
| Fine, 1985 | 1.89 0.87 4.11 | 69.30% | 0.011 | | |
| Lehtinen, 2003 | 2.70 1.17 6.21 | 50.50% | 0.088 | | |
| Holl, 2008 | 2.41 1.10 5.25 | 72.30% | 0.006 | | |
| Francis, 2017 | 1.76 0.82 3.80 | 57.10% | 0.053 | | |
| Wiemels, 2019 | 1.71 0.89 3.29 | 60.00% | 0.040 | | |
| Geris, 2023 | 2.47 0.96 6.36 | 73.60% | 0.004 | | |
| **OMITTED STUDY: Epstein-Barr virus infection and risk of childhood cancer** |  |  |  | | |
| **Original effect estimate** | **1.44 0.88 2.36** | **51.80%** | **0.101** | | |
| Lehtinen, 2003 | 1.25 0.66 2.39 | 49.10% | 0.140 | | |
| Holl, 2008 | 1.26 0.74 2.15 | 54.80% | 0.110 | | |
| Tedeschi, 2009 | 1.77 1.14 2.76 | 18.30% | 0.294 | | |
| Francis, 2017 | 1.58 0.86 2.90 | 63.00% | 0.067 | | |
| **OMITTED STUDY: Influenza virus infection and risk of childhood cancer** |  |  |  | | |
| **Original effect estimate** | **1.40 0.97 2.03** | **66.50%** | **0.002** | | |
| Fedrick, 1972 | 1.24 0.89 1.72 | 56.60% | 0.024 | | |
| Bithell, 1973 | 1.40 0.88 2.23 | 69.30% | 0.002 | | |
| Fine, 1985 | 1.46 1.01 2.12 | 68.40% | 0.002 | | |
| Roman, 1997 | 1.36 0.93 2.00 | 69.60% | 0.002 | | |
| Linos, 1998 | 1.32 0.90 1.92 | 67.40% | 0.003 | | |
| Dockerty, 1999 | 1.53 1.05 2.23 | 65.80% | 0.005 | | |
| Hamrik, 2001 | 1.54 1.03 2.32 | 60.50% | 0.013 | | |
| Kwan, 2007 | 1.35 0.88 2.08 | 67.30% | 0.003 | | |
| Bonaventure, 2025 | 1.49 0.97 2.30 | 69.40% | 0.002 | | |
| **Model Description** | **ES (95% CI)** | **I- squared** | | **P value** |  |
| **OMITTED STUDY: Rubella virus infection and risk of childhood cancer** |  |  | |  |  |
| **Original effect estimate** | **2.04 1.11 3.75** | **0.00%** | | **0.738** |  |
| Bithell, 1973 | 1.84 0.85 3.98 | 0.00% | | 0.519 |  |
| Blot, 1980 | 2.10 0.84 5.23 | 0.00% | | 0.438 |  |
| Fine, 1985 | 2.16 1.16 4.06 | 0.00% | | 0.766 |  |
| **OMITTED STUDY: Varicella zoster virus infection and risk of childhood cancer** |  |  | |  |  |
| **Original effect estimate** | **1.10 0.60 2.00** | **4.20%** | | **0.390** |  |
| Bithell, 1973 | 0.97 0.54 1.73 | 0.00% | | 0.768 |  |
| Blot, 1980 | 1.43 0.61 3.34 | 14.40% | | 0.322 |  |
| Fine, 1985 | 1.22 0.54 2.72 | 23.10% | | 0.267 |  |
| Birch, 1990 | 1.14 0.56 2.31 | 20.30% | | 0.285 |  |
| Roman, 1997 | 1.02 0.57 1.85 | 1.80% | | 0.396 |  |
| Bonaventure, 2025 | 1.45 0.60 3.50 | 15.80% | | 0.314 |  |
| **OMITTED STUDY: Genitourinary tract infection and risk of childhood cancer** |  |  | |  |  |
| **Original effect estimate** | **1.52 1.05 2.19** | **69.30%** | | **0.001** |  |
| Stewart, 1958 | 1.47 1.00 2.15 | 71.80% | | 0.001 |  |
| Swerdlow, 1982 | 1.38 0.97 1.98 | 66.70% | | 0.004 |  |
| Bunin, 1987 | 1.46 1.01 2.11 | 71.10% | | 0.001 |  |
| Olshan, 1993 | 1.69 1.13 2.52 | 69.00% | | 0.002 |  |
| McKinney, 1999 | 1.55 1.03 2.34 | 73.10% | | 0.000 |  |
| Naumburg, 2002 | 1.49 0.98 2.27 | 69.80% | | 0.002 |  |
| He, 2023 | 1.37 0.95 1.97 | 61.50% | | 0.011 |  |
| Bonaventure, 2025 | 1.67 1.13 2.47 | 58.70% | | 0.018 |  |
| Sepulveda, 2025 | 1.62 1.07 2.44 | 72.70% | | 0.001 |  |
| **OMITTED STUDY: Respiratory infection and risk of childhood cancer** |  |  | |  |  |
| **Original effect estimate** | **0.89 0.59 1.35** | **83.50%** | | **0.000** |  |
| McKinney, 1999 | 0.84 0.54 1.31 | 86.00% | | 0.000 |  |
| Heck, 2015 | 0.90 0.56 1.42 | 86.70% | | 0.000 |  |
| Ma, 2021 | 0.91 0.57 1.44 | 86.80% | | 0.000 |  |
| He, 2023 | 0.90 0.57 1.40 | 86.80% | | 0.000 |  |
| Sirirungreung, 2024 | 0.74 0.53 1.04 | 29.50% | | 0.225 |  |
| Sepulveda, 2025 | 1.18 0.95 1.32 | 0.00% | | 0.861 |  |
| **OMITTED STUDY: Urinary tract infection and risk of childhood cancer** |  |  | |  |  |
| **Original effect estimate** | **1.12 0.84 1.50** | **79.70%** | | **0.000** |  |
| Swerdlow, 1958 | 1.07 0.80 1.43 | 80.10% | | 0.000 |  |
| Shu, 1995 | 1.03 0.78 1.36 | 77.20% | | 0.000 |  |
| Dockerty, 1999 | 1.15 0.85 1.55 | 81.50% | | 0.000 |  |
| Hamrik, 2001 | 1.11 0.81 1.54 | 81.20% | | 0.000 |  |
| Naumburg, 2002 | 1.11 0.81 1.51 | 81.40% | | 0.000 |  |
| Kwan, 2007 | 1.19 0.87 1.62 | 80.90% | | 0.000 |  |
| Heck, 2015 | 1.14 0.84 1.53 | 81.70% | | 0.000 |  |
| He, 2023 | 1.06 0.79 1.43 | 77.40% | | 0.000 |  |
| Sirirungreung, 2024 | 1.12 0.80 1.57 | 80.80% | | 0.000 |  |
| Bonaventure, 2025 | 1.15 0.81 1.62 | 81.70% | | 0.000 |  |
| Sepulveda, 2025 | 1.22 0.96 1.54 | 57.30% | | 0.010 |  |
| **OMITTED STUDY: Sexually transmitted infection and risk of childhood cancer** |  |  | |  |  |
| **Original effect estimate** | **2.86 1.88 4.33** | **23.60%** | | **0.249** |  |
| Roman, 1997 | 2.81 1.80 4.40 | 34.60% | | 0.177 |  |
| Hamrik, 2001 | 2.79 1.65 4.71 | 36.00% | | 0.167 |  |
| Lehtinen, 2005 | 3.32 2.32 4.76 | 0.00% | | 0.918 |  |
| Kwan, 2007 | 2.69 1.78 4.07 | 21.00% | | 0.275 |  |
| Heck, 2012 | 2.70 1.62 4.50 | 33.60% | | 0.184 |  |
| Oksuzyana, 2013 | 2.86 1.70 4.79 | 36.30% | | 0.165 |  |
| He, 2023 | 2.76 1.58 4.82 | 35.50% | | 0.171 |  |
| **OMITTED STUDY: Viral Infections and risk of Leukaemia** |  |  | |  |  |
| **Original effect estimate** | **1.34 1.00 1.79** | **55.20%** | | **0.005** |  |
| Fedrick, 1972 | 1.24 0.97 1.58 | 37.90% | | 0.074 |  |
| Bithell, 1973 | 1.32 0.94 1.84 | 57.30% | | 0.004 |  |
| Heinonen, 1973 | 1.34 1.00 1.81 | 58.20% | | 0.003 |  |
| Fine, 1985 | 1.32 0.98 1.79 | 57.90% | | 0.004 |  |
| Gardner, 1990 | 1.34 0.99 1.81 | 58.20% | | 0.003 |  |
| Roman, 1997 | 1.28 0.96 1.70 | 53.10% | | 0.010 |  |
| Dockerty, 1999 | 1.41 1.05 1.89 | 52.90% | | 0.010 |  |
| McKinney, 1999 | 1.35 0.99 1.83 | 58.20% | | 0.003 |  |
| Lehtinen, 2003 | 1.28 0.93 1.76 | 54.40% | | 0.008 |  |
| Kwan, 2007 | 1.29 0.94 1.79 | 55.20% | | 0.006 |  |
| Tedeschi, 2009 | 1.39 1.02 1.90 | 55.50% | | 0.006 |  |
| Bzhalava, 2016 | 1.39 1.03 1.87 | 55.40% | | 0.006 |  |
| Heck, 2021 | 1.42 1.06 1.92 | 49.70% | | 0.018 |  |
| Geris, 2023 | 1.34 0.98 1.83 | 58.20% | | 0.003 |  |
| Bonaventure, 2025 | 1.38 1.00 1.91 | 56.60% | | 0.005 |  |

| **Model Description** | **ES (95% CI)** | **I- squared** | **P value** |
| --- | --- | --- | --- |
| **OMITTED STUDY: Genitourinary tract Infection and risk of Leukaemia** |  |  |  |
| **Original effect estimate** | **1.49 1.05 2.12** | **67.10%** | **0.006** |
| Stewart, 1958 | 1.44 1.00 2.07 | 70.80% | 0.004 |
| McKinney, 1999 | 1.53 1.02 2.27 | 72.60% | 0.003 |
| Naumburg, 2002 | 1.45 0.95 2.19 | 68.90% | 0.007 |
| Kwan, 2007 | 1.38 1.00 1.90 | 62.90% | 0.019 |
| He, 2023 | 1.45 0.95 2.22 | 64.00% | 0.016 |
| Bonaventure, 2025 | 1.68 1.27 2.22 | 24.70% | 0.249 |
| Sepulveda, 2025 | 1.60 1.07 2.38 | 71.50% | 0.004 |
| **OMITTED STUDY: Influenza and risk of Leukaemia** |  |  |  |
| **Original effect estimate** | **1.55 0.97 2.47** | **64.80%** | **0.009** |
| Fedrick, 1972 | 1.32 0.95 1.84 | 35.80% | 0.168 |
| Bithell, 1973 | 1.60 0.83 3.10 | 70.60% | 0.005 |
| Fine, 1985 | 1.59 0.98 2.58 | 70.00% | 0.005 |
| Roman, 1997 | 1.49 0.91 2.44 | 69.60% | 0.006 |
| Dockerty, 1999 | 1.78 1.12 2.81 | 59.80% | 0.029 |
| Kwan, 2007 | 1.53 0.82 2.88 | 68.90% | 0.007 |
| Bonaventure, 2025 | 1.74 0.98 3.08 | 65.30% | 0.013 |
| **OMITTED STUDY: Urinary tract infection and risk of Leukaemia** |  |  |  |
| **Original effect estimate** | **0.92 0.63 1.34** | **82.40%** | **0.000** |
| Dockerty, 1999 | 0.95 0.63 1.41 | 85.70% | 0.000 |
| Naumburg, 2002 | 0.87 0.57 1.32 | 84.90% | 0.000 |
| Kwan, 2007 | 0.97 0.62 1.52 | 85.50% | 0.000 |
| He, 2023 | 0.79 0.57 1.09 | 67.60% | 0.015 |
| Bonaventure, 2025 | 0.90 0.53 1.51 | 84.80% | 0.000 |
| Sepulveda, 2025 | 1.05 0.74 1.48 | 62.70% | 0.030 |
| **OMITTED STUDY: Adenovirus infection and risk of ALL** |  |  |  |
| **Original effect estimate** | **3.78 0.85 16.77** | **14.00%** | **0.313** |
| Gustafsson, 2007 | 2.12 0.08 53.46 | 50.10% | 0.157 |
| Vasconcelos, 2008 | 5.99 1.47 24.42 | 0.00% | 0.707 |
| Honkaniemi, 2010 | 2.18 0.19 24.84 | 47.20% | 0.169 |
| **OMITTED STUDY: Epstein-Barr virus infection and risk of ALL** |  |  |  |
| **Original effect estimate** | **1.26 0.78 2.03** | **39.50%** | **0.192** |
| Lehtinen, 2003 | 0.94 0.56 1.57 | 0.00% | 0.835 |
| Tedeschi, 2009 | 1.52 0.91 2.55 | 21.90% | 0.258 |
| Francis, 2017 | 1.32 0.67 2.59 | 65.00% | 0.091 |
| **OMITTED STUDY: Urinary tract infection and risk of ALL** |  |  |  |
| **Original effect estimate** | **0.99 0.72 1.36** | **80.20%** | **0.000** |
| McKinney, 1999 | 0.98 0.69 1.37 | 83.30% | 0.000 |
| Naumburg, 2002 | 0.92 0.66 1.26 | 80.30% | 0.000 |
| Kwan, 2007 | 1.05 0.73 1.50 | 83.10% | 0.000 |
| He, 2023 | 0.94 0.66 1.33 | 81.10% | 0.000 |
| Sirirungreung, 2024 | 0.96 0.66 1.40 | 79.30% | 0.000 |
| Bonaventure, 2025 | 1.01 0.67 1.51 | 83.10% | 0.000 |
| Sepulveda, 2025 | 1.10 0.90 1.34 | 30.00% | 0.210 |
| **OMITTED STUDY: Viral infection and risk of ALL** |  |  |  |
| **Original effect estimate** | **1.58 1.15 2.18** | **57.00%** | **0.001** |
| Fedrick, 1972 | 1.42 1.08 1.87 | 43.40% | 0.029 |
| Heinonen, 1973 | 1.57 1.14 2.18 | 59.20% | 0.001 |
| Fine, 1985 | 1.57 1.14 2.17 | 59.10% | 0.001 |
| vanSteensel-Moll, 1985 | 1.61 1.14 2.27 | 59.50% | 0.001 |
| Roman, 1997 | 1.54 1.12 2.13 | 57.80% | 0.002 |
| McKinney, 1999 | 1.63 1.17 2.27 | 59.30% | 0.001 |
| Lehtinen, 2003 | 1.57 1.11 2.24 | 57.70% | 0.002 |
| Gustafsson, 2007 | 1.52 1.11 2.09 | 56.30% | 0.002 |
| Kwan, 2007 | 1.54 1.09 2.18 | 55.20% | 0.003 |
| Vasconcelos, 2008 | 1.60 1.16 2.22 | 59.00% | 0.001 |
| Tedeschi, 2009 | 1.68 1.19 2.36 | 58.10% | 0.001 |
| Honkaniemi, 2010 | 1.55 1.13 2.13 | 57.70% | 0.002 |
| Bogdanovic, 2016 | 1.62 1.17 2.25 | 59.00% | 0.001 |
| Francis, 2017 | 1.47 1.07 2.00 | 52.00% | 0.007 |
| Wang, 2019 | 1.64 1.18 2.28 | 58.70% | 0.001 |
| Heck, 2021 | 1.71 1.23 2.39 | 55.00% | 0.003 |
| Geris, 2023 | 1.61 1.15 2.27 | 59.60% | 0.001 |
| Sirirungreung, 2024 | 1.69 1.18 2.43 | 51.60% | 0.007 |
| **OMITTED STUDY: Influenza infection and risk of ALL** |  |  |  |
| **Original effect estimate** | **3.41 1.28 9.13** | **53.70%** | **0.090** |
| Fedrick , 1972 | 2.06 1.33 3.18 | 0.00% | 0.892 |
| Fine, 1985 | 3.80 1.19 12.10 | 68.60% | 0.041 |
| Roman, 1997 | 3.61 0.94 13.85 | 68.90% | 0.04 |
| Kwan, 2007 | 5.86 1.96 17.53 | 12.90% | 0.317 |
| **OMITTED STUDY: Cytomegalovirus infection and risk of ALL** |  |  |  |
| **Original effect estimate** | **2.34 0.99 5.53** | **73.70%** | **0.010** |
| Fine, 1985 | 1.75 0.92 3.34 | 61.90% | 0.072 |
| Lehtinen, 2003 | 3.69 0.96 14.25 | 72.90% | 0.025 |
| Francis, 2017 | 1.98 0.69 5.67 | 70.10% | 0.035 |
| Geris, 2023 | 3.40 0.94 12.26 | 82.10% | 0.004 |

| **Model Description** | **ES (95% CI)** | **I- squared** | **P value** |
| --- | --- | --- | --- |
| **OMITTED STUDY: Viral infection and risk of solid tumours** |  |  |  |
| **Original effect estimate** | **1.44 0.98 2.10** | **26.40%** | **0.201** |
| Fedrick, 1972 | 1.42 0.95 2.12 | 33.20% | 0.152 |
| Heinonen, 1973 | 1.46 0.97 2.18 | 33.70% | 0.148 |
| McKinney, 1999 | 1.36 0.95 1.94 | 17.10% | 0.291 |
| Shu, 1995 | 1.54 1.12 2.12 | 2.70% | 0.412 |
| Holl, 2008 | 1.32 0.89 1.96 | 22.00% | 0.247 |
| Heck, 2012 | 1.34 0.91 1.97 | 23.10% | 0.238 |
| Heck, 2015 | 1.48 0.95 2.30 | 33.70% | 0.148 |
| Heck, 2021 | 1.49 0.95 2.31 | 33.60% | 0.149 |
| Ma, 2021 | 1.57 1.05 2.36 | 21.80% | 0.249 |
| Sirirungreung, 2024 | 1.43 0.88 2.31 | 33.80% | 0.147 |
| **OMITTED STUDY: Genitourinary tract infection and risk of solid tumours** |  |  |  |
| **Original effect estimate** | **1.60 1.06 2.42** | **63.30%** | **0.005** |
| Swerdlow, 1982 | 1.53 0.98 2.38 | 65.20% | 0.005 |
| Bunin, 1987 | 1.54 1.02 2.33 | 65.20% | 0.005 |
| Olshan, 1993 | 1.81 1.17 2.82 | 58.40% | 0.019 |
| Shu, 1995 | 1.44 0.96 2.18 | 57.90% | 0.02 |
| McKinney, 1999 | 1.55 0.99 2.43 | 65.80% | 0.005 |
| Heck, 2012 | 1.43 0.96 2.12 | 56.30% | 0.025 |
| Heck, 2015 | 1.69 1.09 2.61 | 66.90% | 0.004 |
| He, 2023 | 1.74 1.04 2.94 | 65.60% | 0.005 |
| Sirirungreung, 2024 | 1.77 1.12 2.78 | 64.70% | 0.006 |
| **OMITTED STUDY: Viral infection and risk of CNS tumours & neuroblastomas** |  |  |  |
| **Original effect estimate** | **1.24 0.96 1.60** | **15.90%** | **0.283** |
| Fedrick, 1972 | 1.27 0.96 1.67 | 22.90% | 0.218 |
| Heinonen, 1973 | 1.27 0.96 1.67 | 22.90% | 0.218 |
| Farwell, 1979 | 1.18 0.92 1.52 | 12.80% | 0.319 |
| Fine, 1985 | 1.23 0.95 1.59 | 18.10% | 0.266 |
| Birch, 1990 | 1.26 0.96 1.65 | 22.10% | 0.226 |
| Linos, 1998 | 1.13 0.93 1.38 | 1.30% | 0.431 |
| McKinney, 1999 | 1.27 0.97 1.66 | 22.10% | 0.227 |
| Fear, 2001 | 1.19 0.95 1.50 | 10.30% | 0.344 |
| Hamrik, 2001 | 1.33 1.03 1.70 | 2.60% | 0.419 |
| Stolt, 2004 | 1.31 0.98 1.74 | 20.50% | 0.242 |
| Oksuzyana, 2013 | 1.11 0.91 1.35 | 0.00% | 0.518 |
| Heck, 2021 | 1.30 0.97 1.75 | 22.80% | 0.220 |
| Sirirungreung, 2024 | 1.38 0.97 1.98 | 22.60% | 0.221 |
| **OMITTED STUDY: Urinary tract infection and risk of CNS tumours & neuroblastomas** |  |  |  |
| **Original effect estimate** | **1.10 0.91 1.33** | **0.00%** | **0.941** |
| McKinney, 1999 | 1.11 0.92 1.35 | 0.00% | 0.000 |
| Hamrik, 2001 | 1.07 0.86 1.33 | 0.00% | 0.000 |
| He, 2023 | 1.08 0.87 1.33 | 0.00% | 0.000 |
| Sirirungreung, 2024 | 1.13 0.91 1.42 | 0.00% | 0.000 |
| Bonaventure, 2025 | 1.11 0.90 1.38 | 0.00% | 0.000 |
| **OMITTED STUDY: Viral infection and risk of lymphoma** |  |  |  |
| **Original effect estimate** | **1.11 0.41 3.02** | **50.90%** | **0.087** |
| Fedrick, 1972 | 0.88 0.34 2.31 | 47.70% | 0.125 |
| Roman, 1997 | 1.01 0.31 3.31 | 59.40% | 0.061 |
| McKinney, 1999 | 1.22 0.39 3.79 | 62.90% | 0.044 |
| Heck,2021 | 1.77 0.80 3.89 | 0.00% | 0.673 |
| Bonaventure, 2025 | 1.03 0.26 4.10 | 47.50% | 0.126 |
