# Supplementary material for: Maternal infection during pregnancy and the risk of childhood cancer: a systematic review and meta-analysis
Source: BMC Med. 2026 Jan 14;24:51. doi: 10.1186/s12916-026-04625-1 (PMC12849171; doi:10.1186/s12916-026-04625-1)
Supplement: Supplementary file 7 — Additional file 7: Table. S6: Summary of meta-analyses showing positive associations. Heterogeneity was examined with the I² statistic, categorized as low (≤50%), moderate (>50-75%), or substantial (>75%), with statistical significance assessed using the Q statistic (P-value for heterogeneity).Study quality (maximal score = 45) was stratified above the 4th quintile for quality score (≥28.4 = high quality) and below (<28.4 = low quality). Publication bias was assessed using Egger tests and funnel plots for analyses which had at least 5 studies. Abbreviations. ALL, acute lymphoblastic leukaemia; CI, confidence interval; CMV cytomegalovirus. [file 12916_2026_4625_MOESM7_ESM.docx]

| **Additional file 7: Table. S6: Summary of meta-analyses showing positive associations** | | | | | | | | | | |
| --- | --- | --- | --- | --- | --- | --- | --- | --- | --- | --- |
| **Infection** | **Cancer Outcome** | **All studies** | | | | | **High-quality studies** | | | |
|  |  | **Observed publication bias p value** | **Estimate (95% CI)** | **heterogeneity** | | | **Estimate (95% CI)** | **Heterogeneity** | | |
|  |  |  |  | **%** | **P-value** | **Single study exclusion** |  | **%** | **P-value** | **Single study exclusion** |
| Any infection | All childhood cancers | 0.001 | 1.36 (1.17,1.59) | 71.2 | 0.000 | change in heterogeneity to 56.6 after exclusion of Sepulveda 2025 | 1.25(0.99,1.58) | 64.2 % | 0.003 | 10 high-quality studies; no difference in heterogeneity |
| Viral infection | All childhood cancers | 0.026 | 1.43 (1.18,1.74) | 52.3% | 0.000 | No change in heterogeneity | 1.35(0.95,1.86) | 67.7% | 0.005 | 7 high-quality studies; no Difference in heterogeneity |
| Rubella | All childhood cancers | Not done | 2.04 (1.11,3.75) | 0% | 0.733 | Exclusion of Fine et al drives results positive direction |  |  |  | Only 3 studies of low quality |
| CMV | All childhood cancers | Not done | 2.10 (1.02, 4.34) | 67.1% | 0.010 | No change in heterogeneity | 1.73(0.68,4.39) | 67.4% | 0.01 | 3 high-quality studies; no Difference in heterogeneity |
| Genitourinary tract infection | All childhood cancers |  | 1.52 (1.05, 2.19) | 69.3% | 0.001 | No change in heterogeneity | 2.42(1.50,3.91) | - | - | Only one large cohort study [He 2023] |
| Sexually transmitted infection | All childhood cancers | 0.935 | 2.86 (1.88, 4.33) | 23.6% | 0.249 | 0% heterogeneity after excluding Lehtinen 2005 | 2.44(1.36,4.38) | 49.8% | 0.113 | 4 high-quality studies; 0% heterogeneity after excluding Lehtinen 2005 |
| Genitourinary tract infection | Leukaemia | 0.242 | 1.49 (1.05, 2.12) | 67.1% | 0.006 | No change in heterogeneity | 1.74(1.29,2.35) | - | - | Only one large high-quality cohort study [He 2023] |
| Viral infection | Leukaemia | 0.814 | 1.34(1.00,1.79) | 55.2% | 0.005 | No change in heterogeneity | 1.15(0.72,1.83) | 61.20% | 0.052 | 4 high-quality studies; no difference in heterogeneity; no single study influence on association |
| Viral infection | ALL | 0.112 | 1.58 (1.15, 2.18) | 57.0% | 0.001 | No change in heterogeneity | 1.11(0.84,1.47) | 35.90% | 0.182 | 5 high-quality studies; no difference in heterogeneity; no single study influence on association |
| Influenza | ALL | Not done | 3.41 (1.28, 9.13) | 53.7% | 0.090 | 0% heterogeneity after excluding Fedrick 1972 |  |  |  | Only 4 old studies of low quality |
| Genitourinary tract infection | Solid tumours | 0.177 | 1.60 (1.06, 2.42) | 23.6% | 0.249 | No change in heterogeneity | 1.44 (0.72,2.88) | 74.0% | 0.021 | 3 high-quality studies; Heck 2012 drives association |
| Heterogeneity was examined with the I² statistic, categorized as low (≤50%), moderate (>50-75%), or substantial (>75%), with statistical significance assessed using the Q statistic (P-value for heterogeneity).  Study quality (maximal score = 45) was stratified above the 4th quintile for quality score (≥28.4 = high quality) and below (<28.4 = low quality).  Publication bias was assessed using Egger tests and funnel plots for analyses which had at least 5 studies. Abbreviations. ALL, acute lymphoblastic leukaemia; CI, confidence interval; CMV cytomegalovirus. | | | | | | | | | | |
