# Supplementary material for: Maternal infection during pregnancy and the risk of childhood cancer: a systematic review and meta-analysis
Source: BMC Med. 2026 Jan 14;24:51. doi: 10.1186/s12916-026-04625-1 (PMC12849171; doi:10.1186/s12916-026-04625-1)
Supplement: Supplementary file 8 — Additional file 8: Table. S7: Subgroup analyses of observed associations by study-level characteristics. *P values for the subgroups are calculated using meta-regression, some P values not possible/not done due to few available studies for meta-regression, and some omitted due to collinearity. For exposure assessment, if studies report more than one method of assessment the most valid exposure assessment method is chosen. For outcome assessment, no registry means outcome assessed using methods other than registry or pathological confirmation. For confounder adjustment, basic means matching, or adjustment for basic characteristics e.g., age, sex, region; other factors include adjustments for comorbidities, genetic disorders, proxies for infection exposure such as sibship size, mode of delivery; similar factors include adjustments for other infections and antibiotics. Quality, high ≥ 28.4, cut off and low < 28.4 from the quality assessment tool. Abbreviations: ES estimate; UCI, upper confidence interval; LCI, lower confidence interval; Ref, reference value for subgroup analyses involving three categories. [file 12916_2026_4625_MOESM8_ESM.docx]

**Additional file 8: Table. S7: Subgroup analyses of observed associations by study-level characteristics**

| **Subgroup** | **Category** | **No. of studies** | **ES** | **LCI** | **UCI** | **Cases / Controls** | **Heterogeneity I^2^** | **P value from meta-regression *** |
| --- | --- | --- | --- | --- | --- | --- | --- | --- |
| **Overall maternal infection and risk of overall childhood cancer** | | | | | | | | |
| study period | <2000 | 39 | 1.53 | 1.29 | 1.80 | 28919 / 5327078 | 59.60% | 0.014 |
|  | ≥2000 | 7 | 0.87 | 0.64 | 1.17 | 5135 / 4344882 | 80.70% |  |
| design | case-control | 39 | 1.35 | 1.13 | 1.61 | 27446 / 258928 | 71.20% | 0.046 |
|  | cohort | 7 | 1.43 | 1.02 | 2.00 | 6588 / 9413032 | 72.30% |  |
| exposure assessment | self-report | 14 | 1.42 | 0.98 | 2.07 | 4193 / 21902 | 81.80% | 0.855 |
|  | records | 20 | 1.25 | 1.07 | 1.47 | 26228 / 9638693 | 55.70% | 0.272 |
|  | DNA/antibodies | 12 | 1.56 | 1.07 | 2.29 | 3633 / 11365 | 44.00% | Ref |
| outcome assessment | registry | 41 | 1.38 | 1.17 | 1.62 | 33614 / 9666454 | 73.30% | 0.459 |
|  | no registry | 5 | 1.22 | 0.73 | 2.02 | 440 / 5506 | 39.00% |  |
| confounder adjustment | basic | 28 | 1.51 | 1.22 | 1.87 | 18052 / 314439 | 56.80% | Ref |
|  | other factors | 16 | 1.23 | 0.94 | 1.60 | 13394 / 7280018 | 82.10% | 0.817 |
|  | similar exposures | 2 | 1.25 | 0.65 | 2.39 | 2525 / 2077337 | 85.40% | 0.965 |
| region | Asia/Oceania | 6 | 1.07 | 0.94 | 1.22 | 3636 / 4343594 | 0.00% | Ref |
|  | Europe | 23 | 1.54 | 1.27 | 1.87 | 21002 / 5053819 | 68.00% | 0.930 |
|  | North America | 17 | 1.30 | 0.90 | 1.87 | 9416 / 274547 | 79.90% | 0.782 |
| quality | high | 10 | 1.25 | 0.99 | 1.58 | 13146 / 9562046 | 64.20% | 0.674 |
|  | low | 36 | 1.43 | 1.17 | 1.74 | 20908 / 109914 | 72.20% |  |
| **Infection and risk of leukaemia** | | | | | | | | |
| study period | <2000 | 17 | 1.40 | 1.13 | 1.72 | 10990 / 2264642 | 64.20% | 0.257 |
|  | ≥2000 | 3 | 0.63 | 0.54 | 0.73 | 2297 / 2078464 | 0.00% |  |
| design | case-control | 16 | 1.22 | 0.95 | 1.56 | 11944 / 28116 | 79.50% | 0.746 |
|  | cohort | 4 | 1.70 | 0.82 | 3.53 | 2034 / 4317081 | 79.60% |  |
| exposure assessment | self-report | 5 | 1.32 | 0.57 | 3.05 | 2084 / 19080 | 78.10% | 0.328 |
|  | records | 10 | 1.24 | 1.01 | 1.53 | 9148 / 4316781 | 54.80% | 0.779 |
|  | DNA/antibodies | 5 | 1.33 | 0.93 | 1.90 | 2746 / 9336 | 37.90% | Ref |
| outcome assessment | registry | 18 | 1.26 | 1.00 | 1.60 | 11196 / 4333815 | 81.70% | 0.892 |
|  | no registry | 2 | 1.45 | 0.37 | 5.69 | 138 / 5171 | 0.00% |  |
| confounder adjustment | basic | 10 | 1.60 | 1.05 | 2.45 | 6669 / 37726 | 67.90% | Ref |
|  | other factors | 8 | 1.11 | 0.77 | 1.61 | 6234 / 2230134 | 87.10% | 0.275 |
|  | similar exposures | 2 | 1.19 | 0.54 | 2.63 | 1075 / 2077337 | 83.90% | 0.985 |
| region | Asia/Oceania | 3 | 0.74 | 0.49 | 1.12 | 963 / 2077312 | 0.00% | Ref |
|  | Europe | 14 | 1.43 | 1.14 | 1.79 | 10006 / 2261214 | 66.20% | 0.149 |
|  | North America | 3 | 1.09 | 0.47 | 2.50 | 3009 / 6671 | 91.60% | 0.349 |
| quality | high | 6 | 1.28 | 0.95 | 1.74 | 4716 / 4304601 | 45.50% | 0.457 |
|  | low | 14 | 1.28 | 0.95 | 1.74 | 9262 / 40596 | 82.30% |  |
| **Infection and risk of acute lymphoblastic leukaemia** | | | | | | | | |
| study period | <2000 | 19 | 1.54 | 1.19 | 1.98 | 7015 / 2308384 | 62.80% | 0.009 |
|  | ≥2000 | 4 | 1.05 | 0.81 | 1.36 | 2925 / 4344454 | 90.90% |  |
| design | case-control | 17 | 1.30 | 0.97 | 1.73 | 7549 / 19103 | 78.60% | 0.014 |
|  | cohort | 6 | 1.37 | 0.90 | 2.08 | 2391 / 6633735 | 69.00% |  |
| exposure assessment | self-report | 5 | 1.56 | 0.65 | 3.75 | 2438 / 19435 | 91.40% | 0.455 |
|  | records | 9 | 1.57 | 0.97 | 2.54 | 4326 / 6623544 | 44.30% | 0.068 |
|  | DNA/antibodies | 9 | 1.63 | 0.93 | 2.85 | 3176 / 9859 | 50.60% | Ref |
| outcome assessment | registry | 21 | 1.26 | 1.01 | 1.59 | 9889 / 6647748 | 78.70% | 0.505 |
|  | no registry | 2 | 5.12 | 1.25 | 20.87 | 51 / 5090 | 0.00% |  |
| confounder adjustment | basic | 12 | 2.21 | 1.27 | 3.85 | 2643 / 80110 | 70.00% | Ref |
|  | other factors | 9 | 1.05 | 0.77 | 1.42 | 4015 / 4489242 | 0.00% | 0.859 |
|  | similar exposures | 2 | 1.25 | 0.47 | 3.30 | 840 / 2077275 | 85.80% | 0.764 |
| region | Asia/Oceania | 3 | 1.05 | 0.81 | 1.36 | 1672 / 2266651 | 16.80% | Ref |
|  | Europe | 14 | 1.40 | 1.07 | 1.82 | 5156 / 2251987 | 61.40% | 0.147 |
|  | North America | 6 | 1.45 | 0.62 | 3.39 | 3112 / 57852 | 88.30% | 0.195 |
| quality | high | 7 | 1.16 | 0.99 | 1.37 | 4810 / 6569952 | 13.60% | 0.101 |
|  | Low | 16 | 1.55 | 1.09 | 2.22 | 5130 / 82886 | 82.10% |  |
| **Infection and risk of solid tumours** | | | | | | | | |
| study period | <2000 | 12 | 1.46 | 1.06 | 2.02 | 2442 / 2517060 | 49.70% | 0.763 |
|  | ≥2000 | 4 | 1.18 | 0.89 | 1.58 | 549 / 4342950 | 0.00% |  |
| design | case-control | 10 | 1.38 | 0.95 | 1.99 | 1104 / 226279 | 60.30% | 0.543 |
|  | cohort | 6 | 1.27 | 0.99 | 1.61 | 1887 / 6633731 | 0.00% |  |
| exposure assessment | self-report | 6 | 0.89 | 0.62 | 1.27 | 642 / 17986 | 7.50% | 0.792 |
|  | records | 9 | 1.44 | 1.14 | 1.82 | 2320 / 6841909 | 24.30% | 0.363 |
|  | DNA/antibodies | 1 | 2.72 | 1.05 | 7.04 | 29 / 115 | na | Ref |
| outcome assessment | registry | 14 | 1.36 | 1.05 | 1.75 | 2820 / 6854835 | 43.10% | 0.588 |
|  | no registry | 2 | 1.08 | 0.61 | 1.92 | 171 / 5175 | 0.00% |  |
| confounder adjustment | basic | 10 | 1.70 | 1.15 | 2.51 | 773 / 297986 | 48.90% | Ref |
|  | other factors | 5 | 1.13 | 0.90 | 1.41 | 2050 / 4485147 | 0.00% | 0.071 |
|  | similar exposures | 1 | 1.40 | 0.56 | 3.52 | 168 / 2076877 | na | 0.217 |
| region | Asia/Oceania | 3 | 1.24 | 0.89 | 1.73 | 384 / 4342814 | 0.00% | Ref |
|  | Europe | 7 | 1.42 | 1.09 | 1.85 | 1853 / 2256176 | 18.70% | 0.849 |
|  | North America | 6 | 1.22 | 0.62 | 2.41 | 754 / 261020 | 64.70% | 0.801 |
| quality | high | 4 | 1.49 | 1.02 | 2.20 | 2055 / 6770140 | 51.30% | 0.117 |
|  | low | 12 | 1.24 | 0.92 | 1.684 | 936/89870 | 34.80% |  |

| **Subgroup** | **Category** | **No. of studies** | **ES** | **LCI** | **UCI** | **Cases / Controls** | **Heterogeneity I^2^** | **P value from meta-**  **regression *** |
| --- | --- | --- | --- | --- | --- | --- | --- | --- |
| **Sexually transmitted infection and risk of childhood cancer** | | | | | | | | |
| design | case-control | 6 | 2.76 | 1.58 | 4.82 | 4879 / 214761 | 35.50% | Omitted |
|  | cohort | 1 | 3.13 | 1.73 | 5.67 | 1307 / 2218435 | na |  |
| exposure assessment | self-report | 2 | 3.73 | 1.83 | 7.64 | 691 / 209555 | 0.00% | Omitted |
|  | records | 4 | 3.19 | 2.10 | 4.84 | 4915 / 2431020 | 0.00% |  |
|  | DNA/antibodies | 1 | 0.70 | 0.20 | 2.00 | 402 / 1212 | na |  |
| confounder adjustment | basic | 3 | 3.39 | 1.93 | 5.97 | 3925 / 4038 | 0.00% | Omitted |
|  | other factors | 3 | 2.06 | 0.93 | 4.57 | 5017 / 2222955 | 62.00% |  |
|  | similar exposures | 1 | 7.59 | 1.58 | 36.56 | 365 / 460 | na |  |
| region | Europe | 3 | 1.91 | 0.57 | 6.46 | 1822 / 2219873 | 63.50% | Omitted |
|  | North America | 4 | 3.40 | 2.15 | 5.38 | 4364 / 213323 | 0.00% |  |
| quality | high | 4 | 2.44 | 1.36 | 4.38 | 5204 / 2432006 | 49.80% | Omitted |
|  | low | 3 | 3.82 | 1.90 | 7.69 | 982 / 1190 | 0.00% |  |
| **Viral infection and risk of childhood cancer** | | | | | | | | |
| study period | <2000 | 32 | 1.59 | 1.26 | 1.99 | 24894 / 2886599 | 51.10% | 0.661 |
|  | ≥2000 | 5 | 1.00 | 0.84 | 1.18 | 3548 / 4343295 | 0.00% |  |
| design | case-control | 32 | 1.44 | 1.17 | 1.76 | 23175 / 40328 | 40.10% | 0.053 |
|  | cohort | 5 | 1.51 | 0.87 | 2.64 | 5267 / 7189566 | 78.30% |  |
| exposure assessment | self-report | 16 | 1.36 | 1.01 | 1.84 | 14292 / 87641 | 49.40% | 0.788 |
|  | records | 10 | 1.45 | 1.02 | 2.04 | 10919 / 7132100 | 57.50% | 0.414 |
|  | DNA/antibodies | 11 | 1.53 | 1.00 | 2.33 | 3231 / 10153 | 49.00% | Ref |
| outcome assessment | registry | 33 | 1.41 | 1.16 | 1.72 | 28134 / 7224520 | 53.80% | 0.090 |
|  | no registry | 4 | 1.97 | 0.79 | 4.91 | 308 / 5374 | 44.60% |  |
| confounder adjustment | basic | 23 | 1.64 | 1.24 | 2.16 | 16339 / 94293 | 44.20% | Ref |
|  | other factors | 12 | 1.27 | 0.89 | 1.80 | 9578 / 5058264 | 57.10% | 0.403 |
|  | similar exposures | 2 | 1.25 | 0.65 | 2.39 | 2525 / 2077337 | 85.40% | 0.565 |
| region | Asia/Oceania | 5 | 0.97 | 0.83 | 1.15 | 3504 / 4343462 | 0.00% | Ref |
|  | Europe | 22 | 1.71 | 1.31 | 2.23 | 18869 / 2825348 | 39.80% | 0.053 |
|  | North America | 10 | 1.51 | 0.97 | 2.35 | 6069 / 61084 | 58.50% | 0.068 |
| quality | high | 7 | 1.33 | 0.95 | 1.86 | 10828 / 7133348 | 67.70% | 0.536 |
|  | low | 30 | 1.48 | 1.17 | 1.89 | 17614 / 96546 | 45.50% |  |
| **Rubella virus infection and risk of childhood cancer** | | | | | | | | |
| design | case-control | 2 | 2.17 | 1.16 | 4.06 | 11823 / 11484 | 0.00% | - |
|  | cohort | 1 | 0.86 | 0.24 | 31.10 | 14 / 3244 | na | - |
| outcome assessment | registry | 2 | 2.17 | 1.16 | 4.06 | 11823 / 11484 | 0.00% | - |
|  | no registry | 1 | 0.86 | 0.24 | 31.10 | 14 / 3244 | na | - |
| **Cytomegalovirus infection and risk of childhood cancer** | | | | | | | | |
| design | case-control | 4 | 1.5 | 0.77 | 2.93 | 1926 / 6500 | 60.80% | 0.034 |
|  | cohort | 2 | 6.62 | 2.00 | 21.95 | 2279 / 2781867 | 0.00% |  |
| exposure assessment | records | 2 | 6.62 | 2.00 | 21.95 | 2279 / 2781867 | 0.00% | Omitted |
|  | DNA/antibodies | 4 | 1.5 | 0.77 | 2.93 | 1926 / 6500 | 60.80% |  |
| outcome assessment | registry | 5 | 1.89 | 0.87 | 4.11 | 4191 / 2785809 | 69.30% | 0.360 |
|  | no registry | 1 | 4.32 | 0.95 | 19.58 | 14 / 2558 | na |  |
| confounder adjustment | basic | 3 | 2.84 | 1.12 | 7.21 | 348 / 3086 | 30.30% | Omitted |
|  | other factors | 3 | 1.73 | 0.68 | 4.39 | 3857 / 2785281 | 67.40% |  |
| region | Europe | 4 | 2.22 | 0.66 | 7.39 | 2748 / 2783341 | 67.80% | Omitted |
|  | USA | 2 | 2.24 | 0.80 | 6.25 | 1457 / 5026 | 64.00% |  |
| quality | high | 3 | 1.73 | 0.68 | 4.39 | 3857 / 2785281 | 67.40% | 0.606 |
|  | low | 3 | 2.84 | 1.12 | 7.21 | 348 / 3086 | 30.30% |  |
| **Genitourinary tract infection and risk of childhood cancer** | | | | | | | | |
| design | case-control | 8 | 1.37 | 0.95 | 1.97 | 4592 / 18945 | 61.50% | 0.744 |
|  | cohort | 1 | 2.42 | 1.5 | 3.92 | 1307 / 2218435 | na |  |
| exposure assessment | self-report | 3 | 1.00 | 0.57 | 1.76 | 1743 / 1776 | 34.90% | 0.658 |
|  | records | 6 | 1.77 | 1.11 | 2.81 | 4156 / 2235604 | 75.30% |  |
| confounder adjustment | basic | 6 | 1.406 | 0.864 | 2.29 | 2485 / 16838 | 62.10% | 0.943 |
|  | other factors | 3 | 1.72 | 1.12 | 2.65 | 3414 / 2220542 | 53.40% |  |
| region | Europe | 6 | 1.77 | 1.11 | 2.81 | 4156 / 2235604 | 75.30% | Omitted |
|  | North America | 3 | 1.00 | 0.57 | 1.76 | 1743 / 1776 | 34.90% |  |
| quality | high | 1 | 2.42 | 1.5 | 3.92 | 1307 / 2218435 | na | Omitted |
|  | low | 8 | 1.37 | 0.95 | 1.97 | 4592 / 18945 | 61.50% |  |
| **Genitourinary tract infection and risk of leukaemia** | | | | | | | | |
| study design | case-control | 6 | 1.45 | 0.95 | 2.22 | 4582 / 8956 | 64.00% | 0.016 |
|  | cohort | 1 | 1.74 | 1.29 | 2.35 | 1307 / 2218435 | na |  |
| exposure assessment | self-reports | 2 | 2.45 | 0.35 | 17.03 | 1820 / 1915 | 81.10% | 0.029 |
|  | records | 5 | 1.45 | 1.01 | 2.09 | 4069 / 2225476 | 69.00% |  |
| confounder adjustment | basic | 3 | 1.14 | 0.73 | 1.8 | 2110 / 6389 | 34.90% | Ref |
|  | other factors | 3 | 1.63 | 1.27 | 2.09 | 3414 / 2220542 | 0.00% | 0.261 |
|  | similar exposures | 1 | 7.59 | 1.58 | 36.56 | 365 / 460 | na | Omitted |
| region | Europe | 5 | 1.45 | 1.01 | 2.09 | 4069 / 2225476 | 69.00% | Omitted |
|  | North America | 2 | 2.45 | 0.35 | 17.03 | 1820 / 1915 | 81.10% |  |
| quality | high quality | 1 | 1.74 | 1.29 | 2.35 | 1307 / 2218435 | na | Omitted |
|  | low quality | 6 | 1.45 | 0.95 | 2.35 | 4582 / 8956 | 64.00% |  |

| **Subgroup** | **Category** | **No. of studies** | **ES** | **LCI** | **UCI** | **Cases / Controls** | **Heterogeneity I^2^** | **P value from meta- regression*** |
| --- | --- | --- | --- | --- | --- | --- | --- | --- |
| **Viral infection and risk of acute lymphoblastic leukaemia** | | | | | | | | |
| study period | < 2000 | 15 | 1.94 | 1.36 | 2.78 | 3916 / 82644 | 40.20% | 0.063 |
|  | ≥ 2000 | 3 | 1.01 | 0.83 | 1.24 | 1672 / 4342999 | 0.00% |  |
| study design | case-control | 13 | 1.64 | 1.21 | 2.24 | 1341 / 4415284 | 27.30% | 0.018 |
|  | cohort | 5 | 1.74 | 0.77 | 3.93 | 4247 / 10359 | 74.80% |  |
| exposure assessment | self-report | 4 | 2.11 | 0.95 | 4.69 | 1185 / 17980 | 71.00% | 0.535 |
|  | records | 6 | 1.05 | 0.86 | 1.28 | 1568 / 4399016 | 0.00% | 0.298 |
|  | biological specimen | 8 | 1.73 | 1.05 | 2.85 | 2835 / 8647 | 43.00% | Ref |
| outcome assessment | no registry | 2 | 4.78 | 1.17 | 19.48 | 51 / 5074 | 0.00% | 0.643 |
|  | registry/pathological | 16 | 1.51 | 1.09 | 2.08 | 5537 / 4420569 | 58.60% |  |
| confounder adjustment | basic | 11 | 2.60 | 1.44 | 4.71 | 1513 / 74595 | 38.80% | Ref |
|  | other factors | 5 | 1.17 | 0.91 | 1.50 | 3235 / 2273773 | 16.10% | 0.588 |
|  | similar exposures | 2 | 1.25 | 0.47 | 3.30 | 840 / 2077275 | 85.80% | 0.865 |
| region | Asia & Oceania | 3 | 1.01 | 0.83 | 1.24 | 1672 / 4342999 | 0% | Ref |
|  | Europe | 10 | 1.98 | 1.16 | 3.36 | 2067 / 26247 | 52.00% | 0.861 |
|  | North America | 5 | 2.09 | 1.46 | 3.00 | 1859 / 56397 | 0% | Omitted |
| quality | high quality | 5 | 1.11 | 0.84 | 1.47 | 3419 / 4350305 | 35.90% | 0.277 |
|  | low quality | 13 | 2.22 | 1.40 | 3.51 | 2169 / 75338 | 38.20% |  |
| **Influenza virus infection and risk of acute lymphoblastic leukaemia** | | | | | | | | |
| study design | case-control | 2 | 2.07 | 1.33 | 3.22 | 424 / 624 | 0.00% | Omitted |
|  | cohort | 2 | 6.80 | 1.21 | 38.21 | 12 / 21757 | 30.30% |  |
| exposure assessment | self-report | 2 | 4.31 | 0.80 | 23.13 | 321 / 17128 | 84.20% | Omitted |
|  | records | 2 | 2.54 | 0.54 | 12.03 | 115 / 5253 | 0.00% |  |
| outcome assessment | no registry | 1 | 1.50 | 0.07 | 31.90 | 2 / 5027. | na | Omitted |
|  | registry/pathological | 3 | 3.80 | 1.19 | 12.10 | 434 / 17364 | 68.60% |  |
| confounder adjustment | basic | 3 | 5.86 | 1.96 | 17.53 | 125 / 21983 | 12.90% | Omitted |
|  | similar exposures | 1 | 2.02 | 1.28 | 3.18 | 311 / 398 | na |  |
| Region | Europe | 3 | 5.86 | 1.96 | 17.53 | 125 / 21983 | 12.90% | Omitted |
|  | North America | 1 | 2.02 | 1.28 | 3.18 | 311 / 398 | na |  |
| **Genitourinary tract infection and risk of solid tumours** | | | | | | | | |
| study period | < 2000 | 7 | 1.89 | 1.17 | 3.07 | 2257 / 2438804 | 68.60% | 0.410 |
|  | ≥ 2000 | 2 | 0.83 | 0.44 | 1.56 | 301 / 2265913 | 0.00% |  |
| design | case-control | 7 | 1.98 | 1.12 | 3.52 | 858 / 220505 | 65.10% | 0.000 |
|  | cohort | 2 | 1.10 | 0.81 | 1.49 | 1700 / 4484212 | 0.00% |  |
| exposure assessment | self-report | 4 | 1.56 | 0.62 | 3.96 | 558 / 1096 | 72.50% | 0.019 |
|  | records | 5 | 1.67 | 1.02 | 2.75 | 2000 / 4703621 | 62.50% |  |
| outcome assessment | registry/pathological | 8 | 1.69 | 1.09 | 2.61 | 2393 / 4704581 | 66.90% | 0.247 |
|  | no registry | 1 | 0.80 | 0.20 | 3.00 | 165 / 136 | N/A |  |
| confounder adjustment | basic | 5 | 2.06 | 1.01 | 4.22 | 588 / 219730 | 69.90% | 0.006 |
|  | other factors | 4 | 1.31 | 0.75 | 2.26 | 1970 / 4484987 | 60.20% |  |
| region | Asia & Oceania | 1 | 0.84 | 0.41 | 1.71 | 136 / 2265777 | N/A | Ref |
|  | Europe | 3 | 1.60 | 0.96 | 2.67 | 1677 / 10358 | 44.80% | Omitted |
|  | North America | 5 | 1.89 | 0.83 | 4.30 | 745 / 210147 | 74.90% | Omitted |
| quality | high | 3 | 1.44 | 0.72 | 2.88 | 1887 / 4693263 | 74.00% | 0.398 |
|  | low | 6 | 1.77 | 0.96 | 3.27 | 671 / 11454 | 63.00% |  |

*P values for the subgroups are calculated using meta-regression, some P values not possible/not done due to few available studies for meta-regression, and some omitted due to collinearity. For exposure assessment, if studies report more than one method of assessment the most valid exposure assessment method is chosen. For outcome assessment, no registry means outcome assessed using methods other than registry or pathological confirmation. For confounder adjustment, basic means matching, or adjustment for basic characteristics e.g., age, sex, region; other factors include adjustments for comorbidities, genetic disorders, proxies for infection exposure such as sibship size, mode of delivery; similar exposures include adjustments for other infections and antibiotics. Quality, high ≥ 28.4, cut off and low < 28.4 from the quality assessment tool. Abbreviations: ES estimate; UCI, upper confidence interval; LCI, lower confidence interval; Ref, reference value for subgroup analyses involving three categories.
