# Supplementary figures and images for: Maternal infection during pregnancy and the risk of childhood cancer: a systematic review and meta-analysis
Source: BMC Med. 2026 Jan 14;24:51. doi: 10.1186/s12916-026-04625-1 (PMC12849171; doi:10.1186/s12916-026-04625-1)

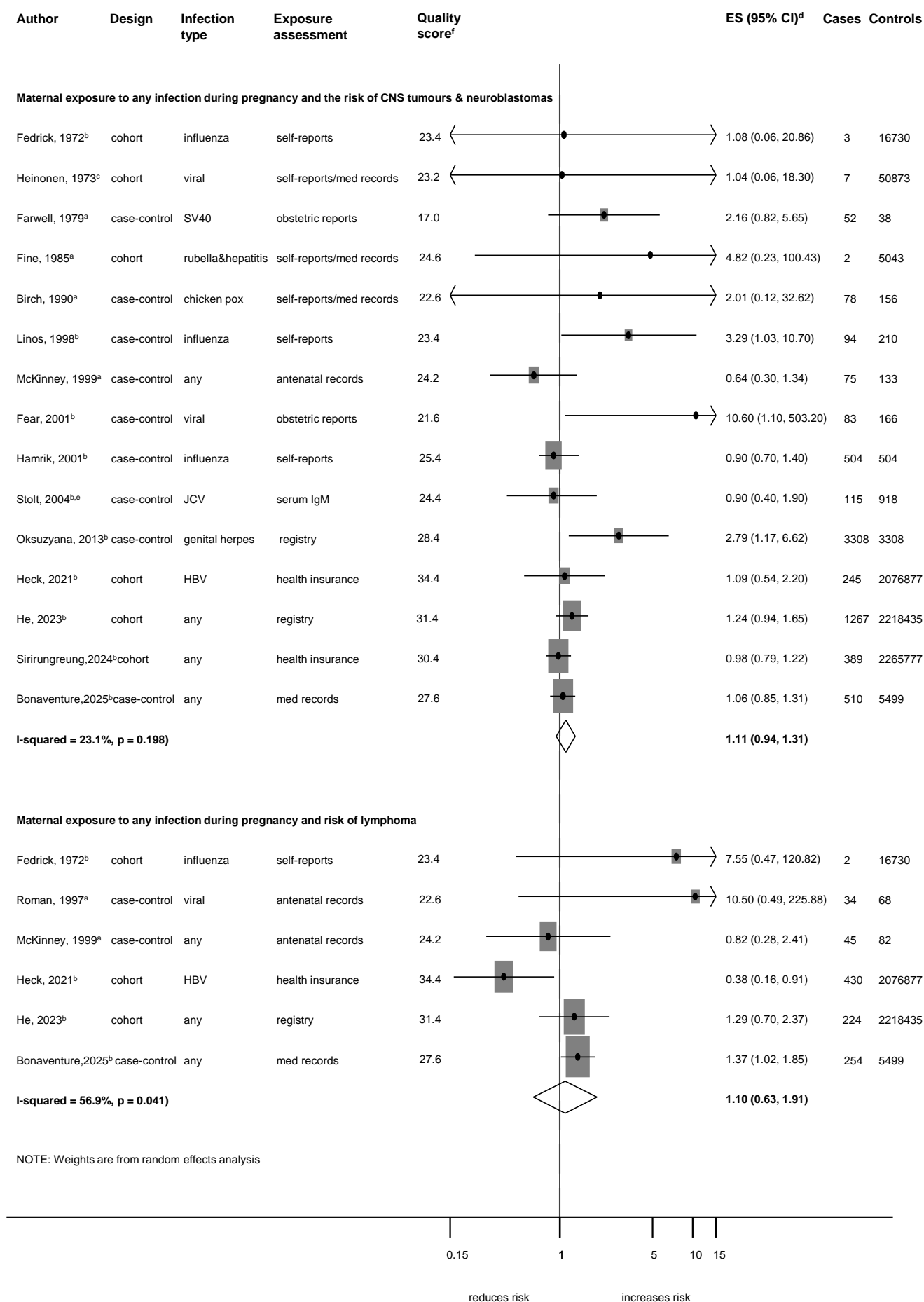

Supplement: Supplementary file 9 — Additional file 9: Fig.S2: Maternal exposure to any infection during pregnancy and the risk of CNS tumours and neuroblastomas and lymphoma. Lymphoma includes all subtypes. a) crude odds ratio taking matching into account. (b) Adjusted estimate as indicated by published study. (c) no adjustment/matching. (d) ES includes single-study odds ratios or hazard ratios and summary odds ratios. (e) first-trimester maternal serum samples. (f) quality score, high quality ≥28.4, low quality <28.4. Abbreviations: CNS, central nervous system; CI, confidence interval; ES, estimate; IgM, immunoglobulin M; JCV, human polyomavirus 2; SV 40, simian virus 40. med records, medical records. Weights are from random effect analysis. [file 12916_2026_4625_MOESM9_ESM.pdf]

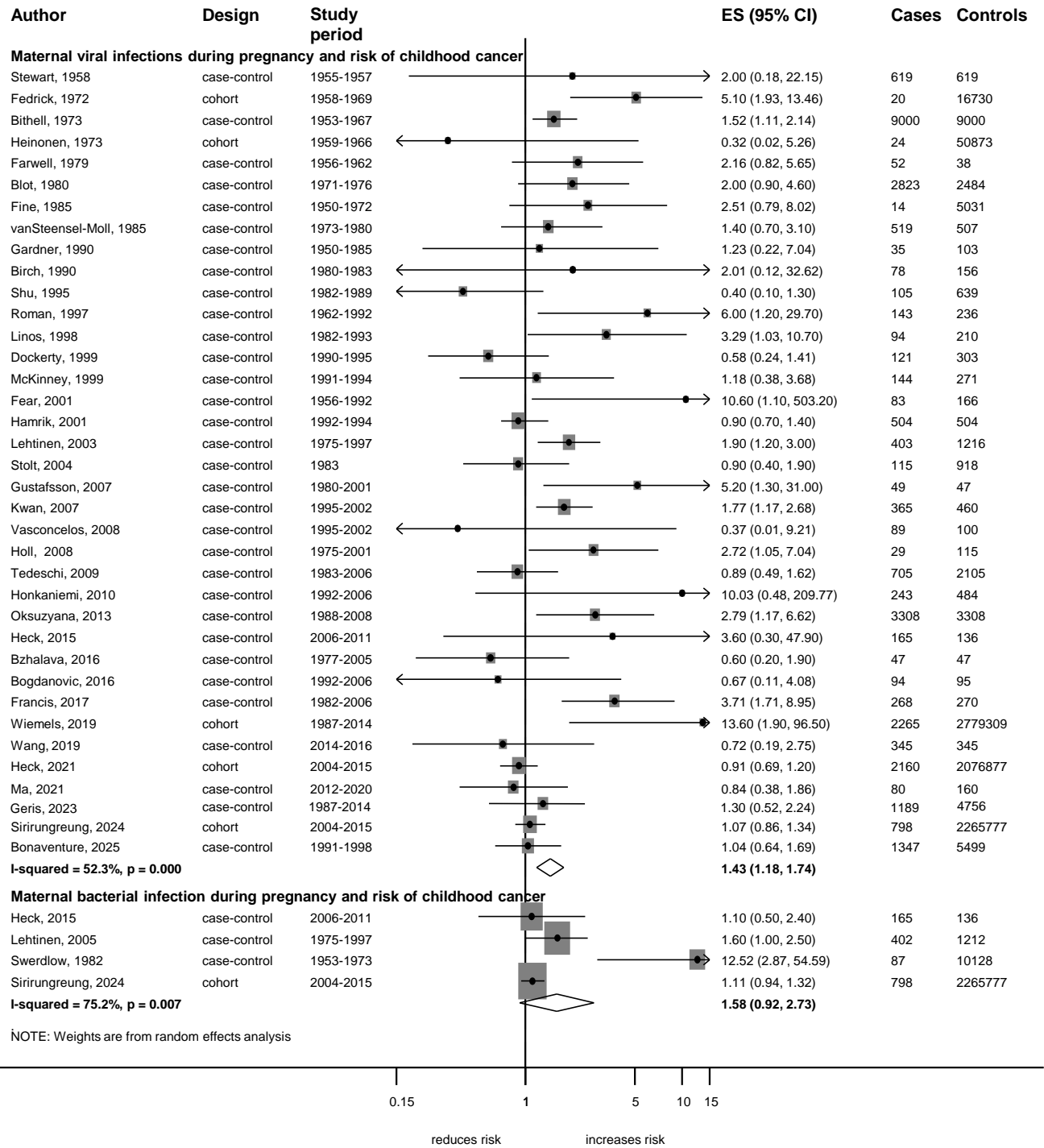

Supplement: Supplementary file 10 — Additional file 10: Fig.S3: Maternal Infection by type of pathogen – viral and bacterial, and overall childhood cancer risk. Abbreviations: ES, estimate; CI confidence interval. [file 12916_2026_4625_MOESM10_ESM.pdf]

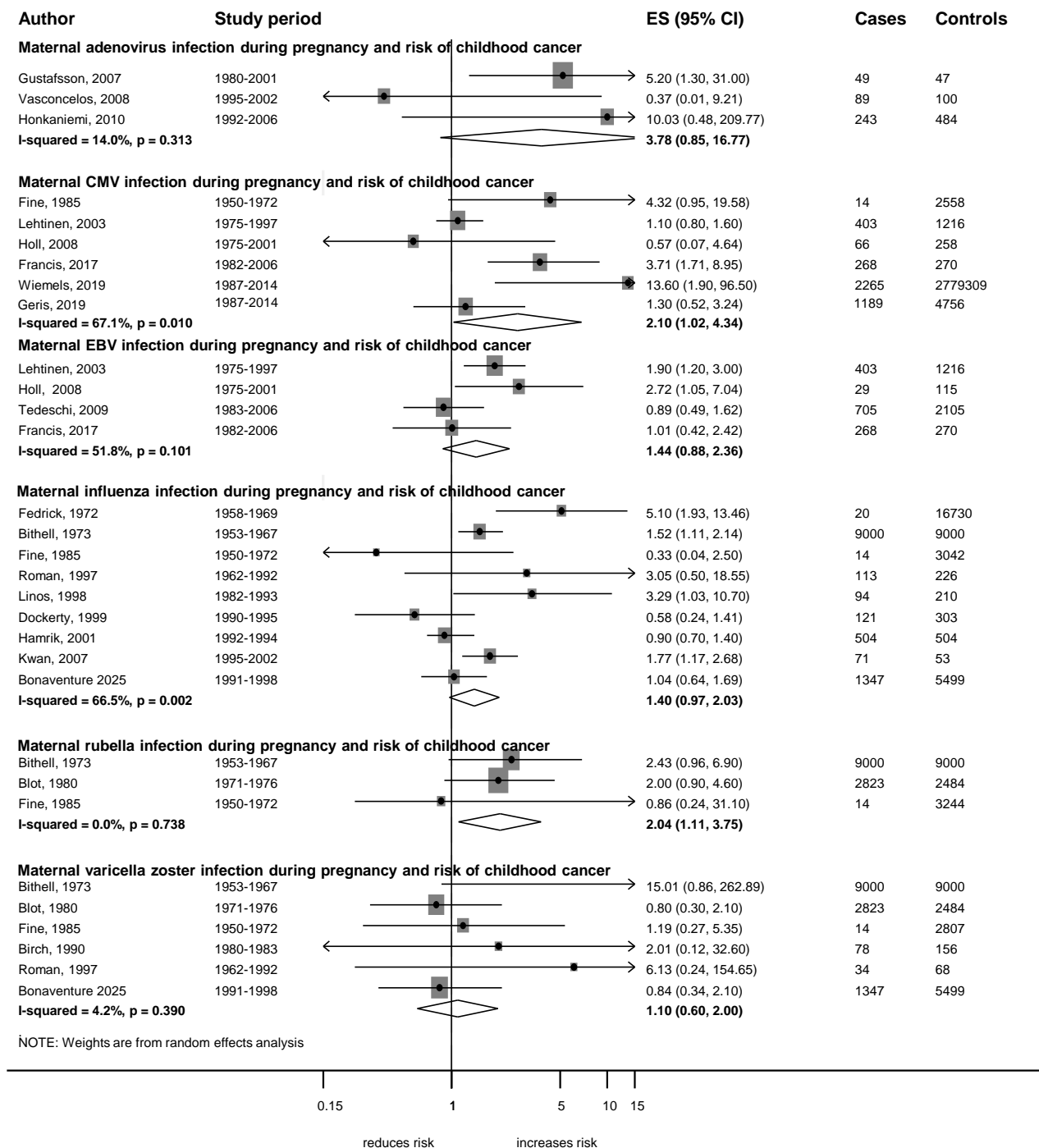

Supplement: Supplementary file 11 — Additional file 11: Fig.S4: Specific viral infections and overall childhood cancer risk. Abbreviations: CMV, cytomegalovirus; EBV, epstein-barr virus; ES, estimate. CI, confidence interval. [file 12916_2026_4625_MOESM11_ESM.pdf]

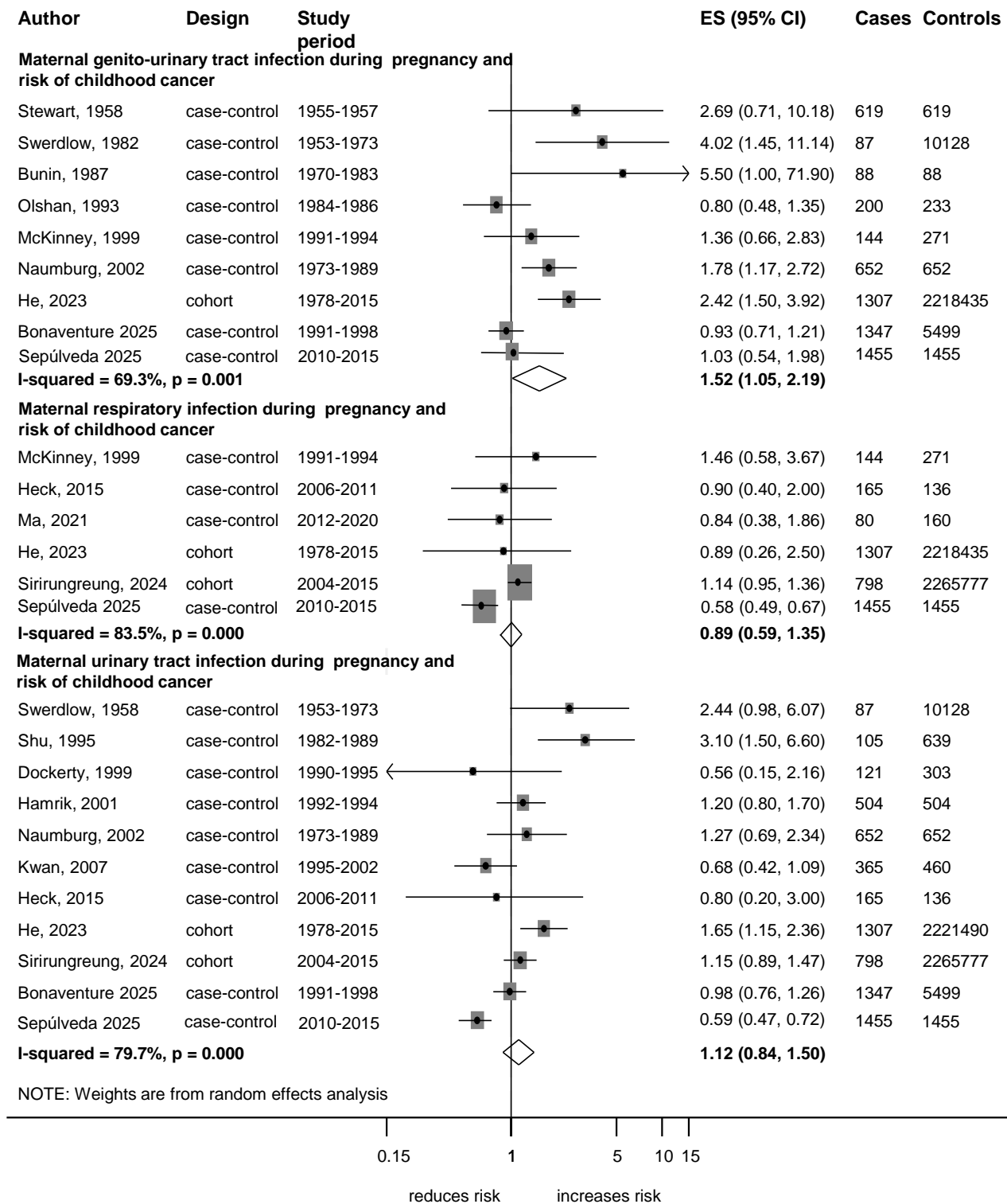

Supplement: Supplementary file 12 — Additional file 12: Fig.S5: Maternal Infection by affected body system and overall childhood cancer risk. Abbreviations: ES, estimate; CI, confidence interval. [file 12916_2026_4625_MOESM12_ESM.pdf]

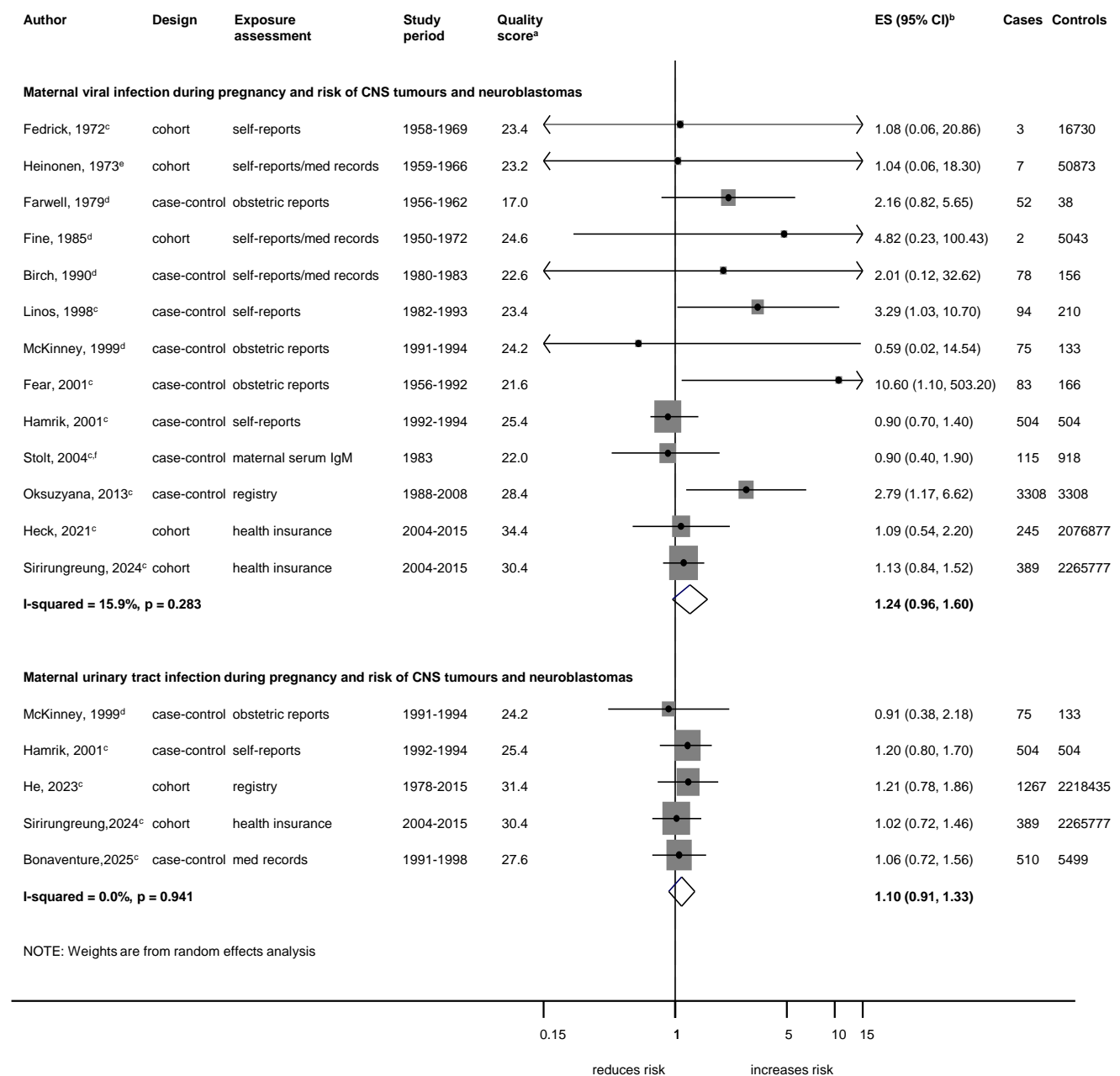

Supplement: Supplementary file 13 — Additional file 13: Fig.S6: Specific maternal infections during pregnancy and the risk of central nervous system tumours and neuroblastomas. (a) quality score, high quality ≥28.4, low quality <28.4. (b) ES includes single-study odds ratios or hazard ratios and summary odds ratios. (c) Adjusted estimate as indicated by published study. (d) crude odds ratio taking matching into account. (e) no adjustment/matching. (f) first-trimester maternal serum samples. Abbreviations: CNS, central nervous system; ES, estimate; CI, confidence interval; IgM, Immunoglobulin M; IgG, Immunoglobulin G. Weights are from random effect analysis. [file 12916_2026_4625_MOESM13_ESM.pdf]

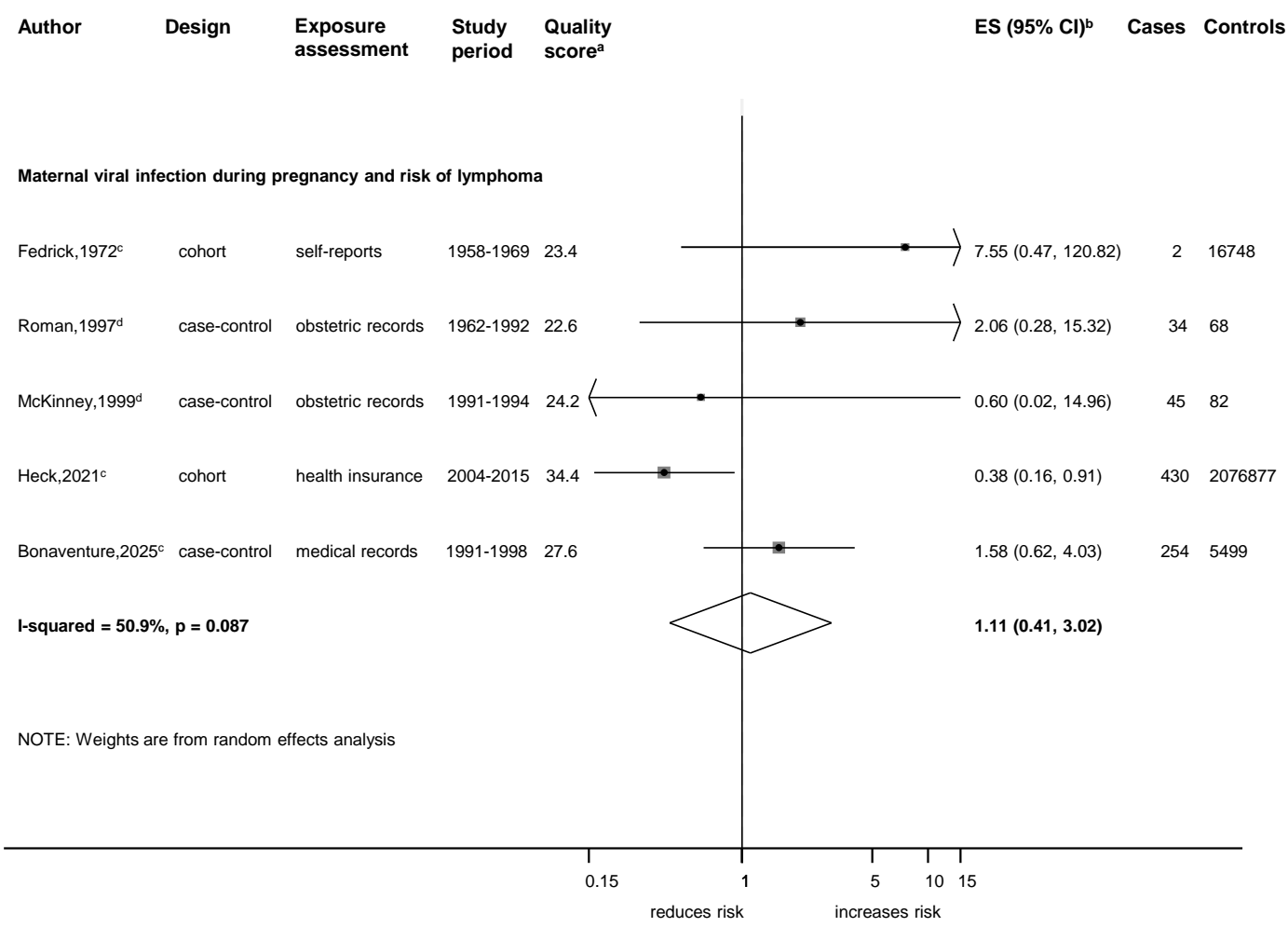

Supplement: Supplementary file 14 — Additional file 14: Fig.S7: Maternal viral infection during pregnancy and the risk of lymphoma. Lymphoma includes all subtypes. (a) quality score, high quality ≥28.4, low quality <28.4. (b) ES includes single-study odds ratios or hazard ratios and summary odds ratios. (c) Adjusted estimate as indicated by published study. (d) crude odds ratio taking matching into account. Abbreviations: ES, estimate; CI, confidence interval. Weights are from random effect analysis. [file 12916_2026_4625_MOESM14_ESM.pdf]
